# Symmetrization of Strong Hydrogen Bond under High Pressure in Bihydroxide-Ion-Containing NaCu_2_(SO_4_)_2_·H_3_O_2_ Revealed by Experimental Charge Density, Single-Crystal Electron Diffraction, and Neutron Diffraction Studies

**DOI:** 10.1021/jacs.5c08310

**Published:** 2025-07-17

**Authors:** Piotr Rejnhardt, Roman Gajda, Magdalena Woińska, Jan Parafiniuk, Gerald Giester, Ronald Miletich, Yan Wu, Tomasz Poręba, Mohamed Mezouar, Szymon Sutuła, Tomasz Góral, Przemysław Dera, Krzysztof Woźniak

**Affiliations:** † Department of Chemistry, 272377 University of Warsaw, Pasteura 1, Warszawa 02-093, Poland; ‡ Department of Geochemistry, Mineralogy and Petrology, Faculty of Geology, 272377 University of Warsaw, Żwirki i Wigury 93, 02-089 Warszawa, Poland; § Department of Mineralogy and Crystallography, University of Vienna, Josef-Holaubek-Platz 2, Vienna A-1090, Austria; ∥ Neutron Scattering Division, 6146 Oak Ridge National Laboratory, Oak Ridge, Tennessee 37831, United States; ⊥ ID27 High Pressure Beamline, 55553 European Synchrotron Radiation Facility (ESRF), 71 avenue des Martyrs, 38000 Grenoble, France; # Centre of New Technologies, University of Warsaw, S. Banacha 2c, 02-097 Warszawa, Poland; ∇ Hawai’i Institute of Geophysics and Planetology, University of Hawai’i at Manoa, 1680 East-West Road, Honolulu, Hawaii 96822, United States

## Abstract

In minerals and inorganic compounds, strong hydrogen bonding can lead to the formation of complex ionic species such as the H_3_O_2_^–^ bihydroxide anion and Zundel cation H_5_O_2_^+^. We studied [NaCu_2_(SO_4_)_2_·H_3_O_2_] natrochalcite, which contains bihydroxide anions and undergoes hydrogen bond symmetrization at the lowest pressure reported so far among inorganic compounds. Hydrogen bond symmetrization leads to changes in the bulk modulus, seismic wave velocities, and proton mobility and plays a primary role in high-temperature superconductivity, but its characteristics are not well understood due to a lack of systematic studies and limitations of experimental methods sensitive to this subtle change. In this work, we applied experimental charge density analysis based on in situ single-crystal X-ray diffraction data, along with the single-crystal neutron and electron diffraction experiments, to probe the behavior of hydrogen atoms during the hydrogen bond symmetrization process under high-pressure conditions. On the way to the symmetrical H-bonding, natrochalcite undergoes a series of complex redistributions of electron density, which we trace with multipole refinement and detailed analysis of changes in the Laplacian of electron density values. Additionally, we deconvoluted the equation of state (volume of the unit cell vs pressure relation) into the atomic equation of states describing dependencies of atomic charges or volumes vs pressure.

## Graphical abstract

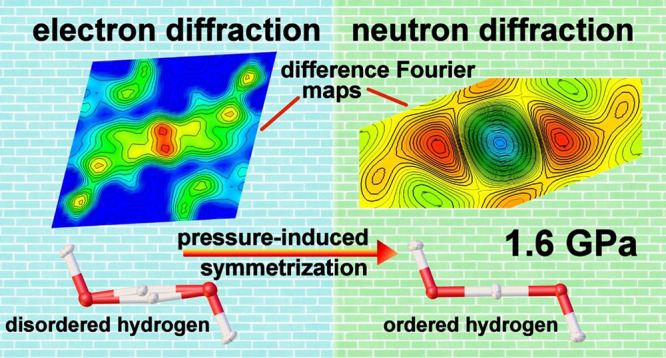

## 1 Introduction

Our understanding of the water cycle in the Earth’s interior has significantly changed over the past decade. For many years, it was believed that hydrous minerals are absent in the lower mantle, because high-temperature and high-pressure conditions lead to their dehydration and water release.^1^ However, recent high-pressure research using diamond anvil cells (DACs)^2^ revealed examples of minerals containing water, such as dense hydrous magnesium silicates,^3,4^ δ-AlOOH,^5^ or ε-FeOOH,^6^ which remain stable under lower mantle conditions. In these hydrous mineral phases, hydrogen bonds (HBs) play a critical role in controlling the compression behavior and physical properties.

In minerals, most hydrogens are bonded to oxygen, making it common to consider the oxygen and its associated hydrogen atoms as a defined unit or species (e.g., OH^–^, H_2_O^0^, and H_3_O^+^). Larger structures, such as the H_3_O_2_^–^ bihydroxide anion (also referred to as the Zundel anion^7^), the Zundel cation H_5_O_2_^+^, and the Eigen cation H_9_O_4_^+^ are stabilized by strong HBs, where the strength of these bonds is related to the O–H···O donor–acceptor distance.^8,9^ Bonds with an O···O distance shorter than 2.5 Å are considered very strong and are intrinsic to both the H_3_O_2_^–^ and H_5_O_2_^+^ units.

Upon compression, all of the hydrous minerals, as well as other inorganic and molecular solids featuring HBs, exhibit a peculiar common transformation involving hydrogen atom. At pressure values corresponding roughly to lower mantle depths, the distinction between the donor and the acceptor of the HB disappears, and the bond becomes symmetric.^5,10–13^ Previous studies of HB symmetrization in inorganic structures mainly utilized first-principles calculations,^5,12,13^ spectroscopic measurements,^11,14,15^ and conventional in situ powder X-ray and neutron diffraction.^10,16^ This currently available toolset has some limitations. First-principles calculations rely on approximations that limit their accuracy in capturing the complex interactions of hydrogen bonding in inorganic structures, while spectroscopic techniques may struggle to provide detailed structural insights into bonding rearrangements. Powder X-ray and neutron diffraction using the independent atom model (IAM) yield average structural information but overlook critical details regarding HBs due to the assumption of spherical atoms. These limitations underscore the need for more sophisticated methods to accurately assess subtleties of the HB behavior and the mechanisms underlying phase transitions in minerals and inorganic structures under high-pressure conditions.

In general, a crystal structure (atomic positions, site occupancies, and atomic displacement parameters) can be obtained by X-ray diffraction (XRD) analysis using a spherical model of the atomic electron density (IAM). The IAM, introduced a century ago, does not allow atoms to exchange electron density, which leads to a loss of information about the deeper electronic structure of crystals, charge flow, and the asphericity of electron density. The recent progress in quantum crystallography, combined with significant improvements in the XRD apparatus (brighter X-ray sources, very small beam size, and low-noise, sensitive detectors with superior quantum efficiency), allows for aspherical refinement of electron density distributions against high-resolution X-ray data even at high-pressure conditions.^17,18^ Using the aspherical atom model (AAM) of atomic electron density at extreme conditions is crucial for mineralogy and inorganic chemistry because it provides insights into the electronic parameters of mineral structures beyond just the bonding geometry, including the detection of very subtle phase transitions and their mechanisms, as well as the nature of interatomic interactions. This leads to a superior understanding of the behavior of matter under the prevailing conditions in the Earth’s deep interior. However, there are many obstacles in analyzing electron density distributions refined against X-ray data at high-pressure conditions. The use of DACs in such experiments leads to a reduced resolution, completeness, and quality of the obtained data.^19,20^ To overcome these drawbacks, very bright sources (modern synchrotron facilities with a very short wavelength radiation) and multiple pieces of best-oriented crystals inside DACs are used to ensure high resolution and completeness of the data at extreme conditions. Despite the significant challenges, there have been several successful reports describing changes in the electron density distribution at elevated pressure for pure elements,^21^ of inorganic compounds with the use of maximum entropy methods,^22^ or of organic structures.^20,23^ The most recent works on grossular,^24^ langbeinite,^25^ or hsianghualite^26^ demonstrated the successful determination of electron density distributions for minerals at high-pressure conditions using the multipole refinement method. In this study, we applied the same approach to investigate HB symmetrization in the mineral natrochalcite. Since the XRD method has major limitations in locating hydrogen positions, it is important to compare the obtained AAM with the model refined against single-crystal neutron or electron diffraction data if it is possible to collect them.

Natrochalcite and its potassium analogue kaliochalcite are structurally closely related to other members of the tsumcorite group of minerals.^27^ This copper mineral, characterized by the emerald green color (Figure 1a) and slow solubility in water, forms as a product of weathering processes. Natrochalcite gained importance in recent years as a potential anode material for lithium-ion batteries used in powering consumer electronics and vehicles.^28^ Moreover, natrochalcite-type compounds feature the H_3_O_2_^–^ bihydroxide complex forming the shortest, low-barrier HB reported thus far among the hydrogen-bearing minerals.^29^

**Figure 1:**
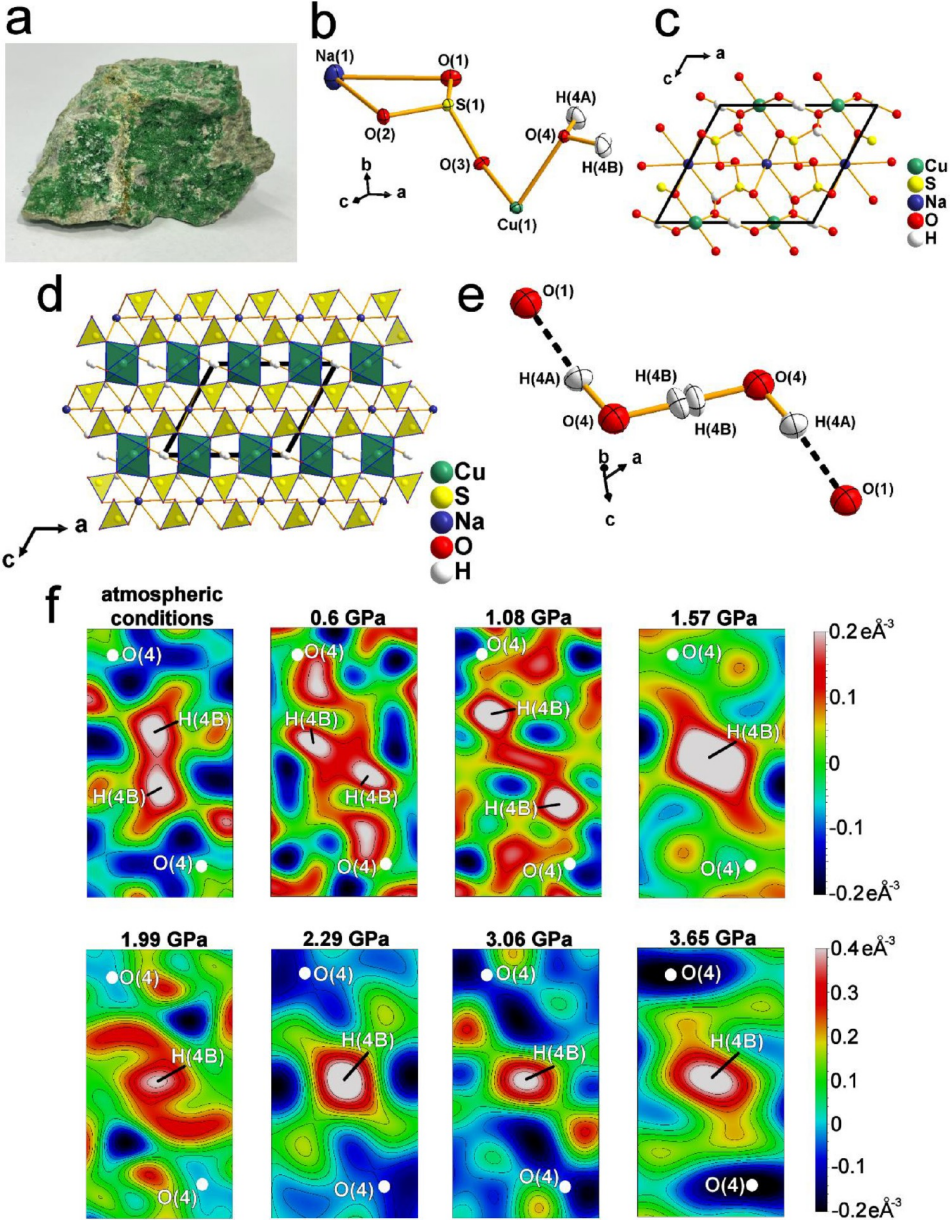
Crystal structure of natrochalcite and residual Fourier electron density maps at various pressure conditions. (a) Sample of natural natrochalcite [NaCu_2_(SO_4_)_2_ ·H_3_O_2_] from Chuquicamata mine, Antofagasta, Chile. Small pieces from this sample were studied in this work. (b) Asymmetric unit of the natrochalcite crystal structure at RT conditions. (c) Atomic arrangement within the unit cell at RT conditions. (d) Projection of the polyhedral model of the natrochalcite structure at RT conditions along the Y-direction. (e) Bihydroxide ion in the structure of natrochalcite at RT conditions forming two HBs: shorter O(4)–H­(4B)···O(4) with disordered hydrogen atoms and longer O(4)–H­(4A)···O(1) H-bond. (f) Residual Fourier electron density maps calculated using the AAM without the hydrogen atom (H4B). The Fourier summation was performed on a grid parallel to the plane of the H_2_O molecule. The scales on the right side of the images represent values for the maximum and minimum peaks of residual density for maps in a particular row (eÅ^–3^).

It is essential to understand the process of HB symmetrization, since the formation of strong symmetric HBs in hydrous minerals enables water transport to the deeper part of Earth’s mantle.^30–32^ Beyond minerals, similar bihydroxide units have been observed in high-pressure organic salts and ice phases, where strong HBs are critical for proton conduction.^33,34^ Moreover, it was theoretically predicted that the HB symmetrization strongly affects the superconductivity of hydrogen sulfide, characterized by the highest critical temperature (*T*_c_) measured so far.^35–38^ In this study, for the first time, we utilized a combination of charge density analysis with single-crystal electron and neutron diffraction experiments to gain insights into the mechanism of HB symmetrization in bihydroxide H_3_O_2_^–^ anion at high pressure.

## 2 Results and Discussion

### 2.1 Crystal Structure of Natrochalcite Mineral

Natrochalcite [NaCu_2_(SO_4_)_2_·H_3_O_2_] crystallizes in the centrosymmetric space group *C*2/*m* and its crystal structure consists of infinite chains of edge-sharing CuO_6_ octahedra extending along the [010] direction, which are responsible for the green color. The chains are connected along the [100] direction by very short HBs (donor–acceptor distance at room temperature = 2.445 Å) formed by the H_3_O_2_^–^ bihydroxide anions (Figure 1d). Single-crystal neutron diffraction and spectroscopic studies at ambient conditions revealed that in the O(4)–H­(4B)···O(4) moiety, the hydrogen atom position is disordered and split into two positions separated by an inversion center, each with a 50% H atom occupancy (Figure 1e).^39,40^ Additionally, the bihydroxide anions also participate in the formation of a second, O(4)–H­(4A)···O(1) HB with a more typical donor–acceptor distance of 2.695 Å. The CuO_6_ octahedra are linked to SO_4_ tetrahedra (yellow color; Figure 1d) by sharing corner oxygens and to sodium atoms coordinated to 8 oxygen ligands (NaO_8_), thus creating a 3D framework (Figure 1b,c).

### 2.2 Observation of HB Symmetrization

The main goal of this study was to establish the mechanism of HB symmetrization beyond atomic positions and site occupancies and determine the electron density features responsible for controlling the O–H···O potential energy function. We performed a multipole refinement^41^ against single-crystal synchrotron XRD data collected at high pressure at ID27 beamline station at the European Synchrotron Radiation Facility (ESRF) (see Section 4). This approach allows us to trace very subtle changes at the electron density level and gives us insights into the detailed mechanism of HB symmetrization. We first refined the AAM of natrochalcite, without the hydrogen atom H­(4B) present, to observe how the positions of residual electron density peaks associated with this particular H atom change at elevated pressure (Figure 1f). These difference maps revealed that at room temperature (RT) and ambient pressure, the hydrogen atom is indeed disordered and split into two positions (phase I). However, at 1.57 GPa, only one positive peak of electron density is present between the oxygen atoms (phase II), indicating that the ordering of the hydrogen atom and HB symmetrization occur at very low pressures, between 1.08 and 1.57 GPa. At ambient pressure, the disordered hydrogen atoms are nearly collinear with respect to the O(4) atom, whereas with increasing pressure (0.6 and 1.08 GPa), the peaks of electron density shift apart, indicating a nonlinear arrangement. Additionally, the disordered H atoms move to an off-center position toward the oxygen atoms, forming a very short O–H covalent bond and a longer H···O HB at 1.08 GPa (Figure 1f). The symmetrization of the HB leads to a significant decrease in the distance between the oxygens O(4), driven by the changing nature of atomic interactions within the bihydroxide ion during the transition, suggesting that the entire process has a discontinuous character (Figure 2c). Despite this, we did not observe discontinuous changes in the unit-cell volume and normalized *a*, *b*, and *c* lattice parameters plotted as a function of pressure (Figure 2a,b). To analyze the obtained volume data, we employed the Vinet equation of state (EOS). The results showed that applied EOS did not accurately fit the volumetric data across the entire pressure range. Fitting the data separately for both phases yielded a much more accurate representation; interestingly, the calculated value of the bulk modulus (*K*_0_) for phase I differs slightly from the one for phase II. The EOS for the data ranging from ambient pressure to 1.08 GPa gives *K*_0_ = 55.24 (±3.84) GPa, while the data from 1.57 to 3.65 GPa yields *K*_0_ = 63.22 (±0.27) GPa. These results indicate that the HB symmetrization process is a second-order transition and the structure with symmetric HB is slightly less compressible. The data were also fit to a Birch–Murnaghan EOS, and the results differed by less than 5% in comparison to results from Vinet EOS. The ordering of hydrogen atom positions appears when the O(4)···O(4) distance is between 2.440 and 2.429 Å, which aligns well with recent work by Meier et al. They found, using high-resolution ^1^H NMR, that a maximum in hydrogen mobility (a precursor to the localization of the hydrogen atom) occurs when the oxygen–oxygen distance d­(O···O) is in a narrow range between 2.44 and 2.45 Å, regardless of the chemical environment of the O–H···O unit. The HB symmetrization also leads to significant discontinuous changes in the geometry of the sulfate moiety (Figure 2d) (see Section 6 in the Supporting Information).

**Figure 2:**
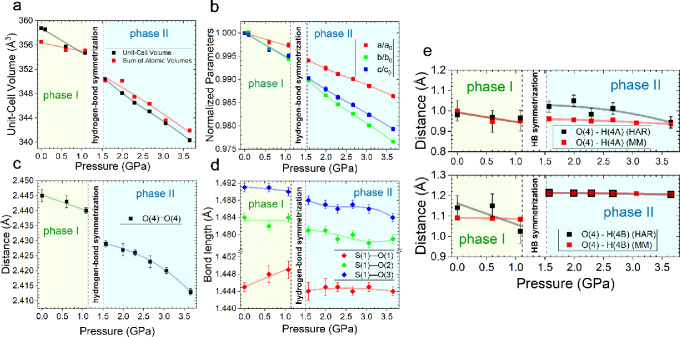
Structural changes for natrochalcite as a function of pressure. (a) Changes of the unit-cell volume as a function of pressure. Black squares represent values of the unit-cell volume from the X-ray synchrotron data collection. Red squares represent the sum of integrated atomic volumes for all atoms in the unit cell at particular pressure conditions. Integrated volumes were calculated using the quantum theory of atoms in molecules (QTAIM) applied to the experimental data. (b) Normalized unit-cell parameters as a function of pressure. (c) Distance between two oxygen atoms O(4) from the O(4)–H­(4B)···O(4) unit as a function of pressure. (d) Changes in the bond length of the sulfate moiety as a function of pressure. (e) Comparison of O–H bond lengths obtained with the Hirshfeld atom model (HAR, black squares) and MM (red squares). For all graphs, the white square represents the pressure region where phase transition driven by HB symmetrization occurs, and error bars represent the standard deviations.

We performed Hirshfeld atom refinement (HAR) against the collected high-pressure X-ray data and compared the results with the outcome of refined multipole models (MMs). At atmospheric conditions, the O–H bond distances obtained with multipolar refinement for both hydrogen atoms are closer to the corresponding neutron values than those obtained with HAR (Table S2). This is a surprising result since the oxygen–hydrogen bond distances from MM are usually a bit too short and less precise than the ones acquired with HAR.^42,43^ At high-pressure conditions, the values of the O–H bond lengths from multipole refinement are in good agreement with those from HAR (Figure 2e). However, using the HAR method only, we were able to observe the shortening of the O(4)–H­(4B) bond and increasing distance between disordered positions of the hydrogen H­(4B) atom prior to HB symmetrization (Table S2, Figure 2e). Additionally, HAR revealed the dependence of the O(4)–H­(4A) bond on the HB symmetrization manifested by the discontinuous elongation of that bond during phase transition (Figure 2e).

### 2.3 Single-Crystal Electron Diffraction Experiment—IAM Dynamical Refinement and Dynamical Kappa Refinement

The recent development of dynamical refinement for electron diffraction (ED) data demonstrates that this method enables the precise and accurate location of hydrogen atoms in single crystals even for inorganic materials.^44,45^ However, there was only one successful attempt to apply a dynamical approach against ED data for a mineral sample.^46^ Additionally, the localization of the disordered hydrogen positions around the inversion center, as it happens in the natrochalcite mineral, is the ultimate challenge even for the dynamical refinement. The previous attempt for a similar situation in cobalt aluminophosphate led to only one broad maximum between oxygen atoms on the difference potential map.^44^ To overcome these obstacles, we collected the ED data for natrochalcite at atmospheric pressure up to the ultrahigh resolution of *d* = 0.40 Å (sinθ_max_/λ = 1.25 Å^–1^), and additionally, we used the dynamical kappa refinement approach to improve the model of the structure obtained with common IAM refinement. A recent work by Suresh et al.^46^ showed that it is possible to apply kappa refinement for the data collected by the ED method and derive a more accurate structural model along with information on the ionization of atoms. In the case of natrochalcite, implementation of the kappa refinement reduced the noise level on the difference potential maps, which made it easier to interpret some very fine details connected to a disordered hydrogen atom from a strong HB (Figure 3a,b). Calculated difference maps in the plane parallel to the bihydroxide ion plane obtained with the kappa model of natrochalcite without the H­(4B) atom revealed two symmetry-dependent electron density maxima around the inversion center between oxygen atoms (Figure 3c,d). This is in excellent agreement with our previous results acquired by modeling the experimental electron density (Figure 1f) and shows that the hydrogen atom H­(4B) is disordered at atmospheric pressure. The maxima belonging to the positions of the disordered H atom from the model of the natrochalcite structure refined against ED data are not colinear with respect to the line joining the O(4) atoms (Figure 3c,d) and look more like that at 0.6 GPa pressure from the MM refined against the X-ray data (Figure 1f). This is probably caused by the low-temperature conditions applied during ED measurement since the sample was kept at around 80 K to maintain vacuum conditions inside the microscope chamber. There are many studies suggesting that pressures between 0.2 and 0.5 GPa can be expected to cause the same change of crystal volume as the temperature decrease from 300 to 100 K.^48–50^ To determine how much the unit-cell volume of natrochalcite decreased at low-temperature conditions, we collected the XRD data at 100 K, since the ED method has limited accuracy in the measurement of the unit-cell parameters.^51^ The unit-cell volumes of natural samples of natrochalcite at 100 K from Chuquicamata mine, Chile (*V* = 355.74 Å^3^, Table S10), and Lavrion Mining District, Greece (*V* = 355.3 Å^3^),^27^ corresponded very well with the unit-cell volume of natrochalcite at 0.6 GPa (*V* = 355.78 Å^3^, Table S7). Moreover, the kappa refinement approach allowed us to freely refine the atomic displacement parameters (ADPs) for hydrogen atoms in natrochalcite (Figure 3e).

**Figure 3:**
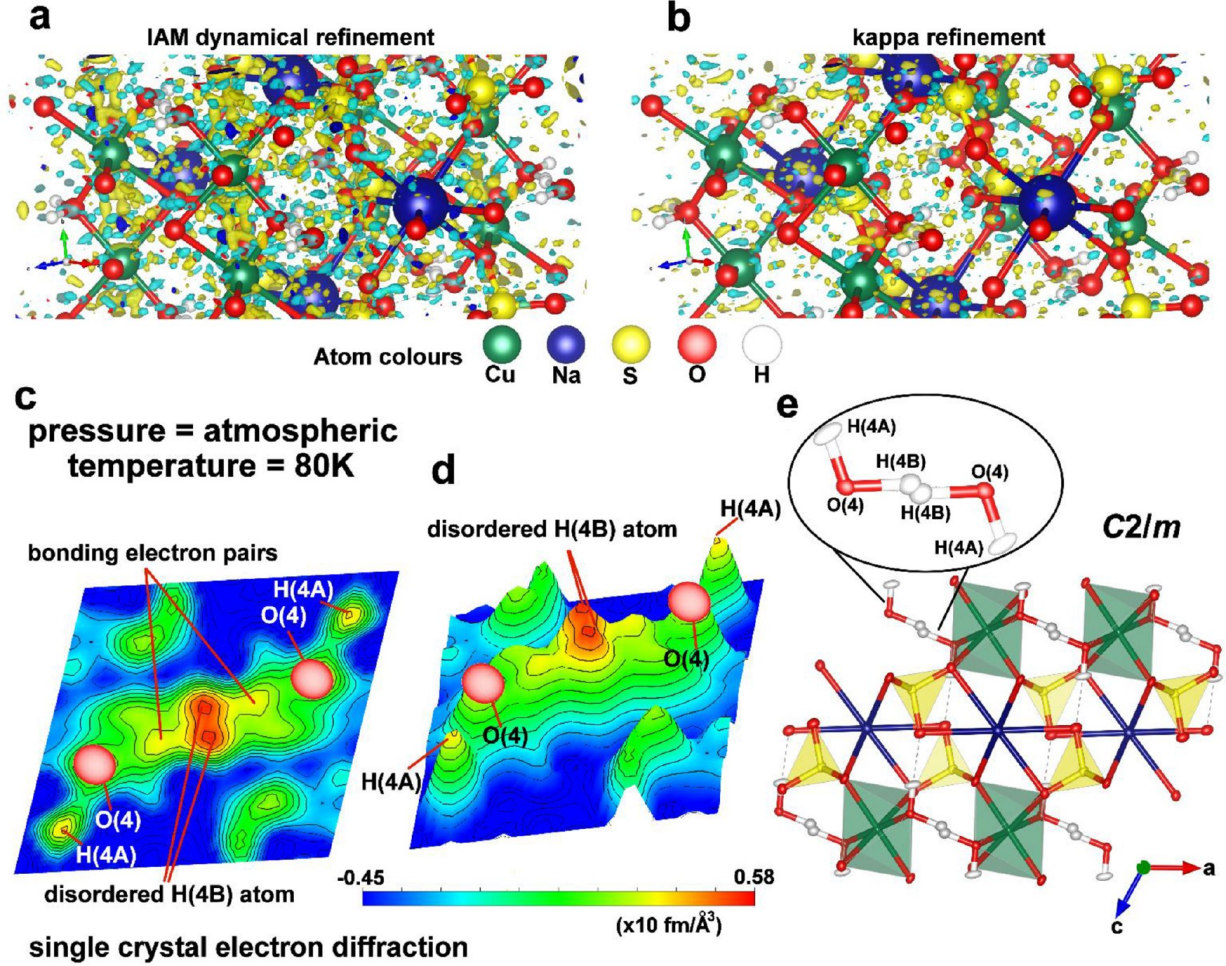
Disorder of hydrogen atom H­(4B) revealed by single-crystal electron diffraction experiment. (a) 3D difference potential map of a section of natrochalcite around the bihydroxide ion after IAM dynamical refinement and (b) after kappa refinement. (c) Difference potential map in the plane parallel to the bihydroxide ion plane calculated using the kappa model without the hydrogen atom H­(4B). (d) Bird’s eye view of the difference map. (e) Structure model of natrochalcite along the *Y*-direction at 80 K obtained with kappa refinement.

### 2.4 Single-Crystal Neutron Diffraction Experiment at High-Pressure Conditions

We performed a single-crystal neutron diffraction study of natrochalcite on a DEMAND instrument of the High-Flux Isotope Reactor (HFIR) at Oak Ridge National Laboratory (ORNL) to confirm the HB symmetrization effect at high-pressure conditions. The single-crystal neutron diffraction method is the most efficient tool to precisely locate the position of hydrogen atoms and properly constrain the geometry of HBs in the crystal structure. The difference Fourier map of the refined model at 1.6 GPa (*P* = 1.66 ± 0.18 GPa as calculated^52^) from neutron data without hydrogen atom H­(4B) revealed only one minimum peak at the middle of the HB (Figure 4a), as in Figure 1f (1.57 GPa pressure). With this result, the neutron diffraction indicated that HB symmetrization already took place at ∼1.6 GPa [the O(4)–O(4) distance at that pressure is 2.430(14) Å according to the model refined against neutron data), and it is in perfect agreement with the results from multipole refinement against X-ray data. Moreover, the minimum peak visible in Figure 4b has a very narrow shape, which suggests the absence of proton tunneling or any dynamical disorder. The H-bond symmetrization does not lead to a symmetry change in the crystal structure of natrochalcite, and the space group remains the same (*C*2/*m*) for a high-pressure phase (Figures 3e and 4c). This is in a very good agreement with a previous study on transition from disordered to ordered HBs under high-pressure conditions, which revealed that symmetrization does not affect crystal symmetry.^10,53^ To the best of the authors’ knowledge, this is the first direct observation of pressure-induced symmetrization of HB using the single-crystal neutron diffraction method since all of the previous studies about the phenomenon are based on powder neutron diffraction.^10,16,53^

**Figure 4:**
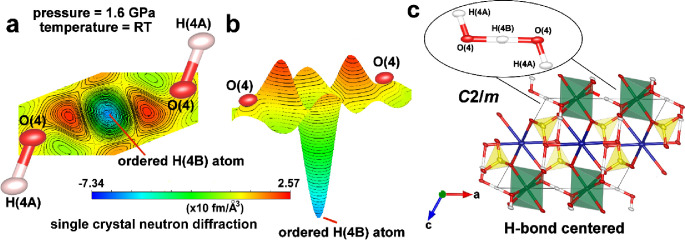
HB symmetrization within the natrochalcite structure at 1.6 GPa revealed by a single-crystal neutron diffraction experiment. (a) Difference Fourier map calculated using the model without the hydrogen atom H­(4B) refined against neutron data at 1.6 GPa. Hydrogen has a negative scattering factor, so the missing H atom is recognized as a negative distribution.^47^ (b) Bird’s eye view of the difference Fourier map. (c) Structural model of natrochalcite along the Y-direction at 1.6 GPa with symmetric HB.

### 2.5 Topological Properties of Electron Density

Calculated topological properties from the experimental electron density at the bond critical points (BCPs) were used to classify HB interactions under high-pressure conditions. In the topological analysis, covalent and ionic interactions are categorized as shared and closed-shell interactions, respectively.^54,55^ The results show that in the case of symmetric HB (1.57–3.65 GPa range), there is a shared interaction between the O(4)–H­(4B) atoms since the values of the Laplacian of the electron density ∇^2^ρ­(**r**_BCP_) are negative (Tables S3 and S5). For most pressure points, this covalent character of symmetric HB is rather moderate, since the Laplacian assumes small negative values. However, for the highest measured pressure (3.65 GPa), the Laplacian at the BCP from the MM is much more negative (closer to the values characteristic for the standard covalent O–H, N–H, or C–H bonds). This suggests the strengthening of symmetric HB with increasing pressure (Table S3). Additionally the large values of the electron density ρ­(**r**_BCP_) (in the range of 0.93–1.49 eÅ^–3^) combined with a negative total energy density *H*(**r**_BCP_) indicate the significant strength of symmetric HB. In weak and moderate HBs, typical values of ρ­(**r**_BCP_) are much lower in the order from 0.05 to 0.23 eÅ^–3^.^56,57^ In the case of longer HBs, ρ­(**r**_BCP_) and ∇^2^ρ­(**r**_BCP_) values revealed closed-shell interactions, typical for classical HB (Tables S4 and S6). Topological properties at HB critical points calculated using models obtained with HAR are in very good agreement with the properties obtained with multipole refinement (Tables S3–S6). For phase II, HAR revealed a similar increase of electron density and decrease of total energy density values at the O4–H­(4B)···O(4) HB critical points, related to significant strengthening of that symmetric bond. A change in the values of Laplacian from positive (phase I) to negative (phase II) shows a transition of the HB from the closed shell to a strong shared interaction between the O(4) oxygen and H­(4B) hydrogen atoms.

### 2.6 Maps of Experimental Electron Density Distributions for the Bihydroxide Anion

The negative Laplacian maps revealed changes in the arrangement of hydrogens in the bihydroxide anion similar to those observed on the residual Fourier density maps (atm conditions − 0.6 GPa range) (Figure 5a). At 1.08 GPa, just before the HB symmetrization, the blue iso-contour starts to appear in the middle of the O(4)···O(4) contact. This shows that the shift of disordered H atoms with increasing pressure to a nonlinear arrangement and off-center positions is driven by the significant outflow of electron density from the center of HB toward oxygen atoms at 1.08 GPa (phase I; Figure 5a). At 1.57 GPa, the red iso-contour appeared at the center point between the O(4) ···O(4) atoms, and it is associated with the localization of electron density at the H atom position at the middle of HB (phase II). This means that the hydrogen atom is fully ordered, and the process of HB symmetrization in the bihydroxide anion is completed at that pressure point. The maps of differences of the negative Laplacian values for hydrogen H­(4B) represent zones where the electron density increases with pressure (red iso - contours) or space of charge depletion at elevated compression (blue iso-contours) when pressure values change from a smaller to a bigger value, as defined at the top of the plots in Figure 5b. We observed significant concentration of electron density off the O(4)···O(4) line when the pressure changes from atmospheric conditions through 0.6 up to 1.08 GPa. However, during pressure elevation from 1.08 to 1.57 GPa, a discontinuous electron density switch occurs and it starts accumulating in the zone poor in electron density (in the middle of the HB) and the previously depleted space becomes electron-density-rich (Figure 5b). Additionally, further increase in pressure leads to an expansion of the charge concentration at the ordered hydrogen position in the perpendicular direction to the HB, especially in the pressure range of 3.06–3.65 GPa.

**Figure 5:**
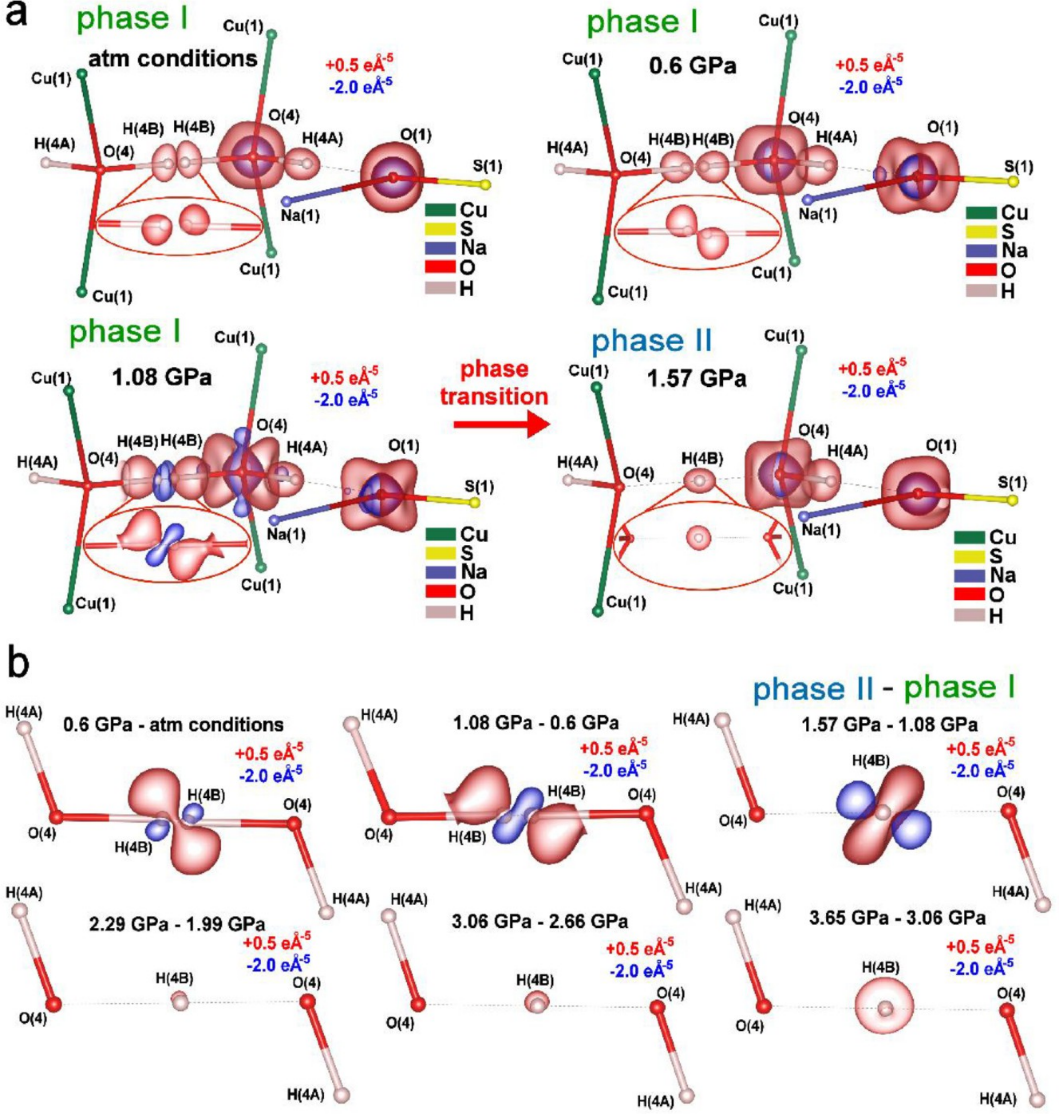
3D maps of negative Laplacian presenting how the electron density redistributes with elevated pressure. (a) 3D maps of the negative Laplacian obtained from the experimental electron density distribution for both HBs formed by the bihydroxide anion. Red contours showing regions of charge concentration, and iso-contours are at +0.5 eÅ^–5^. Blue contours showing regions of charge depletion, and iso-contours are at −2.0 eÅ^–5^. (b) 3D maps of differences in negative Laplacian values for the H­(4B) atom. These maps represent the difference in charge concentration and depletion between pressure points defined at the top of the particular figures. Red contours show regions of charge concentration, and iso-contours are at +0.5 eÅ^–5^. Blue contours showing regions of charge depletion, and iso-contours are at −2.0 eÅ^–5^. The presented levels of iso-contours are the same for all pressure points for better comparability.

3D static deformation density maps show that the charge outflow from the center of HB (phase I) starts already at lower pressure, since we noticed the appearance of a small red iso-contour (charge depletion) on the map at 0.6 GPa (Figure 6). At 1.08 GPa, bonding electron pairs (blue iso-contours at the bonds, charge concentration) are deformed for both covalent bonds, O(4)–H­(4B) and O(4)–H­(4A). This effect is probably connected with the charge transfer and metastability of the structure close to the phase-transition point. After the HB symmetrization, at 1.57 GPa, bonding electron pairs between oxygens O(4) and hydrogen H­(4B) are directed toward the H atom, which indicates the covalent character of the symmetric HB (Figure 6). Further pressure increase leads to an increase in the volume of these electron pairs (2.66 GPa; Figure S11), and finally, at 3.65 GPa, pronounced charge transfer across the symmetric HB O(4)–H­(4B)–O(4) is visible (Figure 6). The calculated theoretical 3D deformation density maps for phase II are in good agreement with the experimental maps (Figure S12). A complete set of more detailed 3D maps of negative Laplacian and static deformation density, represented with lower values for iso-contour levels, is given in the Supporting Information.

**Figure 6:**
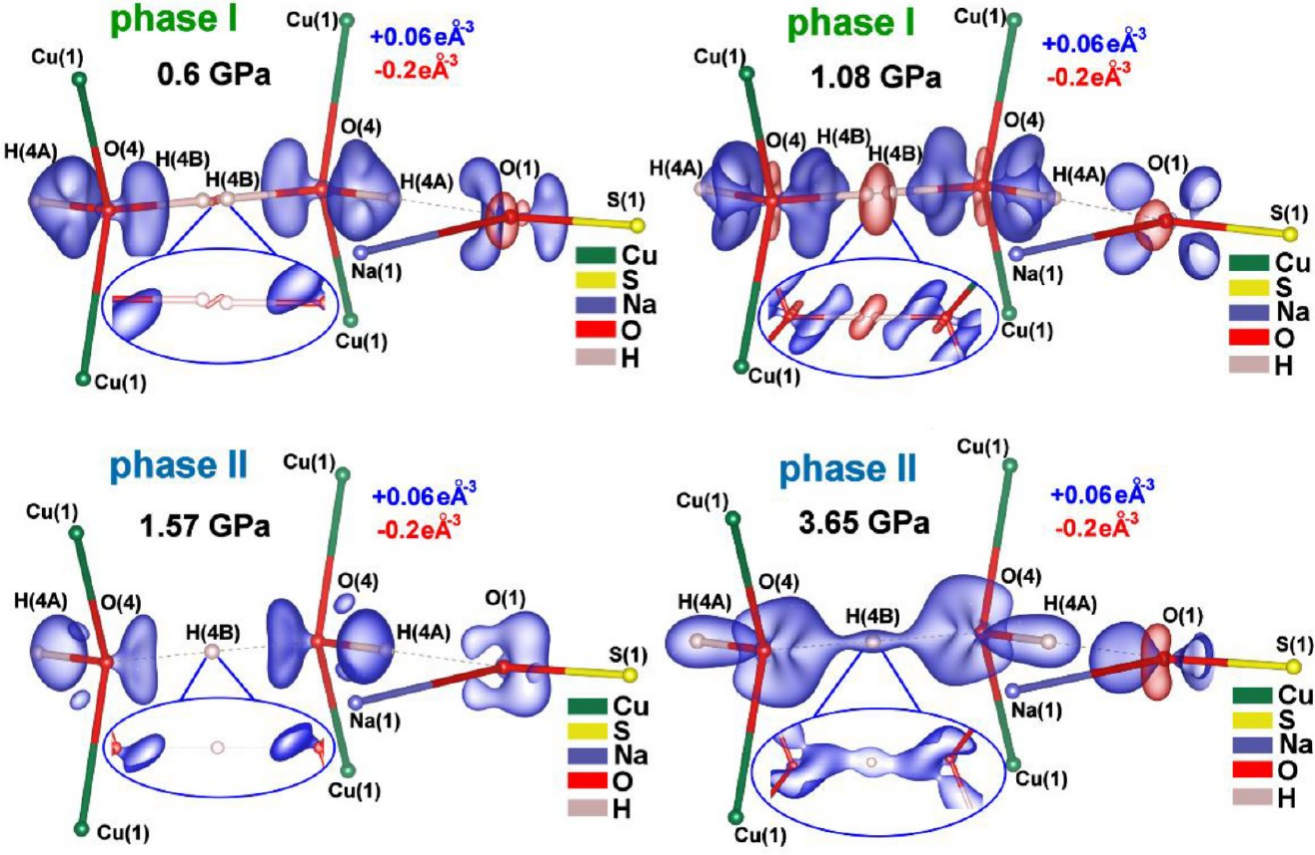
3D maps of static deformation density obtained from the experimental electron density distribution for both HBs formed by the bihydroxide anion. Blue and red colors indicate positive and negative regions of electron density, respectively, and reveal lone and bonding electron pairs. Positive iso-contours are at +0.06 eÅ^–3^ and negative iso-contours are at −0.2 eÅ^–3^. The presented levels of iso-contours are the same for all pressure points for better comparability.

### 2.7 Changes of Atomic Basins as a Function of Pressure

The presented partition of the electron density over atomic basins also reveals charges and volumes for each particular atom in the asymmetric unit (Table S1). The sum of all integrated atomic volumes in the whole unit cell is nearly equal to the measured volumes of the unit cell from XRD experiments for every high-pressure point (red squares, Figure 2a). Since increasing pressure leads to a change in the shape of the atomic basins, a charge flow is observed between atoms in the structure of natrochalcite. The changes of charges and volumes as a function of pressure for particular atoms are represented in Figures S2–S4, which show discontinuous changes for both parameters around the transition point. Furthermore, we noticed that changes in the atomic volumes are inversely proportional to changes in the atomic charges. Our results enable the decomposition of the commonly used EOS into atomic shares and the discussion of properties of atomic EOS. The changes in the shape of atomic volumes as a function of pressure are very complex and dependent on local interactions (see Section 8 in the Supporting Information). Additionally, the values of atomic charges and volumes obtained with theoretical calculations are in very good agreement with the experimental values obtained with multipole refinement (Figures S2–S4).

Before the phase transition (ambient conditions − 1.08 GPa), the wall of the atomic basin of oxygen O(4) from the side of the HB has an irregular shape, which is caused by the disorder of the H atom (Figure 7a). For the second phase, when the hydrogen atom is fully ordered (1.57–3.65 GPa), it leads to the formation of the O(4) atomic basin wall with a more regular shape on the HB side. Since the electron density from disordered hydrogens drifts away toward the O(4) atoms at phase I, the increase of atomic volume for that atom is noticeable before HB symmetrization (Figure S3d). This leads to the expansion of the O(4) atomic basin in the gradient direction (toward bonding edges) at 1.08 GPa (green color, Figure 7a). For the second phase, we observed a change in the shape of the O(4) basin only at 1.57 GPa, right after the phase transition. In this case, the atomic basin expands toward the antigradient edges (green color), in the opposite direction compared to phase I. The ordering of the H atom and change in HB interactions for the second phase lead to the transition from the H­(4B) atomic basin, which has a shape elongated in the direction out of plane to HB, to the atomic basin with its shape elongated in the direction planar to HB (Figure 7b). Moreover, at 1.08 GPa, there is a notable compression of the H­(4B) basin connected with charge depletion from positions associated with the disorder hydrogen before the phase transition. We also observed that for the second phase, the volume of the hydrogen atom H­(4B) increases with elevated pressure (Figure S4b) because of a constant increase in the charge concertation in the H atom position, which is visible on the 3D negative Laplacian maps of HBs (Figure 5b) and the changing shape of the H­(4B) atomic basin (Figure 7b). This effect is responsible for the strengthening of the symmetric HB under high-pressure conditions. A complete set of 3D superimposed atomic basins is given in the Supporting Information.

**Figure 7:**
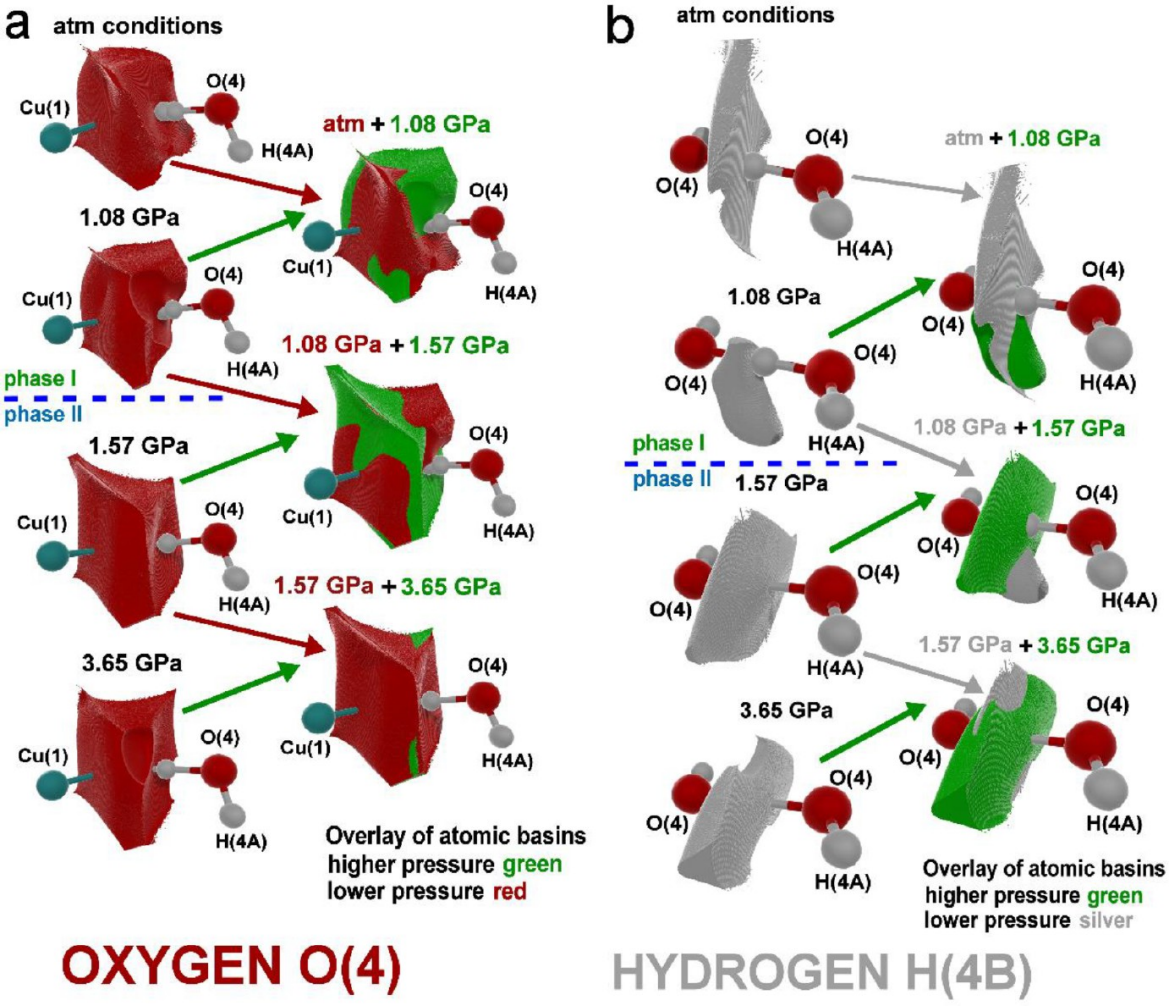
Atomic basins for atoms from the O(4)–H­(4B)···O(4) moiety under high-pressure conditions. (a) Shape changes of the oxygen O(4) atomic basin influenced by high pressure. (b) Shape changes of the hydrogen H­(4B) atomic basin influenced by high pressure. Atomic basins at lower pressures are superimposed on the atomic basins at higher pressures colored in green. Parts where the green color is on the top reveal volume expansion due to increasing pressure, and in the remaining fragments, the electron density of the atom (and hence volume) is compressed.

## 3 Summary and Conclusions

Our single-crystal electron diffraction, neutron diffraction, and XRD experiments revealed that the bihydroxide anion in natrochalcite undergoes HB symmetrization at the lowest pressure (between 1.08 and 1.57 GPa) reported so far for any inorganic compounds. As a comparison, HB symmetrization for FeOOH occurs above 45 GPa,^58^ for δ-AlOOH at 18.1 GPa,^10^ and for D_2_O around 80 GPa.^16^ This shows that natrochalcite is a perfect example to study the HB symmetrization process, because a lower pressure is more experimentally accessible than a pressure of several orders of magnitude. To the best of the authors’ knowledge, this work presents the most detailed study of the HB symmetrization process reported so far, utilizing for the first time single-crystal electron and neutron diffraction methods to investigate this phenomenon.

Previous studies suggest that HB symmetrization is a continuous phase transition^15,16,59,60^ (especially in the case of ice structures), and only one study indicates that it is a discontinuous process and exhibits a clear transition.^61^ Our results show that HB symmetrization is unambiguously a second-order phase transition, and this process leads to a less compressible structure due to the appearance of symmetric HB with an unusually strong covalent character; its formation can have significant influence on the physical properties of mineral structures under conditions in the Earth’s mantle and in high-pressure hydrogenous superconductor systems. HB symmetrization is preceded by continuous changes in the arrangement of disordered hydrogen atoms combined with their counterintuitively off-center movement toward oxygen atoms with elevated pressure (Figures 1f, 5a,b, and 6). This H atoms behavior prior to the symmetrization was predicted before, but it was based only on theoretical calculations.^62–65^ Calculated topological properties at BCPs for HBs revealed that the HB symmetrization is also accompanied by the transition of HB from a closed shell to shared interaction. This change triggers a decrease in the oxygen–oxygen distance (Figure 2c).

The deformation density maps show significant charge transfer across symmetric HB at the highest measured pressure (3.65 GPa; Figure 6). Very high electron density ρ­(**r**) and negative Laplacian ∇^2^ρ values at BCP for symmetric HB at this particular pressure (Tables S3 and S5) indicate its strong covalent character. There are many theoretical predictions that the origin of high-temperature superconductivity for hydrogen sulfide comes from the strong covalency of symmetric HBs,^35,66,67^ which fortifies the stability of the structure. We present for the first time experimentally that charge transfer and strong covalent character are possible for the symmetric HB at high-pressure conditions, and this effect can have a significant impact on the appearance of high-temperature superconductivity. In this study, we successfully refined a full multipolar model for nine structures of natrochalcite under various pressure conditions. Using the AAM of atomic electron density, we were able to detect very subtle changes in the structure and the electron density redistribution, even those connected with hydrogen atoms. Furthermore, the model refined against single-crystal neutron diffraction data at 1.6 GPa supports results from multipolar refinement and confirms that natrochalcite undergoes HB symmetrization. Currently, neutron scattering experiments with large sample space (∼ 10 mm^3^) clamp cells for single-crystal investigations are restricted to a low accessible pressure range of up to ∼ 2 GPa. DACs for single-crystal neutron studies in the higher pressure range have a limited sample space of ∼ 0.1 mm^3^, making it challenging to obtain large-volume data with good signal ratio for studying fine structure displacements in low-symmetry systems. Therefore, not all systems are suitable for neutron diffraction investigations under extreme pressure conditions. In this view, the presented AAM approach is a very good alternative to neutron experiments, especially when pressure is applied. However, the use of synchrotron radiation for high-pressure experiments is the only way to obtain high resolution and high completeness of X-ray data necessary to enable multipole refinement.

## 4 Methods

### 4.1 Single-Crystal XRD Data Collection

The single crystal of natural natrochalcite from the Chuquicamata mine, Antofagasta, Chile, was measured at ambient pressure and temperature to serve as a reference for high-pressure studies. High-resolution single-crystal X-ray diffraction (SCXRD) data up to 0.47 Å were collected using an in-house Rigaku Oxford Diffraction SuperNova four-circle diffractometer equipped with an EOS CCD detector and a molybdenum microsource (Mo *K*α, λ = 0.71 Å). The raw data were processed with CrysAlisPro data reduction program (version 1.171.43.105a). Shape-based absorption correction was applied by using the same software. The intensities of the reflections were corrected for Lorentz and polarization effects. The hydrogen atoms were visible on the difference Fourier maps, and their positions were refined freely by using the IAM approach. The crystal structure was refined by the full-matrix least-squares method using SHELXL incorporated in OLEX2.^70,71^ Sortav program^72^ in WinGX suite^73^ was used to merge symmetry equivalent Bragg reflections and average data sets. Structures were visualized using Diamond 4.^74^

### 4.2 High-Pressure SCXRD Data Collection

High-pressure SCXRD experiments were conducted at the ID27 synchrotron beamline at the European Synchrotron Radiation Facility (ESRF) (λ = 0.22 Å, beam size 2 × 2 μm), equipped with an EIGER2 X CdTe 9 M detector. We loaded three preoriented crystals of natural natrochalcite (from the same sample as for the experiment at ambient conditions) into the membrane-driven DAC (BETSA-type). These crystals were placed into the DAC with slightly different orientations from each other. This multicrystal approach allows for an increase in the reciprocal data coverage measured for the sample at high-pressure conditions. However, significant difference in crystal orientations should be avoided since it leads to improper scaling during merging of the data and high *R*_int_ values for the obtained models. A 4:1 mixture of methanol/ethanol was used as a pressure-transmitting medium. The pressure inside the DAC was measured in situ by the ruby-fluorescence method^75^ with a precision of 0.05 GPa and taking into account the temperature correction.^76^ At each pressure point, the data were collected in step scans of 0.5° upon rotating the DAC from −32° to +32° about the vertical axis (ω-scan). The beam always probed only one of the three crystals, so we were able to collect the diffraction patterns for every crystal separately. The very-high-resolution data up to 0.32 Å were collected at 10 pressure points in the range of 0.1–3.65 GPa. The data sets collected for two pieces of the natrochalcite crystals were processed using CrysalisPro program and merged using Proffit merge program within the CrysalisPro package. We decided to remove from the merging procedure the data collected for the third crystal, despite the better completeness of the merged data, as we observed a significant increase in residual electron density values (+1.5, −2.0 eÅ^–3^) compared to the model refined against merged data collected for only two crystals. The completeness of the data is stable through all of the measured resolution shells at all pressure points, and it is in the range of 65–80% (see Section 12 in the Supporting Information). This strategy for the HP experiment allows us to obtain extremely high completeness for the sample that crystallizes in the monoclinic system. The obtained values of *R*_int_ are very low for all collected pressure points, even after merging the data, and they are similar to those obtained for standard charge density data collections without DAC. The crystal structure at all pressure points was resolved by direct methods^68,69^ and refined by the full-matrix least-squares method using SHELXL incorporated in OLEX2.^70,71^ Sortav program^72^ in WinGX suite^73^ was used to merge symmetry equivalent Bragg reflections and average data sets.

### 4.3 Collecting and Processing ED Data

Single crystals of natural natrochalcite from the same locality as samples used in XRD experiments with no additional preparation were placed on a lacey-carbon grid by using a manual blotting technique under ambient conditions. The grid was clipped and placed inside a Thermofisher 200 kV Glacios FEG Cryo transmission electron microscope (CryoTEM). Inside the microscope chamber, the sample was kept at 80 K and 8 × 10^–6^ Pa before and during the measurement. A high-quality single crystal of the investigated compound was selected for the ED experiment. Diffraction frames were collected with 200 kV electron radiation (λ = 0.0251 Å) using the Ceta-D detector and EPU-ED software with 2 × 2 binning. Data were collected using a phi-scan tilt axis from –60 to 60° with a 0.5° step size and 1s exposure time. The chosen camera length value of 652 mm allowed for data collection at a resolution higher than that of the diffracting limit of the sample. The raw data were processed with CrysAlisPro data reduction program (version 1.171.43.105a). The structure of natrochalcite was refined using JANA 2020 software.^77^ The starting model was taken from the refinement of the X-ray data under atmospheric conditions. The structure was initially refined by using a kinematical approximation. It was then followed by dynamical refinement using Dyngo software integrated into JANA 2020. One scale factor per frame and one thickness parameter for the whole data set were refined in the first refinement cycle with all other parameters fixed. In the next steps, all parameters were refined simultaneously, and in further refinement cycles, atomic displacement parameters and the thickness model (wedge) were introduced to increase the accuracy of the obtained model. JANA 2020 was used to perform kappa refinement, and the Mott–Bethe formula was applied to convert the X-ray structure factors to electron structure factors. The refinement used form factors from STO wave functions implemented in JANA 2020 software. The initial values of *P*_val_ were taken as the number of valence electrons in the neutral state, and all κ values were initially set to unity. The structure obtained from IAM refinement using the theory of dynamical diffraction was taken as a starting model for kappa refinement.

### 4.4 High-Pressure Single-Crystal Neutron Experiment

The single-crystal neutron diffraction experiment of synthetic natrochalcite (see Supporting Information) was performed at the Dimensional Extreme Magnetic Neutron Diffractometer (DEMAND) instrument of the HFIR at the Oak Ridge National Laboratory (ORNL) with a high pixelated large-area detector. Neutron wavelength λ = 1.003 Å generated with the multilayer-[110]-wafer silicon monochromator was used for the experiment. A piston-cylinder type of pressure cell (clamp cell) was employed for applying pressure. Fluorinert 70:77 in a 1:1 ratio was used as the pressure medium. A NaCl crystal was included in the cell, together with the natrochalcite sample for pressure calibration. Neutron diffraction data of the natrochalcite sample were collected at a nominal pressure of 1.6 GPa, which is *P* = 1.66 ± 0.18 GPa calculated with the NaCl EOS function.^52^ The data processing was performed using Mantid 6.12.0 program.^78^ The crystal structure was refined by the full-matrix least-squares method using SHELXL incorporated in OLEX2.^70,71^

### 4.5 Experimental Electron Density Distribution

The IAM models were used as starting points, and a multipole refinement was performed on F^2^. For phase I (atm–1.08 GPa pressure range), the starting models had a disordered H­(4B) atom, and for phase II (1.57–3.65 GPa pressure range), hydrogen atom H­(4B) was fully ordered. The input file for the multipole refinement of structure models from phase I included a fixed 50% occupancy for the hydrogen H­(4B) atom. The refinement strategy for the full multipolar model was as follows for every pressure point. First, atomic coordinates (*xyz*) and atomic displacement parameters (*Uij*) were refined together with the scale factor (*s*) using the high-order XRD data of sinθ/λ > 0.7 Å^–1^ for the non-hydrogen atoms. The terms *xyz* and *Uij* were fixed after high-order refinement. In the next step, the atomic displacement parameters (ADPs) of the H atoms were determined by combining a rigid-body analysis of the O atom ADPs using SHADE software.^79^ Then, the procedure was performed in a stepwise manner, adding new parameters in the following order: scale factor (*s*) and valence populations (*M*), dipoles (*D*), quadrupoles (*Q*), octupoles (*O*), and hexadecapoles (*H*) for Cu, S, and O atoms. The Na atom was treated as a spherical Na^+^ cation, with all multipole parameters fixed at 0. Next, the positions (*xyz*) of hydrogen atoms were refined alone, and then, the bond-directed dipole and quadrupole (*Q*_0_) for H atoms were added to the refinement. The starting values for hydrogen atom positions were taken from the neutron experiment at atmospheric conditions.^39^ We did not use any geometric constraints on the distances between O and H during refinement. In the last step, *s* was refined with spherical κ parameters for Cu, S, O, and H atoms. The resolution employed in the refinement was sin θ/λ = 1.1 Å^–1^. XD2020 ^80^ program was used for the multipolar modeling. More information about multipole refinement can be found in the Supporting Information (Section 1). All 3D negative Laplacian and deformation density maps were generated using the XDPROP module of the XD2020 suite and visualized using VESTA software.^81^ Topological parameters at BCPs between atoms were determined from the experimental electron density by using the XDPROP module in XD2020. Partitioning of the electron density into atomic basins was performed using the XDPROP module within XD2020 and Multiwfn^82^ programs. Atomic Basins were visualized using VMD software.^83^

### 4.6 Hirshfeld Atom Refinement

HAR was performed using the DiSCaMB library^84^ integrated with Olex2 starting from the IAM geometry. Aspherical atomic scattering factors were obtained as a result of quantum mechanical calculations carried out with Orca^85,86^ using the B3LYP functional and the Def2-SVP basis set. To simulate the influence of the crystal environment, which is particularly important for network crystals, two clusters of atoms, consisting of 92 and 76 atoms, were used for wave function calculations. The atoms used for calculations of atomic form factors were located at the centers of the clusters. During HAR, hydrogen atoms were refined isotropically, and the only constraints imposed on hydrogen positions were the crystal symmetry-related ones. Topological parameters at BCPs between atoms were determined using the Multiwfn program. The HAR for the structure measured at 3.06 GPa pressure was not successful.

## Supplementary Material



## Notes

Determination and analysis of quantitative electron density distributions (EDDs) in crystals from XRD experiment and DFT calculations; syntheses of natrochalcite single crystals; integrated atomic charges and volumes for natrochalcite; geometry of HBs in natrochalcite as a function of pressure obtained with multipolar refinement, HAR, and single-crystal neutron diffraction; topological properties at critical points of all HBs in natrochalcite as a function of pressure obtained with multipolar refinement and HAR; impact of HB symmetrization on the crystal structure of natrochalcite; a complete set of 3D maps of negative Laplacian and static deformation density for natrochalcite; a complete set of integrated atomic basins for natrochalcite; comparison of experimental results for AAMs at various pressure conditions by the analysis of residual electron density; IAM and MM refinement against X-ray data—experimental tables; experimental tables for single-crystal data collected using electron and neutron diffraction methods; completeness of the collected data at high-pressure conditions (PDF)

## Abbreviations

HB: hydrogen bond
DAC: diamond anvil cell
atm: atmospheric conditions
BCP: bond critical point
MM: multipole model
AAM: aspherical atom model
HAR: Hirshfeld atom refinemen;
ED: electron diffraction
ESRF: European Synchrotron Radiation Facility
HFIR: High-Flux Isotope Reactor
ORNL: Oak Ridge National Laboratory
VSCC: valence shell charge concentration
CSCD: core–shell charge depletion
EOS: equation of state
SCXRD: single-crystal X-ray diffraction
