# Supplementary material for: Symmetrization of Strong Hydrogen Bond under High Pressure in Bihydroxide-Ion-Containing NaCu2(SO4)2·H3O2 Revealed by Experimental Charge Density, Single-Crystal Electron Diffraction, and Neutron Diffraction Studies
Source: J Am Chem Soc. 2025 Jul 17;147(30):26830–43. doi: 10.1021/jacs.5c08310 (PMC12314919; doi:10.1021/jacs.5c08310)
Supplement: Supplementary file 1 [file ja5c08310_si_001.pdf]

Supplementary information for

Symmetrization of strong hydrogen bond under high pressure in bihydroxide-ion-containing $NaCu_2(SO_4)_2{\cdot}H_3O_2$ revealed by experimental charge density, single crystal electron diffraction and neutron diffraction studies

Piotr Rejnhardt[a*], Roman Gajda[a], Magdalena Woińska[a], Jan Parafiniuk[b], Gerald Giester[c], Ronald Miletich[c], Yan Wu[d], Tomasz Poręba[e], Mohamed Mezouar[e], Szymon Sutuła[f], Tomasz Góral[f], Przemysław Dera[g], Krzysztof Woźniak[a*]

[a]Department of Chemistry, University of Warsaw, Pasteura 1, Warszawa, 02-089, Poland.
[b]Department of Geochemistry, Mineralogy and Petrology, Faculty of Geology, University of Warsaw, Żwirki i Wigury 93, 02-089, Warszawa, Poland.
[c]Department of Mineralogy and Crystallography, University of Vienna, Josef-Holaubek-Platz 2, Vienna A-1090, Austria.
[d]Neutron Scattering Division, Oak Ridge National Laboratory, Oak Ridge, Tennessee 37831, USA.
[e]ID27 High Pressure Beamline, European Synchrotron Radiation Facility (ESRF), 71 avenue des Martyrs, 38000 Grenoble, France.
[f]Centre of New Technologies, University of Warsaw, S. Banacha 2c, 02-097, Warszawa, Poland
[g]Hawai'i Institute of Geophysics and Planetology, University of Hawai'i at Manoa, 1680 East-West Road, Honolulu, HI 96822, USA.
*Correspondence authors. Email: p.rejnhardt@chem.uw.edu.pl, kwozniak@chem.uw.edu.pl

Contents

Section 1: Determinations and analysis of quantitative electron density distributions (EDDs) in crystals from X-ray diffraction experiment and DFT calculations 2
Topological analysis of electron density 3
Density functional theory (DFT) calculations 4
Section 2: Syntheses of natrochalcite single crystals 5
Section 3: Integrated atomic charges and volumes for natrochalcite 6
Section 4: Geometry of hydrogen bonds in natrochalcite as a function of pressure obtained with multipolar refinement, Hirshfeld atom refinement and single crystal neutron diffraction 13
Section 5: Topological properties at critical points of all hydrogen bonds in natrochalcite as a function of pressure obtained with multipolar refinement and Hirshfeld atom refinement 14
Section 6: Impact of HB symmetrization on the crystal structure of natrochalcite 16
Section 7: A complete set of 3D maps of negative Laplacian and static deformation density for natrochalcite 19
Section 8: A complete set of integrated atomic basins for natrochalcite 25
Section 9: Comparison of experimental results for aspherical atom models at various pressure conditions by the analysis of residual electron density 31
Section 10: Independent Atom Model and Multipole Model refinement against X-ray data - experimental tables 44
Section 11: Experimental tables for single crystal data collected using electron and neutron diffraction methods 48
Section 12: Completeness of the collected data at high pressure conditions 50
Section 13: References 58

Section 1: Determinations and analysis of quantitative electron density distributions (EDDs) in crystals from X-ray diffraction experiment and DFT calculations

One of the crucial conditions to get accurate EDD is sufficiently high resolution of obtained X-ray diffraction data. By 'sufficient high resolution' we mean measurements for which $(\sin\theta/\lambda)_{max}$ value is no less than 1.1 $Å^{-1}$.[1] This condition must be fulfilled since multipolar refinement of electron density brings up to 32 additional parameters per atom so it requires many more unique observations to obtain proper data/parameter ratio.[2] For comparison Independent Atom Model (IAM) refines only 9 parameters per atom – atomic positions and atomic displacement parameters (ADPs). Moreover, the low diffraction-angle data range contributes only to the valence electron density and high-resolution reflections are strongly associated with nuclei positions and particularly with ADPs. It shows, that using low resolution data leads to significant loss of important structural information, especially in the context of hydrogen atoms positions. The other conditions are sufficiently completeness of collected data, accurate and precise measurement of the intensities of the reflections or accurate correction of systematic effects such as absorption by the sample, extinction or absorption by the apparatus itself (diamond anvil cell in the case of high pressure experiment).[2,3] Since the contribution of valence electrons to the total value of scattered reflections intensity is around a few percent it is pivotal to conduct very accurate measurements based on the aforementioned conditions.

The most common, aspherical quantitative model of experimental charge density, is based on a finite spherical harmonic expansion of the electronic part of the charge distribution around each atomic center. Such an atomic expansion is called a pseudoatom and the molecular electron distribution at any point in a crystal is the sum of all the pseudoatomic densities. In the most commonly used formalism of Hansen and Coppens[4,5] the pseudoatom electron density is defined by:

$$\rho(\mathbf{r}) = \rho_c(r) + P_V\kappa^3\rho_V(\kappa r) + \sum_{i=0}^{l_{max}} \kappa'^3 R_l(\kappa' r) \sum_{m=0}^{l} P_{lm\pm} d_{lm\pm}(\Theta,\varphi)$$

where $\rho_c(r)$ and $\rho_V(r)$ are spherical core and valence densities, respectively. The third term contains the sum of the angular functions $d_{lm\pm}(\Theta,\varphi)$ to take into account aspherical deformations. The angular functions $d_{lm\pm}(\Theta,\varphi)$ are real spherical harmonic functions. The coefficients P_V and $P_{lm\pm}$ are populations for the valence and deformation density multipoles, respectively. The κ and κ' are scaling parameters introduced to make valence and deformation densities expand or contract. In the Hansen-Coppens formalism the P_V, $P_{lm\pm}$, κ and $\kappa'$are refineable parameters together with the atomic coordinates and thermal coefficients. Least-squares refinements are performed against the measured intensities F^2(hkl) of reflections obtained by single crystal X-ray diffraction. Starting atomic coordinates and anisotropic displacement parameters are taken from the ordinary spherical refinement stage and freely refined. The O-H bond distances and ADPs of H-atoms are usually taken from neutron diffraction experiment.

In multipole refinement we used a core and spherical-valence scattering factors derived from Clementi and Roetti wavefunctions.[6] A single-ζ Slater type radial function multiplied by density-normalized spherical harmonics are used for describing the valence deformation terms. No extinction correction was necessary since we used very small pieces of crystals and very short synchrotron wavelength (λ = 0.22 Å) Site symmetry restrictions were applied to following non-H atoms for both high pressure phases: the symmetry $\overline{1}$ for Cu(1) atom, the mirror symmetry *m* for S(1), O(1), O(2) and O(4) atoms and the $2/m$ symmetry for Na(1) atom. For atom O(3) no site symmetry restrictions were applied. In the case of hydrogen atoms H(4A) and H(4B) from the phase I the mirror symmetry m were applied for both atoms. For the hydrogen atoms from the phase II the symmetry restriction m were applied for H(4A) atom and symmetry restriction $2/m$ for H(4B) atom. No chemical constrains were applied for the multipole refinement. The multipole expansion was truncated at the hexadecapole level (l_{max} = 4) for Cu, S and O atoms. The following multipoles were released in the refinement for non-H atoms for both high pressure phases: M1, Q0, Q1+, Q1-,Q2+, Q2-, H0, H1+, H1-, H2+, H2-, H3+, H3-, H4+ and H4- for Cu(1) atom, M1, D1+, D1-, Q0, Q2+, Q2-, O1+, O1-, O3+, O3-, H0, H2+, H2-, H4+ and H4- for S(1), O(1), O(2) and O(4) atoms and all multipoles were released for O(3) atom The used ground-state electronic configuration of Cu corresponds to [Ar]$4s^1 3d^{10}$ because it has given the most physically reasonable multipole populations and deformation density for copper atom. The Na atom was treated as a spherical Na^+ cation with all multipole parameters fixed at 0. The residual electrons from sodium atom were distributed over the all O atoms in the asymmetric unit equally to satisfy unit-cell electroneutrality. The position of hydrogen atoms along with bond directed dipole and quadrupole were refined and the starting values for hydrogen atoms positions were taken from the neutron experiment at ambient conditions.[7] The anisotropic displacement parameters (ADPs) of the H atoms were determined combining a rigid-body analysis of the O atom ADPs using the SHADE software separately for every measured pressure data. The H-atoms ADPs were fixed during the whole multipole refinement. For both hydrogen atoms from phase I the following multipoles were released: M1, D1+, D1-, H0. For the phase II the M1, D1+, D1- and H0 multipoles were released for H(4A) atom and the M1 and H0 multipoles were released for H(4B) atom. The radial fit of the valence density was optimized by refinement of the expansion-contraction parameters κ and κ′ parameters were kept fixed at the data base values for all atoms. The high pressure X-ray data used in the multipole refinement were truncated at (sin θ)/λ = 1.1 $Å^{-1}$ resolution for all pressure points since the data above that resolution had lower completeness (ca. 35 – 40%).

Topological analysis of electron density

Different methods of electron density partitioning can be used to define the properties of the studied systems, when aspherical atom model for structure is established. These include such methods as the stockholder[8] pseudoatom partitioning, or - the most popular - Atoms-In-Molecules theory[9] (AIM) proposed by R. Bader. AIM theory[10,11] offers a self-consistent way of partitioning any molecular or crystalline system into its atomic basins, deduced from the first principles of Quantum Mechanics and Schwinger's principle of stationary action.[12] In AIM theory, atomic basins are 3D objects of irregular shape defined by the gradient lines of electron density which start from the local maxima of electron density (atom, ion) and go to infinity except those

ones which terminate at the closest neighboring atoms. Such families of gradient lines are separated by interatomic hypersurfaces of zero flux of gradient of the electron density. These zero-flux surfaces define the shape of atomic basins. Integrating properties over atomic basins is one of the cornerstones of AIM theory because it yields worthwhile information such as the integrated charges, the volumes of atoms/ions, their energies, and electronic populations as well as higher multiple moments,[11] polarizabilities,[13] etc.

Density functional theory (DFT) calculations

We have also optimized the structure of natrochalcite by DFT calculations using *CRYSTAL17* software[14,15]. Calculated on the basis of this optimization, dynamical structure factors ($\tilde{F}_{\mathrm{hkl}}$)[16] have allowed us to obtain theoretical electron density distributions, which can be used as a benchmark for experimental results. Multipole model refinements on the basis of dynamical structure factors were conducted in the same way as refinements on the basis of experimental data. The only differences are as follows: theoretical refinements were of F (not F^2) and datasets were 100% complete. We were not able to optimize the structure model of natrochalcite for phase I (atm – 1.08 GPa pressure range) since *CRYSTAL17* software cannot perform calculations for model with disordered hydrogen atoms.

During the optimization, we used the *B3LYP*[17,18] exchange–correlation function corrected for dispersion by Grimme's *D3*[19] correction in conjunction with the *pob-TZVP-rev2* basis sets[20] for S, Na, O and H atoms and different one ("extended_ruiz_2003") for Cu atom[21]. Calculations were conducted for six pressure points between 1.57 GPa and 3.65 GPa. Optimization of the atomic position was allowed but the cell volume was fixed. Convergence criterion on the root mean square of the gradient (TOLDEG = 0.00085), as well as on the displacement (TOLDEX = 0.0009), was also employed. The calculation grid had 75 radial points and maximum number of 974 angular points in the regions relevant for chemical bonding (XLGRID). The truncation criteria for bi-electronic integrals were adopted (TOLINTEG = 7 7 7 9 30). A Pack-Monkhorst/Gilat shrinking factor was also used (8 – shrinking factor in reciprocal space, 16 – shrinking factor for the Gilat net). The convergence accelerator DIIS (Direct Inversion of the Iterative Subspace convergence accelerator) was also used for mixing Fock/KS matrix derivatives.

Section 2: Syntheses of natrochalcite single crystals

Crystals of $NaCu_2(SO_4)_2{\cdot}H_3O_2$ were grown of a size (i.e. ca. 10 mm^3) and of a quality as required for the neutron diffraction experiment by low-hydrothermal synthesis in a Teflon-lined autoclave and run times of 2 to 3 months. Approximately 0.5 g copper oxide rods (Kupferoxid Drahtform, Art. 2767 Fa. E. Merck, Darmstadt, FRG) were heated in a closed "Teflon"- lined steel vessel of ~ 6 cm^3 volume together with ~0.25 g of the sodium sulphate, very little water and some few droplets of the pertinent acid to 180 —240°C for several days and then slowly cooled.

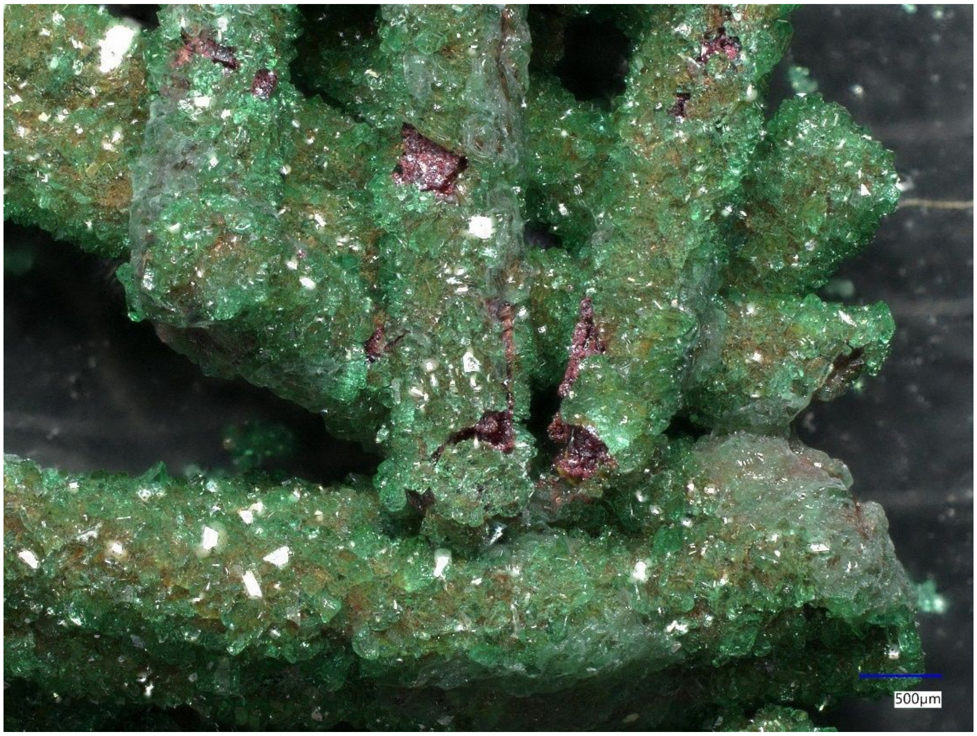

Fig. S1 Sample of synthetic single crystals of natrochalcite [$NaCu_2(SO_4)_2{\cdot}H_3O_2$] studied using neutron diffraction experiment.

Section 3: Integrated atomic charges and volumes for natrochalcite

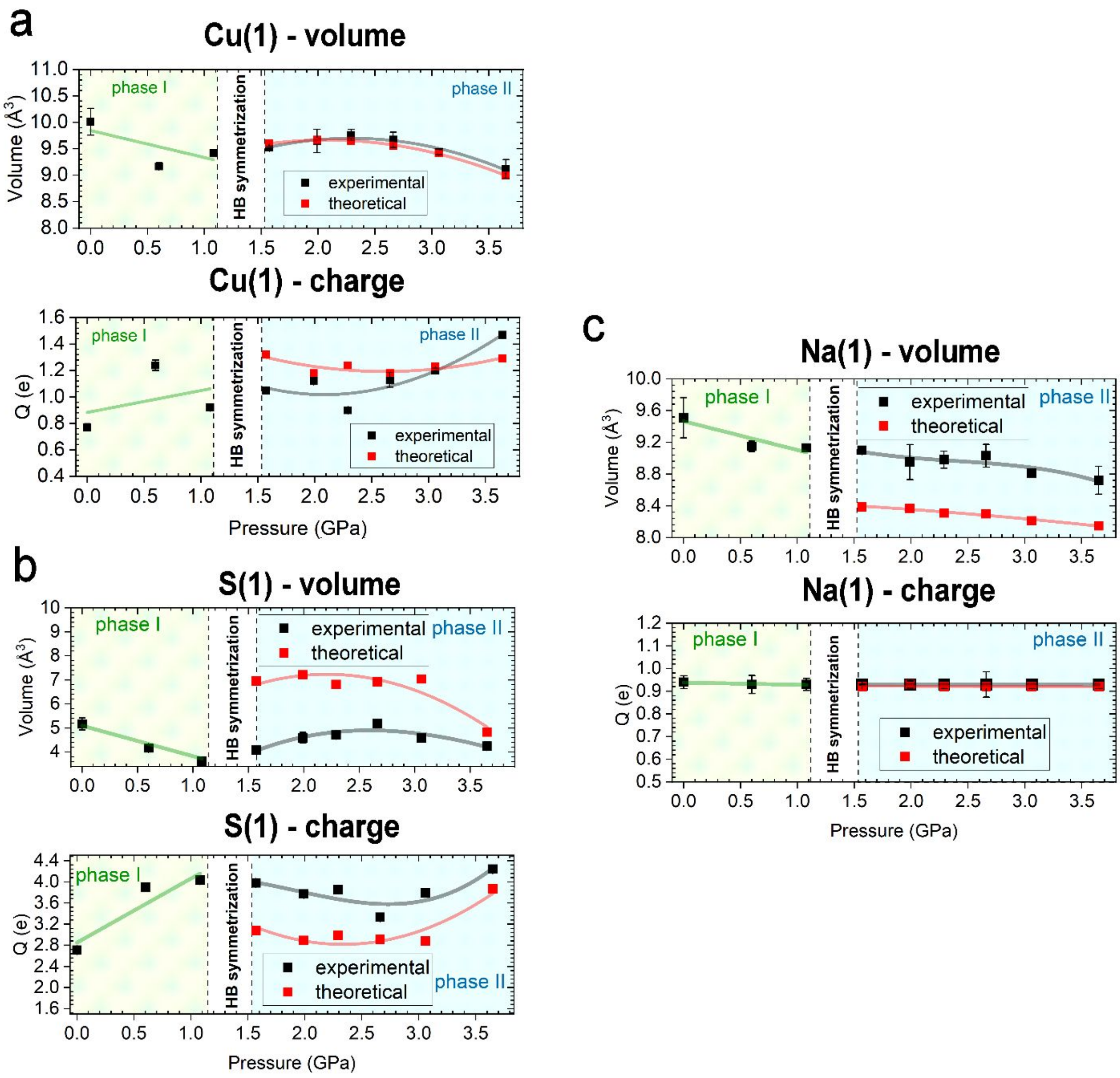

Fig. S2 The values of the net atomic charges and volumes as a function of pressure obtained after integration over atomic basins. Black squares represent the values obtained with experimental data and red squares represent the data obtained with theoretical data. (a) Volumes and charges for copper atom Cu(1) at various pressure conditions. (b) Volumes and charges for sulfur atom S(1) at various pressure conditions. (c) Volumes and charges for sodium atom Na(1) at various pressure conditions. For all graphs the white square represents the pressure region where phase transition driven by hydrogen-bond symmetrization occurs and error bars represent the standard deviations.

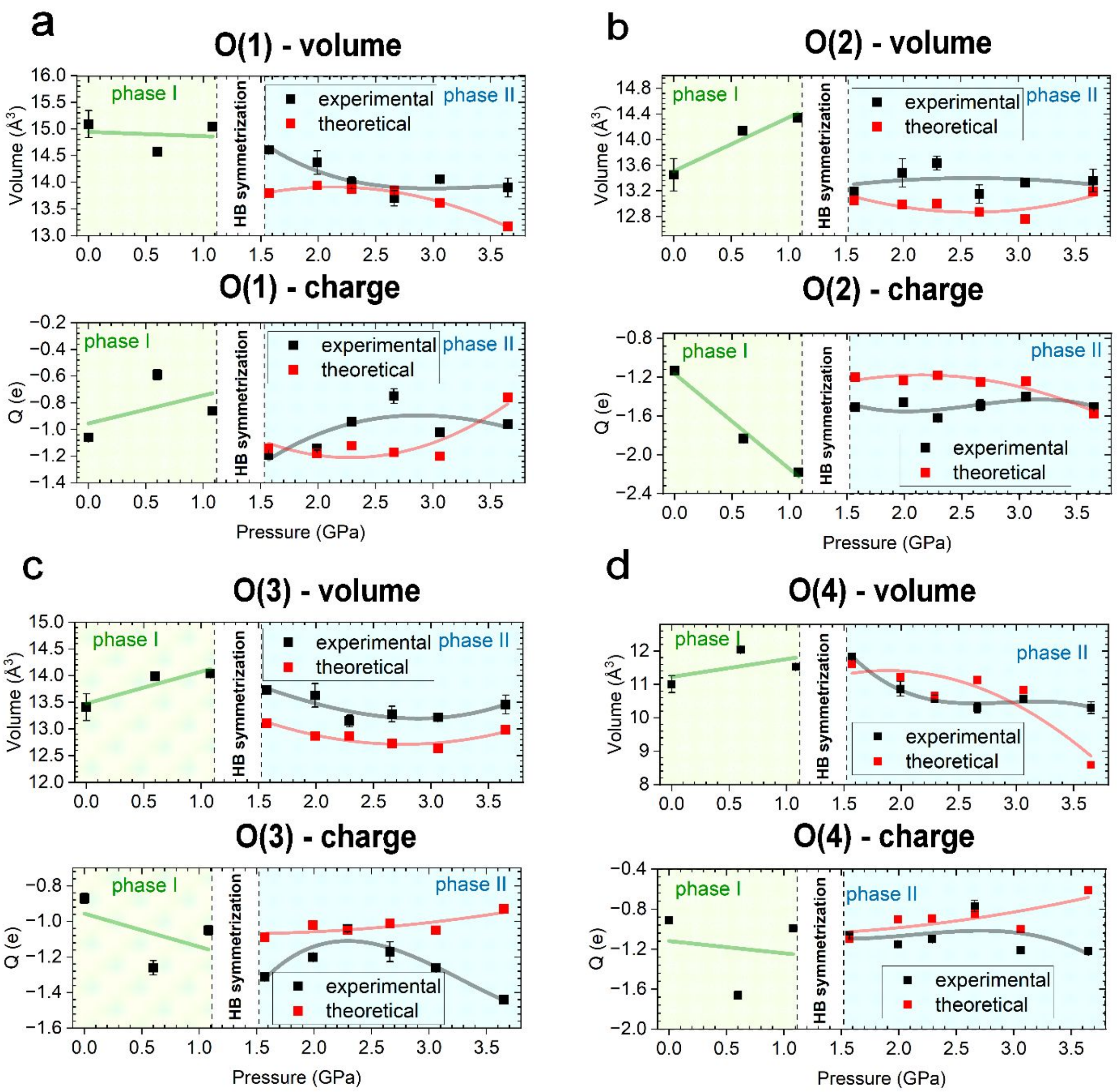

Fig. S3. The values of the net atomic charges and volumes as a function of pressure obtained after integration over atomic basins. Black squares represent the values obtained with experimental data and red squares represent the data obtained with theoretical data. (a) Volumes and charges for oxygen atom O(1) at various pressure conditions. (b) Volumes and charges for oxygen atom O(2) at various pressure conditions. (c) Volumes and charges for oxygen atom O(3) at various pressure conditions. (d) Volumes and charges for oxygen atom O(4) at various pressure conditions. For all graphs the white square represents the pressure region where phase transition driven by hydrogen-bond symmetrization occurs and error bars represent the standard deviations.

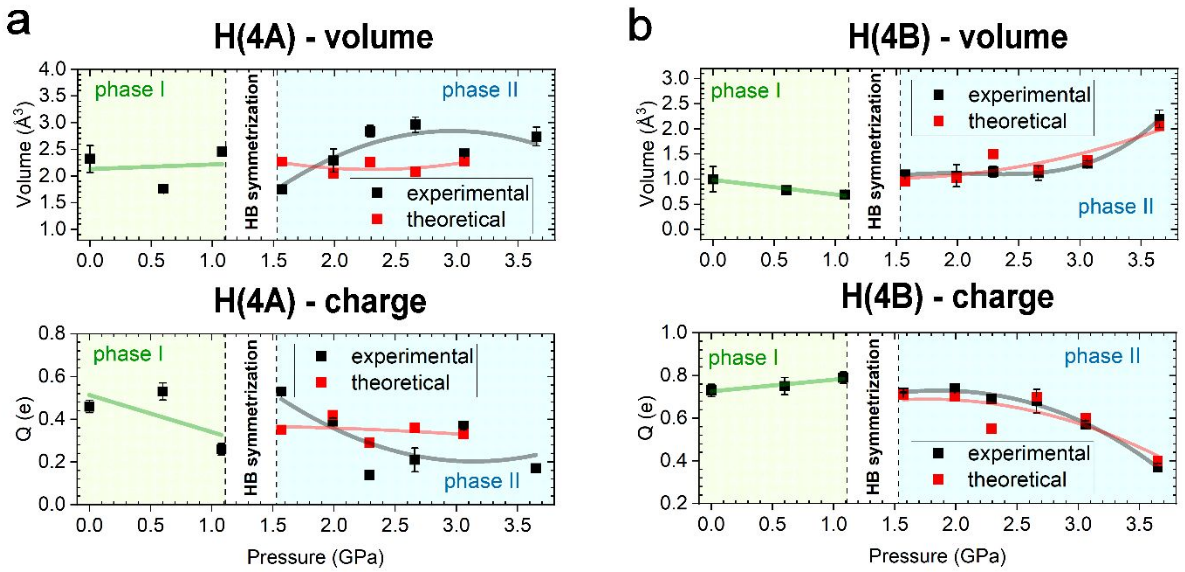

Fig. S4 The values of the net atomic charges and volumes as a function of pressure obtained after integration over atomic basins. Black squares represent the values obtained with experimental data and red squares represent the data obtained with theoretical data. (a) Volumes and charges for hydrogen atom H(4A) at various pressure conditions. (b) Volumes and charges for hydrogen atom H(4B) at various pressure conditions. For all graphs the white square represents the pressure region where phase transition driven by hydrogen-bond symmetrization occurs and error bars represent the standard deviations.

Table S1 Experimental (MM) and theoretical (theory) integrated charges and volumes of Bader atomic basins.

Pressure (GPa)	atom	q (e) MM	q (e) theory	V (Å^3) MM	V (Å^3) theory	Lagrangian (au) MM	Lagrangian (au) theory
	Cu(1)	0.77	------------	10.01	------------	$2.55x10^{-3}$	---------------------------
	S(1)	2.71	------------	5.17	------------	$-1.27x10^{-3}$	---------------------------
	Na(1)	0.94	------------	9.51	------------	$-5.19x10^{-5}$	---------------------------
	O(1)	-1.06	------------	15.09	------------	$1.27x10^{-3}$	---------------------------
**ambient**	O(2)	-1.13	------------	13.45	------------	$4.62x10^{-6}$	---------------------------
	O(3)	-0.87	------------	13.41	------------	$-7.51x10^{-4}$	---------------------------
	O(4)	-0.91	------------	11.01	------------	$1.65x10^{-4}$	---------------------------
	H(4A)	0.46	------------	2.32	------------	$7.39x10^{-4}$	---------------------------
	H(4B)	0.73	------------	1.00	------------	$-3.44x10^{-4}$	---------------------------
	Cu(1)	1.24	------------	9.17	------------	$1.73x10^{-3}$	---------------------------
	S(1)	3.90	------------	4.17	------------	$-8.20x10^{-3}$	---------------------------
	Na(1)	0.93	------------	9.15	------------	$1.56x10^{-4}$	---------------------------
	O(1)	-0.59	------------	14.57	------------	$-2.90x10^{-3}$	---------------------------
**0.6 GPa**	O(2)	-1.83	------------	14.14	------------	$-5.15x10^{-4}$	---------------------------
	O(3)	-1.26	------------	13.99	------------	$-1.68x10^{-3}$	---------------------------
	O(4)	-1.66	------------	12.04	------------	$1.03x10^{-2}$	---------------------------
	H(4A)	0.53	------------	1.76	------------	$8.92x10^{-4}$	---------------------------
	H(4B)	0.75	------------	0.78	------------	$-2.19x10^{-3}$	---------------------------
	Cu(1)	0.92	------------	9.42	------------	$2.10x10^{-4}$	---------------------------
	S(1)	4.03	------------	3.63	------------	$-3.06x10^{-2}$	---------------------------
**1.08 GPa**	Na(1)	0.93	------------	9.13	------------	$1.64x10^{-4}$	---------------------------
	O(1)	-0.86	------------	15.04	------------	$2.73x10^{-4}$	---------------------------
	O(2)	-2.18	------------	14.34	------------	$-6.41x10^{-4}$	---------------------------
	O(3)	-1.05	------------	13.72	------------	$-4.14x10^{-3}$	---------------------------

	O(4)	-0.99	------------	11.53	------------	$1.42x10^{-2}$	---------------------------
	H(4A)	0.26	------------	2.46	------------	$-8.69x10^{-4}$	---------------------------
	H(4B)	0.79	------------	0.69	------------	$2.94x10^{-3}$	---------------------------
**1.57 GPa**	Cu(1)	1.05	1.32	9.52	9.60	$-1.47x10^{-3}$	$-1.27x10^{-2}$
	S(1)	3.98	3.08	4.08	6.95	$-2.34x10^{-3}$	$-2.04x10^{-2}$
	Na(1)	0.93	0.92	9.10	8.39	$-3.13x10^{-5}$	$5.54x10^{-4}$
	O(1)	-1.19	-1.14	14.61	13.80	$-1.72x10^{-3}$	$-2.94x10^{-4}$
	O(2)	-1.51	-1.20	13.19	13.05	$-4.21x10^{-5}$	$-4.77x10^{-4}$
	O(3)	-1.31	-1.09	13.73	13.11	$2.78x10^{-4}$	$7.70x10^{-4}$
	O(4)	-1.06	-1.10	11.83	11.62	$2.94x10^{-3}$	$-2.58x10^{-3}$
	H(4A)	0.53	0.35	1.75	2.27	$2.30x10^{-6}$	$9.01x10^{-4}$
	H(4B)	0.72	0.71	1.09	0.96	$-7.83x10^{-4}$	$3.67x10^{-3}$
**1.99 GPa**	Cu(1)	1.12	1.18	9.65	9.67	$-7.19x10^{-3}$	$-1.23x10^{-2}$
	S(1)	3.77	2.89	4.59	7.21	$-2.22x10^{-2}$	$-1.90x10^{-2}$
	Na(1)	0.93	0.93	8.95	8.37	$-6.88x10^{-5}$	$5.58x10^{-4}$
	O(1)	-1.14	-1.18	14.37	13.94	$1.00x10^{-3}$	$-9.11x10^{-4}$
	O(2)	-1.46	-1.23	13.48	12.99	$4.76x10^{-5}$	$1.02x10^{-4}$
	O(3)	-1.20	-1.02	13.63	12.87	$-9.89x10^{-4}$	$7.99x10^{-4}$
	O(4)	-1.15	-0.9	10.87	11.23	$6.91x10^{-4}$	$-2.65x10^{-3}$
	H(4A)	0.39	0.42	2.29	2.05	$-1.52x10^{-4}$	$1.32x10^{-4}$
	H(4B)	0.74	0.70	1.07	1.03	$-3.45x10^{-3}$	$3.45x10^{-3}$
**2.29 GPa**	Cu(1)	0.90	1.24	9.76	9.65	$-4.58x10^{-3}$	$-1.49x10^{-2}$
	S(1)	3.85	2.99	4.71	6.81	$-3.02x10^{-3}$	$-1.17x10^{-2}$
	Na(1)	0.93	0.92	8.98	8.31	$-1.17x10^{-4}$	$5.52x10^{-4}$
	O(1)	-0.94	-1.12	13.99	13.87	$-1.43x10^{-3}$	$1.94x10^{-4}$
	O(2)	-1.62	-1.18	13.63	13.00	$-1.18x10^{-3}$	$-2.42x10^{-4}$

	O(3)	-1.04	-1.05	13.15	12.87	-9.56x10^{-4}	3.43x10^{-4}
	O(4)	-1.10	-0.89	10.57	10.67	-1.73x10^{-3}	8.40x10^{-4}
	H(4A)	0.14	0.29	2.84	2.26	-4.55x10^{-4}	-1.29x10^{-3}
	H(4B)	0.69	0.55	1.15	1.50	-9.73x10^{-5}	2.57x10^{-5}
**2.66 GPa**	Cu(1)	1.13	1.18	9.67	9.56	-2.79x10^{-3}	-9.12x10^{-3}
	S(1)	3.33	2.91	5.18	6.92	-2.62x10^{-2}	-1.42x10^{-2}
	Na(1)	0.93	0.92	9.03	8.30	-1.43x10^{-4}	5.21x10^{-4}
	O(1)	-0.75	-1.17	13.7	13.85	5.25x10^{-4}	-1.05x10^{-3}
	O(2)	-1.49	-1.25	13.15	12.87	2.10x10^{-4}	5.80x10^{-4}
	O(3)	-1.17	-1.01	13.28	12.73	-1.19x10^{-3}	2.75x10^{-4}
	O(4)	-0.77	-0.85	10.3	11.14	-6.58x10^{-4}	-1.98x10^{-3}
	H(4A)	0.21	0.36	2.96	2.08	-6.98x10^{-4}	-2.09x10^{-3}
	H(4B)	0.68	0.70	1.12	1.19	-1.14x10^{-4}	1.52x10^{-3}
**3.06 GPa**	Cu(1)	1.20	1.23	9.44	9.42	3.18x10^{-3}	-1.10x10^{-2}
	S(1)	3.79	2.88	4.58	7.04	-3.08x10^{-2}	-1.38x10^{-2}
	Na(1)	0.93	0.92	8.81	8.21	-1.18x10^{-4}	4.14x10^{-4}
	O(1)	-1.02	-1.20	14.06	13.61	1.50x10^{-3}	-1.11x10^{-3}
	O(2)	-1.40	-1.24	13.33	12.76	-3.53x10^{-4}	2.55x10^{-4}
	O(3)	-1.26	-1.05	13.22	12.64	1.21x10^{-3}	1.12x10^{-3}
	O(4)	-1.21	-1.00	10.57	10.84	1.46x10^{-3}	-2.70x10^{-4}
	H(4A)	0.37	0.33	2.42	2.27	2.85x10^{-4}	2.68x10^{-3}
	H(4B)	0.57	0.60	1.31	1.38	1.54x10^{-3}	2.43x10^{-3}
**3.65 GPa**	Cu(1)	1.47	1.29	9.12	8.99	-2.16x10^{-3}	-8.73x10^{-3}
	S(1)	4.24	3.87	3.68	4.83	-1.69x10^{-2}	2.55x10^{-2}
	Na(1)	0.93	0.92	8.72	8.15	-1.20x10^{-5}	5.64x10^{-4}
	O(1)	-0.96	-0.76	13.9	13.17	-7.02x10^{-4}	7.96x10^{-4}

O(2)	-1.51	-1.58	13.36	13.19	-5.72×10^{-4}	8.96×10^{-4}
O(3)	-1.44	-0.93	13.46	12.99	-2.49×10^{-4}	4.13×10^{-5}
O(4)	-1.22	-0.61	10.30	8.59	-1.54×10^{-3}	5.95×10^{-2}
H(4A)	0.17	-0.71*	2.74	4.61	-3.41×10^{-4}	-2.58×10^{-2}
H(4B)	0.37	0.40	2.20	2.06	-6.81×10^{-4}	1.95×10^{-2}

*At 3.65 GPa the theoretical calculated value for atomic charge of hydrogen H(4A) atom was negative which is physically unrealistic.

As we know, the increasing pressure leads to the decreasing of the unit cell volume. However the graphs of integrated atomic charges and volumes show, that different basins do not change their volume proportionally to the decrease of the whole unit cell (Fig. S2, S3, S4). For the phase I, oxygen basins slightly expanded their volumes, but the copper, sulfur and sodium basins have shrunk with elevated pressure. Especially the volume of sulfur atom decreased significantly and compensates changes in oxygen basins volumes (Fig. S2b). In the case of phase II, volume changes are more complex. In this phase, oxygen basins volumes behave in the opposite way than in the phase I and mostly all of them have shrunk when pressure increased (Fig. S3). The sulfur atomic basin is the smallest one among non-hydrogen atoms since it is completely surrounded from each side by oxygen atoms. Additionally, the volume of sulfur atom changes proportionally to changes in volumes of oxygen basins with increasing pressure. We also observed, that in the phase II the volume of hydrogen H(4B) atomic basin increased above 3 GPa pressure (Fig. S4b) and it is connected with observed increased in charge concentration with pressure, which is visible on the Laplacian maps (Fig. 5). Since we treated sodium atom as a spherical Na^+ cation, the charge of this cation is stable in the whole pressure range despite the constant decrease in the volume of Na^+ atomic basin with increased pressure (Fig. S2c).

The theoretical values of integrated atomic charges and volumes obtained with *Crystal17* software are in a good agreement with experimental values obtained with multipole refinement. It is worth to mention, that we were not able to perform theoretical calculations using *Crystal17* software for the phase I since it is not possible to do that for structure with disordered positions of atoms. Moreover, theoretical calculations for the structure measured at the 3.65 GPa pressure was partially successful since the obtained charge for H(4B) atom has negative value which is physically unrealistic. The biggest differences are observed for sulfur atom which has less positive charge and in consequence bigger atomic volume in comparison to experimental results (Fig. S1b). Both experimental and theoretical values revealed that oxygen O(4) has the least negative charge among all the oxygen atoms (Fig. S2d). This is the only oxygen involved in the formation of covalent bonds with hydrogen atoms so small negative charge is possibly caused by sharing electron density with H-atoms. Additionally theoretical calculations confirmed that atomic volume decreased with elevated pressure for the O(4) atom from phase II which is connected with strengthening effect of symmetric hydrogen bond."

Section 4: Geometry of hydrogen bonds in natrochalcite as a function of pressure obtained with multipolar refinement, Hirshfeld atom refinement and single crystal neutron diffraction

Table S2 Geometry parameters for hydrogen bonds O(4)-H(4A)···O(1) and O(4)-H(4B)···O(4) at various pressure conditions obtained with multipolar refinement (MM – red), Hirshfeld atom refinement (HAR - green) and single crystal neutron diffraction (blue).

Pressure [GPa]	O(4)-H(4A) [Å]	O(4)-H(4B) [Å]	H(4A)···O(1) [Å]	H(4B)···O(4) [Å]	H(4B)···H(4B) [Å]	O(4)···O(4) [Å]
atm neutron	0.983(2)	1.089(12)	1.712(2)	1.353(11)	0.265(13)	2.443(1)
atm HAR	1.000(50)	1.141(59)	1.706(54)	1.313(50)	0.281(14)	2.444(1)
atm MM	0.982(1)	1.091(1)	1.719(1)	1.354(1)	0.263(1)	2.445(1)
0.6 HAR	0.958(54)	1.159(59)	1.738(47)	1.302(53)	0.355(19)	2.439(1)
0.6 MM	0.969(1)	1.089(1)	1.712(2)	1.354(1)	0.267(1)	2.443(2)
1.08 HAR	0.952(51)	1.025(59)	1.723(48)	1.414(97)	0.391(14)	2.439(1)
1.08 MM	0.966(1)	1.085(1)	1.708(1)	1.355(1)	0.270(1)	2.440(1)
1.57 HAR	1.021(28)	1.214(1)	1.630(28)	1.214(1)	------------------	2.429(1)
1.57 MM	0.961(1)	1.214(1)	1.693(1)	1.214(1)	------------------	2.428(1)
1.6 neutron	0.945(24)	1.215(11)	1.697(24)	1.215(11)	------------------	2.430(1)
1.99 HAR	1.050(28)	1.213(1)	1.594(27)	1.213(1)	------------------	2.426(1)
1.99 MM	0.956(1)	1.213(1)	1.689(1)	1.213(1)	------------------	2.427(1)
2.29 HAR	0.984(27)	1.212(1)	1.649(28)	1.212(1)	------------------	2.424(1)
2.29 MM	0.950(1)	1.213(1)	1.682(1)	1.213(1)	------------------	2.427(1)
2.66 HAR	1.012(28)	1.210(1)	1.617(27)	1.210(1)	------------------	2.420(1)
2.66 MM	0.956(1)	1.211(1)	1.674(1)	1.211(1)	------------------	2.422(2)
3.06 MM	0.942(1)	1.210(1)	1.676(1)	1.210(1)	------------------	2.420(2)
3.65 HAR	0.944(28)	1.206(1)	1.660(27)	1.206(1)	------------------	2.413(1)
3.65 MM	0.936(1)	1.207(1)	1.669(1)	1.207(1)	------------------	2.413(1)

Section 5: Topological properties at critical points of all hydrogen bonds in natrochalcite as a function of pressure obtained with multipolar refinement and Hirshfeld atom refinement

Table S3 Geometry parameters**,** electron density, $\rho(r_{BCP})$ [eÅ^{-3}], Laplacian $\nabla^2\rho(r_{BCP})$ [eÅ^{-5}] and total energy density $H(r_{BCP})$ [hÅ^{-3}], at the (3, -1) BCPs for hydrogen bond O(4)-H(4B)···O(4) at various pressure conditions obtained with multipolar refinement.

Pressure	Interaction	D···A [Å]	D-H [Å]	H···A [Å]	DHA [°]	R_{ij} [Å]	$\rho(r_{BCP})$ [eÅ^{-3}]	$\nabla^2\rho(r_{BCP})$ [eÅ^{-5}]	$H(r_{BCP})$ [hÅ^{-3}]
neutron	O4–H(4B)···O4	2.443(1)	1.089(12)	1.353(11)	179.4(1)	----------	----------	----------	----------
ambient	O4–H(4B)···O4	2.445(2)	1.091(1)	1.354(1)	179.49(1)	----------	----------	----------	----------
0.6 GPa	O4–H(4B)···O4	2.443(2)	1.089(1)	1.354(1)	178.59(1)	----------	----------	----------	----------
1.08 GPa	O4–H(4B)–O4	2.440(1)	1.085(1)	1.355(1)	179.79(1)	----------	----------	----------	----------
1.57 GPa	O4–H(4B)–O4	2.429(1)	1.214(1)	1.214(1)	180.00	1.2161	0.93(2)	-3.14(3)	-0.73(2)
1.99 GPa	O4–H(4B)–O4	2.427(2)	1.213(1)	1.213(1)	180.00	1.2133	0.97(2)	-3.67(3)	-0.79(2)
2.29 GPa	O4–H(4B)–O4	2.426(1)	1.213(1)	1.213(1)	180.00	1.2138	0.99(2)	-4.30(1)	-0.81(3)
2.66 GPa	O4–H(4B)–O4	2.423(2)	1.211(1)	1.212(1)	180.00	1.2134	1.03(2)	-2.08(3)	-0.84(2)
3.06 GPa	O4–H(4B)–O4	2.420(1)	1.210(1)	1.210(1)	180.00	1.2104	1.25(2)	-1.87(4)	-1.16(2)
3.65 GPa	O4–H(4B)–O4	2.413(1)	1.207(1)	1.207(1)	180.00	1.2071	1.49(2)	-9.69(5)	-1.72(3)

Table S4 Geometry parameters**,** electron density, $\rho(r_{BCP})$ [eÅ^{-3}], Laplacian $\nabla^2\rho(r_{BCP})$ [eÅ^{-5}] and total energy density $H(r_{BCP})$ [hÅ^{-3}], at the (3, -1) BCPs for hydrogen bond O(4)-H(4A)···O(1) at various pressure conditions obtained with multipolar refinement.

Pressure	Interaction	D···A [Å]	D-H [Å]	H···A [Å]	DHA [°]	R_{ij} [Å]	$\rho(r_{BCP})$ [eÅ^{-3}]	$\nabla^2\rho(r_{BCP})$ [eÅ^{-5}]	$H(r_{BCP})$ [hÅ^{-3}]
neutron	O4–H(4A)···O1	2.695(1)	0.983(2)	1.712(2)	179.4(1)	----------	----------	----------	----------
ambient	O4–H(4A)···O1	2.697(2)	0.982(1)	1.719(1)	173.89(7)	1.7553	0.39(1)	2.36(1)	0.01(1)
0.6 GPa	O4–H(4A)···O1	2.677(2)	0.969(1)	1.712(2)	173.71(8)	1.9408	0.11(4)	3.67(3)	0.01(1)
1.08 GPa	O4–H(4A)···O1	2.671(1)	0.966(1)	1.708(1)	174.28(4)	1.8909	0.17(2)	4.10(2)	0.01(1)
1.57 GPa	O4–H(4A)···O1	2.654(2)	0.961(1)	1.693(1)	178.27(1)	1.8164	0.13(1)	4.37(1)	0.04(1)
1.99 GPa	O4–H(4A)···O1	2.642(2)	0.956(1)	1.689(2)	174.28(6)	1.7133	0.43(2)	2.90(2)	-0.13(1)
2.29 GPa	O4–H(4A)···O1	2.632(1)	0.950(1)	1.682(1)	178.61(4)	1.6827	0.44(3)	3.28(4)	-0.13(1)
2.66 GPa	O4–H(4A)···O1	2.629(2)	0.956(1)	1.674(2)	176.84(6)	1.697	0.50(2)	4.91(3)	-0.14(1)
3.06 GPa	O4–H(4A)···O1	2.618(2)	0.942(1)	1.676(2)	179.17(1)	1.6782	0.50(4)	1.62(6)	-0.16(1)
3.65 GPa	O4–H(4A)···O1	2.605(1)	0.936(1)	1.669(1)	178.72(5)	1.6734	0.50(2)	4.32(2)	-0.14(2)

Table S5 Geometry parameters, electron density, $\rho(r_{BCP})$ [eÅ^{-3}], Laplacian $\nabla^2\rho(r_{BCP})$ [eÅ^{-5}] and total energy density $H(r_{BCP})$ [hÅ^{-3}], at the (3, -1) BCPs for hydrogen bond O(4)-H(4B)···O(4) at various pressure conditions obtained with HAR.

Pressure	Interaction	D···A [Å]	D-H [Å]	H···A [Å]	DHA [°]	$\rho(r_{BCP})$ [eÅ^{-3}]	$\nabla^2\rho(r_{BCP})$ [eÅ^{-5}]	$H(r_{BCP})$ [hÅ^{-3}]
neutron	O4–H(4B)···O4	2.4430(1)	1.089(12)	1.353(11)	179.4(1)	----------	----------	----------
ambient	O4–H(4B)···O4	2.4440(12)	1.141(59)	1.4300(9)	172(12)	0.62	3.39	-0.93
0.6 GPa	O4–H(4B)···O4	2.4406(13)	1.159(59)	1.8100(7)	179(10)	0.59	3.83	-0.80
1.08 GPa	O4–H(4B)–O4	2.4389(12)	1.025(59)	1.4200(6)	176 (8)	0.63	3.17	-1.01
1.57 GPa	O4–H(4B)–O4	2.4285(10)	1.214(1)	1.2143(5)	180.	1.08	-5.68	-3.39
1.99 GPa	O4–H(4B)–O4	2.4270(2)	1.213(1)	1.2130(6)	180.	1.09	-5.71	-3.41
2.29 GPa	O4–H(4B)–O4	2.4328(10)	1.212(1)	1.2119(5)	180.	1.08	-5.71	-3.40
2.66 GPa	O4–H(4B)–O4	2.4201(10)	1.210(1)	1.2100(5)	180.	1.10	-5.79	-3.46
3.06 GPa	O4–H(4B)–O4	----------	----------	----------	----------	----------	----------	----------
3.65 GPa	O4–H(4B)–O4	2.4125(10)	1.206(1)	1.2063(5)	180.	1.10	-5.87	-3.49

Table S6 Geometry parameters, electron density, $\rho(r_{BCP})$ [eÅ^{-3}], Laplacian $\nabla^2\rho(r_{BCP})$ [eÅ^{-5}] and total energy density $H(r_{BCP})$ [hÅ^{-3}], at the (3, -1) BCPs for hydrogen bond O(4)-H(4A)···O(1) at various pressure conditions obtained with HAR.

Pressure	Interaction	D···A [Å]	D-H [Å]	H···A [Å]	DHA [°]	$\rho(r_{BCP})$ [eÅ^{-3}]	$\nabla^2\rho(r_{BCP})$ [eÅ^{-5}]	$H(r_{BCP})$ [hÅ^{-3}]
neutron	O4–H(4A)···O1	2.695(1)	0.983(2)	1.712(2)	179.4(1)	----------	----------	----------
ambient	O4–H(4A)···O1	2.6973(10)	1.000(50)	1.72(4)	169(4)	0.25	3.50	0.08
0.6 GPa	O4–H(4A)···O1	2.6781(11)	0.958(54)	2.07(5)	178(5)	0.26	3.64	0.08
1.08 GPa	O4–H(4A)···O1	2.6708(11)	0.952(51)	1.71(4)	172(4)	0.26	3.67	0.09
1.57 GPa	O4–H(4A)···O1	2.6507(9)	1.021(28)	1.63(3)	178 (3)	0.31	3.95	0.01
1.99 GPa	O4–H(4A)···O1	2.6413(6)	1.050(28)	1.63(3)	175(3)	0.32	3.99	-0.01
2.29 GPa	O4–H(4A)···O1	2.6323(9)	0.984(27)	1.66(2)	178 (2)	0.29	4.00	0.05
2.66 GPa	O4–H(4A)···O1	2.6256(10)	1.012(28)	1.63(3)	175(3)	0.31	4.07	0.02
3.06 GPa	O4–H(4A)···O1	----------	----------	----------	----------	----------	----------	----------
3.65 GPa	O4–H(4A)···O1	2.6034(10)	0.944(28)	1.67(3)	178 (3)	0.28	4.10	0.08

Section 6: Impact of HB symmetrization on the crystal structure of natrochalcite

The HB symmetrization not only triggered modifications in the geometry of bihydroxide anion in the structure of natrochalcite. There are also significant changes in the bond lengths for the sulfate molecule, especially for S(1)-O(1) bond (Fig. 2d, red diamonds), which is the closest one to the H_2O molecule. Since the oxygen atom O(4) is also a donor of the longer hydrogen bond O(4)-H(4A)···O(1), we observed the valence shell deformations for O(1) at 1.08 GPa (Fig 5a), which are triggered by the HB symmetrization and constant outflow of electron density from O(1) atom towards the S(1)-O(1) bond with increasing pressure (Fig. S6). These changes lead to the significant charge concentration connected with bonding electron pairs of S(1)-O(1) bond which is most visible on the static deformation density maps for the sulfate moiety (starting from 1.57 GPa, Fig. S6) and to the decrease of this bond length after the phase transition (Fig. 2d). The change in the shape of oxygen O(1) atomic basin is also associated with the change in the geometry of sulfate molecule (Fig. S15a). As electron density from O(1) drifts away towards the S(1)-O(1) bond after the phase transition we noticed significant compression of O(1) basin at 1.57 GPa (Fig.S15a). and volume decrease (Fig. S3a). The shape changes for hydrogen H(4A) atomic basin are not as spectacular as for H(4B), however we notice constant expansion of H(4A) basin with elevated pressure after HB symmetrization (Fig S15b). Interestingly aforementioned expansion of H(4A) atom occurs mostly in the direction towards bonds with oxygen atoms. In the case of Cu-O and Na-O bonds, we do not observe any notable changes connected with the phase transition (Fig S5a, S5b).

In the case of Laplacian maps for oxygen atoms, the blue regions represent the core shell charge depletion (CSCD) and red regions are the valence shell charge concentration (VSCC). Surprisingly, we observed significant deformation for valence shell of the O(4) oxygen atom with appearance of charge depletion regions along the O(4)-Cu(1) bonds at 1.08 GPa (Fig. 5a). These changes are caused by a charge transfer from disordered hydrogen atoms to oxygen atoms or can be triggered by the metastability of the structure right before the phase transition. Additionally, at 1.08 GPa, similar valence shell (VSCC) deformation is visible for oxygen atom O(1) (Fig. 5a).

Experimental deformation density maps revealed a significant charge depletion (red regions) in the copper valence shell along Cu-O bonds (Fig. S7). These regions are stable in the whole pressure range and did not change their shape or volume. The blue regions around Cu(1) atom correspond with the charge concentration which is connected with spin density. In this case we observed significant shape deformations for blue contours at 3.06 GPa and 3.65 GPa along with their volume decrease. These changes are associated with charge outflow from copper atom (Fig. S2a) towards oxygen O(3) (Fig S3c) with elevated pressure (experimental values). It leads to significant increase in the volume of blue iso-contour (charge concentration) around O(3) oxygen atom at 3.65 GPa which represents its lone pair (Fig. S7).

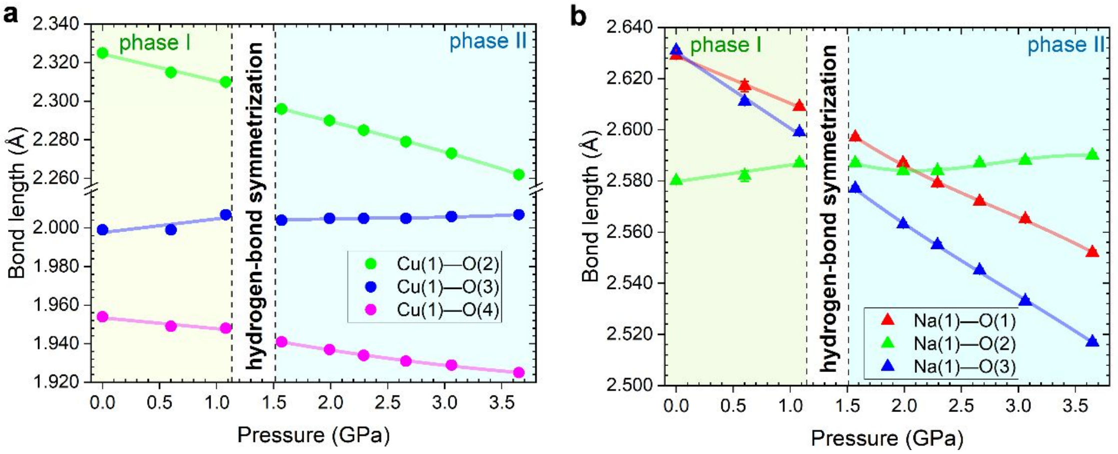

Fig. S5 Structural changes for natrochalcite as a function of pressure. (a) Changes in the bond lengths between copper and oxygen atoms as a function of pressure. (b) Changes in the bond lengths between sodium and oxygen atoms as a function of pressure. For all graphs the white square represents the pressure region where phase transition driven by hydrogen bond symmetrization occurs and error bars represent the standard deviations.

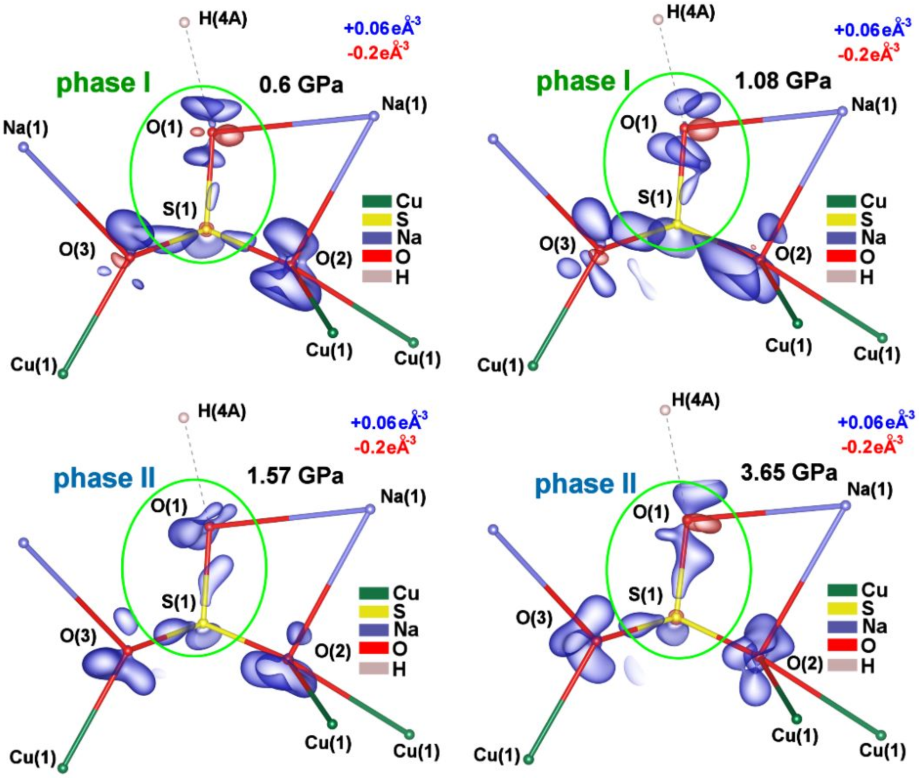

Fig. S6 3D maps of static deformation density obtained from the experimental electron density distribution for sulfate molecule in the structure of natrochalcite. Blue and red colors indicate positive and negative regions of electron density, respectively and reveal lone and bonding electron pairs. Positive iso-contours are at 0.06 eÅ^{-3} and negative iso-contours are at 0.2 eÅ^{-3}. The green ellipsoid indicates the S(1)-O(1) bond.

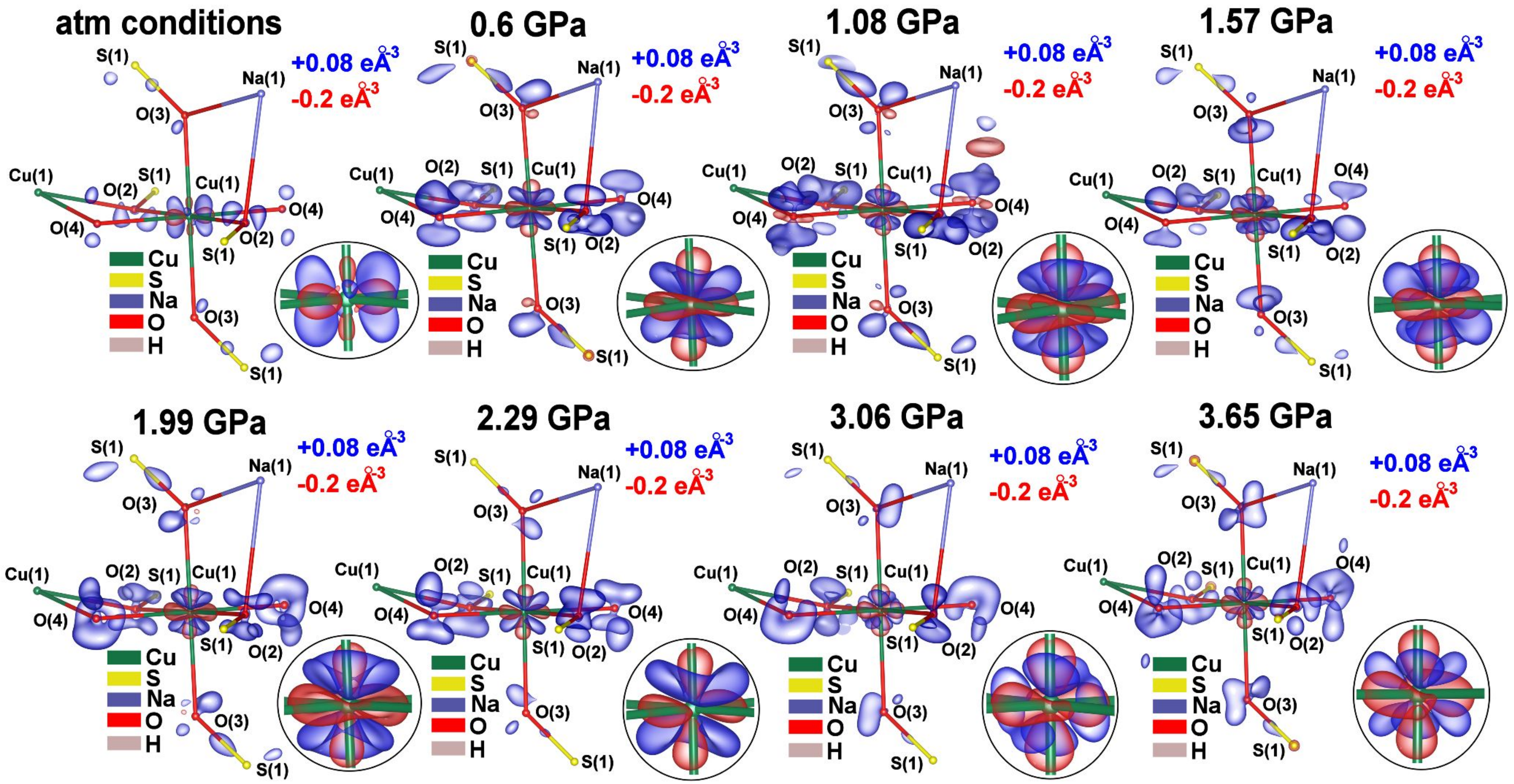

Fig. S7 3D maps of static deformation density obtained from the experimental electron density distribution for CuO_6 octahedra in the structure of natrochalcite. Blue and red colors indicate positive and negative regions of electron density, respectively and reveal lone and bonding electron pairs. Positive iso-contours are at 0.06 eÅ^{-3} and negative iso-contours are at 0.2 eÅ^{-3}.

Section 7: A complete set of 3D maps of negative Laplacian and static deformation density for natrochalcite

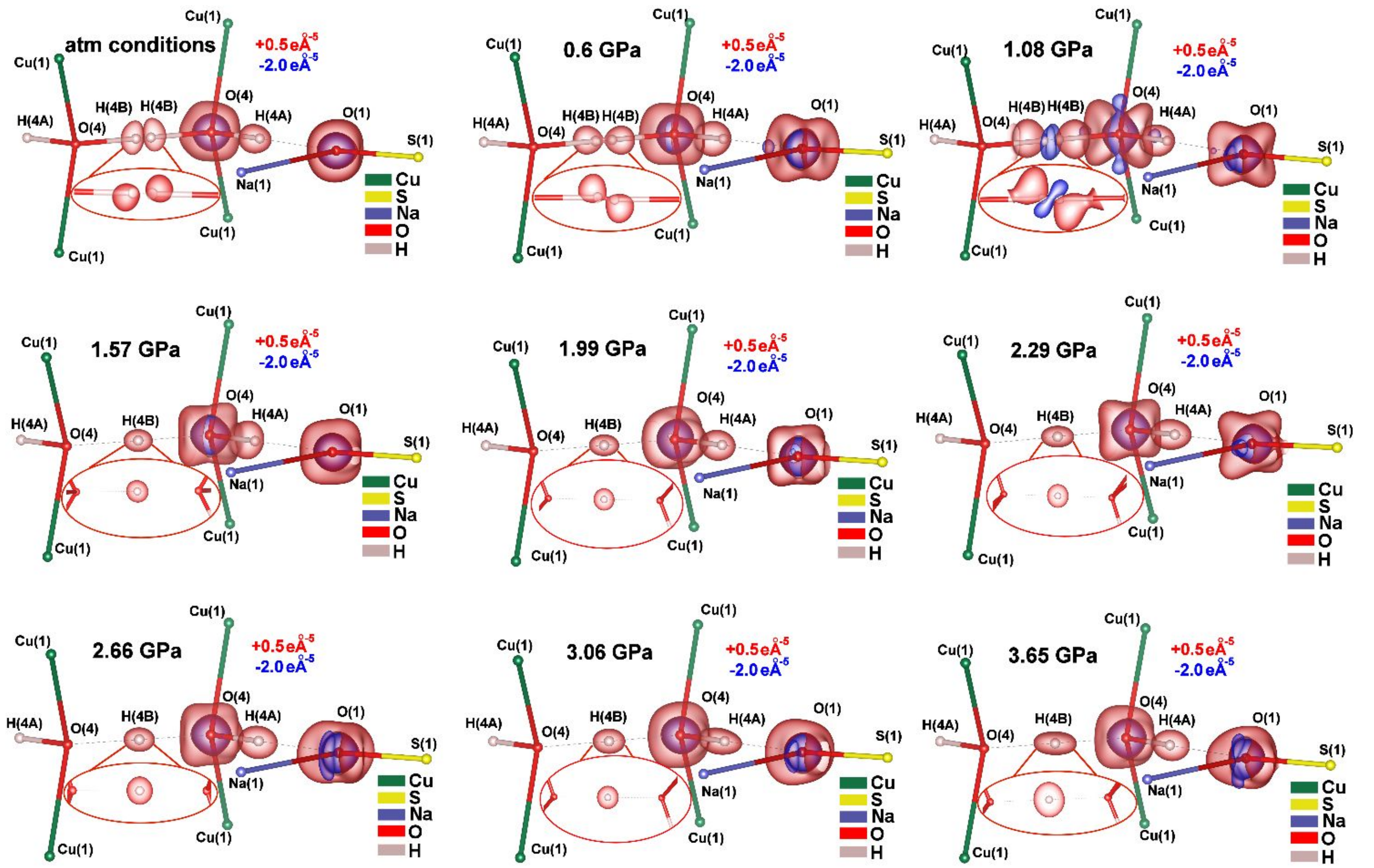

Fig. S8 3D maps of negative Laplacian obtained from the experimental electron density distribution for both hydrogen bonds in the structure of natrochalcite. Red contours showing regions of charge concentration and iso-contours are at 0.5 eÅ^{-5}. Blue iso-contours showing regions of charge depletion and contours are at 2.0 eÅ^{-5}.

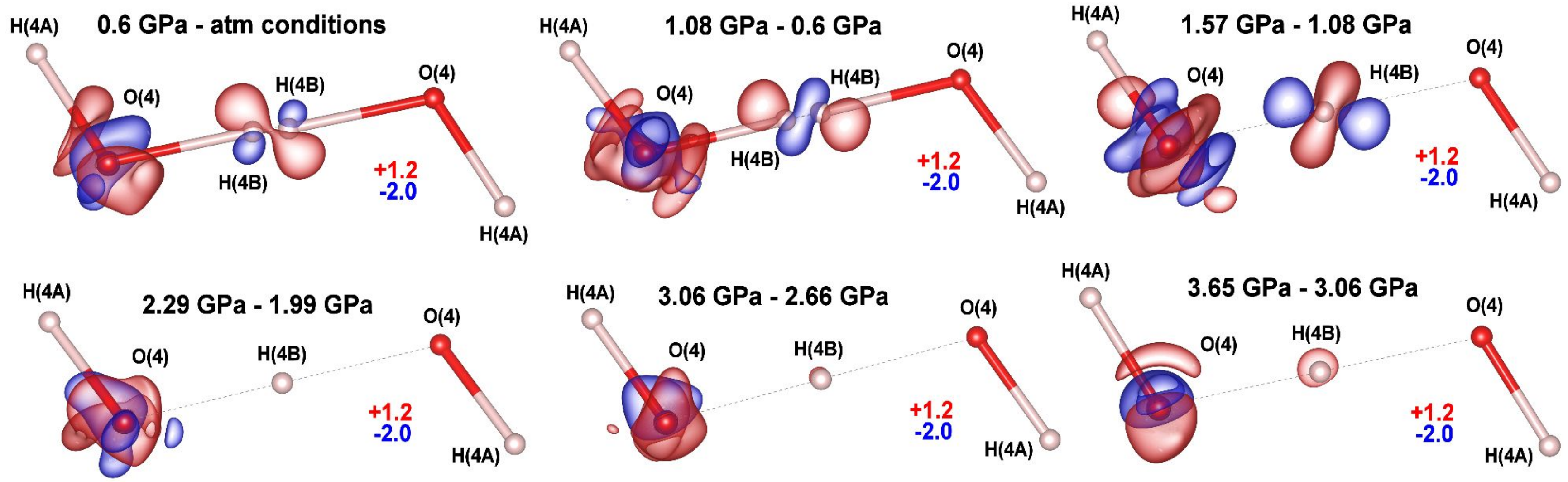

Fig. S9 3D maps of differences in negative Laplacian values for the O(4) oxygen and H(4B) hydrogen. These maps represent the difference in charge concentration and depletion between pressure points defined at the top of particular figures. Red contours show regions of charge concentration and iso-contours are at + 1.2 eÅ^{-5}. Blue contours showing regions of charge depletion and iso-contours are at - 2.0 eÅ^{-5}

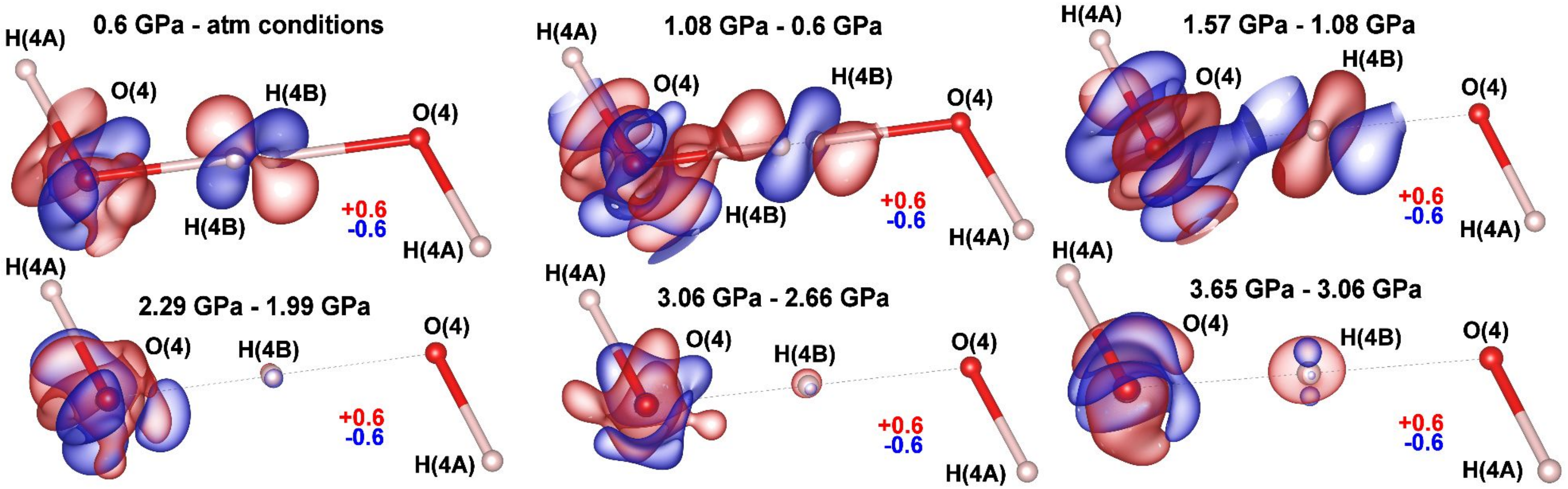

Fig. S10 3D maps of differences in negative Laplacian values for the O(4) oxygen and H(4B) hydrogen. These maps represent the difference in charge concentration and depletion between pressure points defined at the top of particular figures. Red contours show regions of charge concentration and iso-contours are at + 0.6 eÅ^{-5}. Blue contours showing regions of charge depletion and iso-contours are at – 0.6 eÅ^{-5}

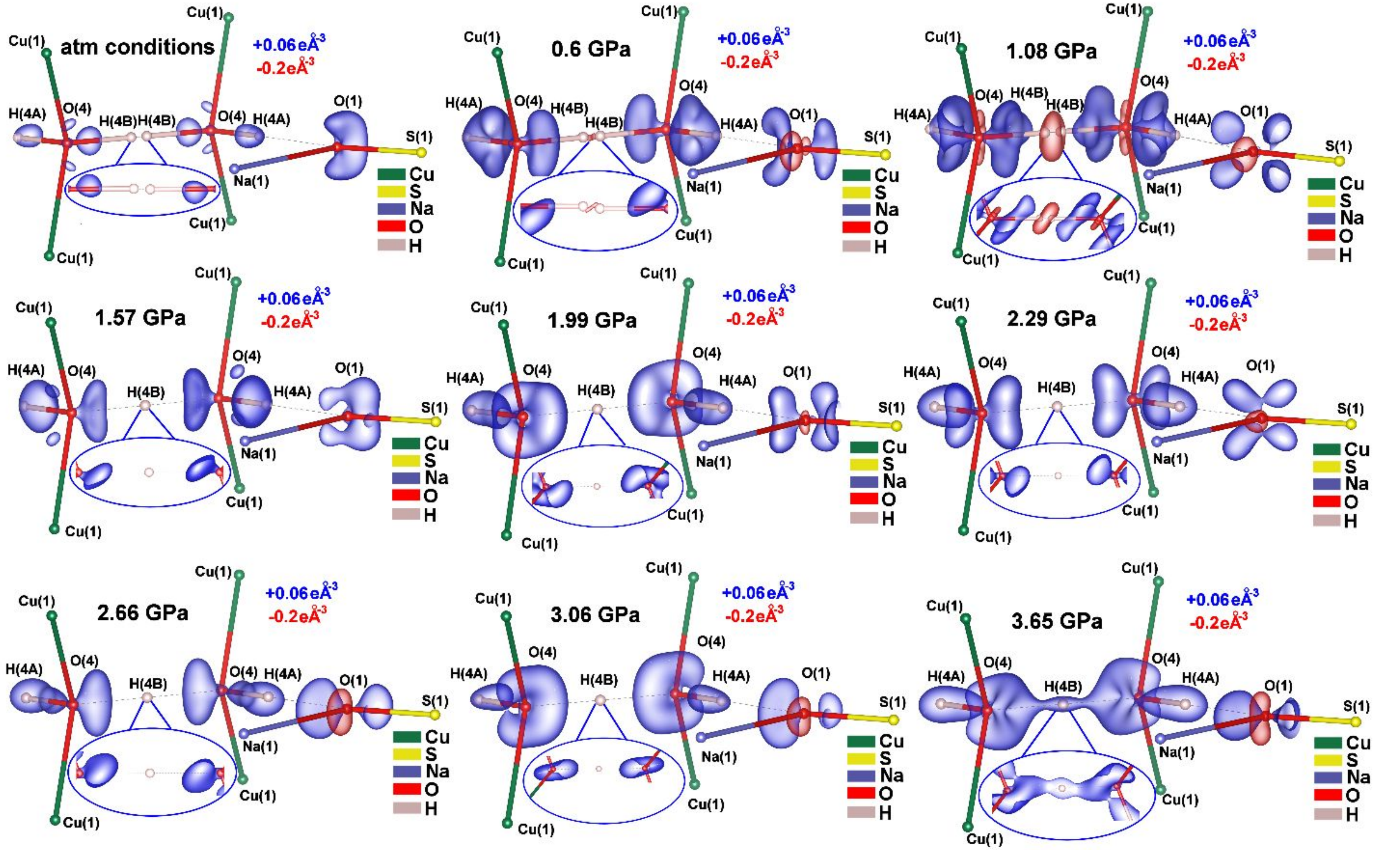

Fig. S11 3D maps of static deformation density obtained from the experimental electron density distribution for both hydrogen bonds in the structure of natrochalcite. Blue and red colors indicate positive and negative regions of electron density, respectively and reveal lone and bonding electron pairs. Positive iso-contours are at 0.06 eÅ^{-3} and negative iso-contours are at 0.2 eÅ^{-3}

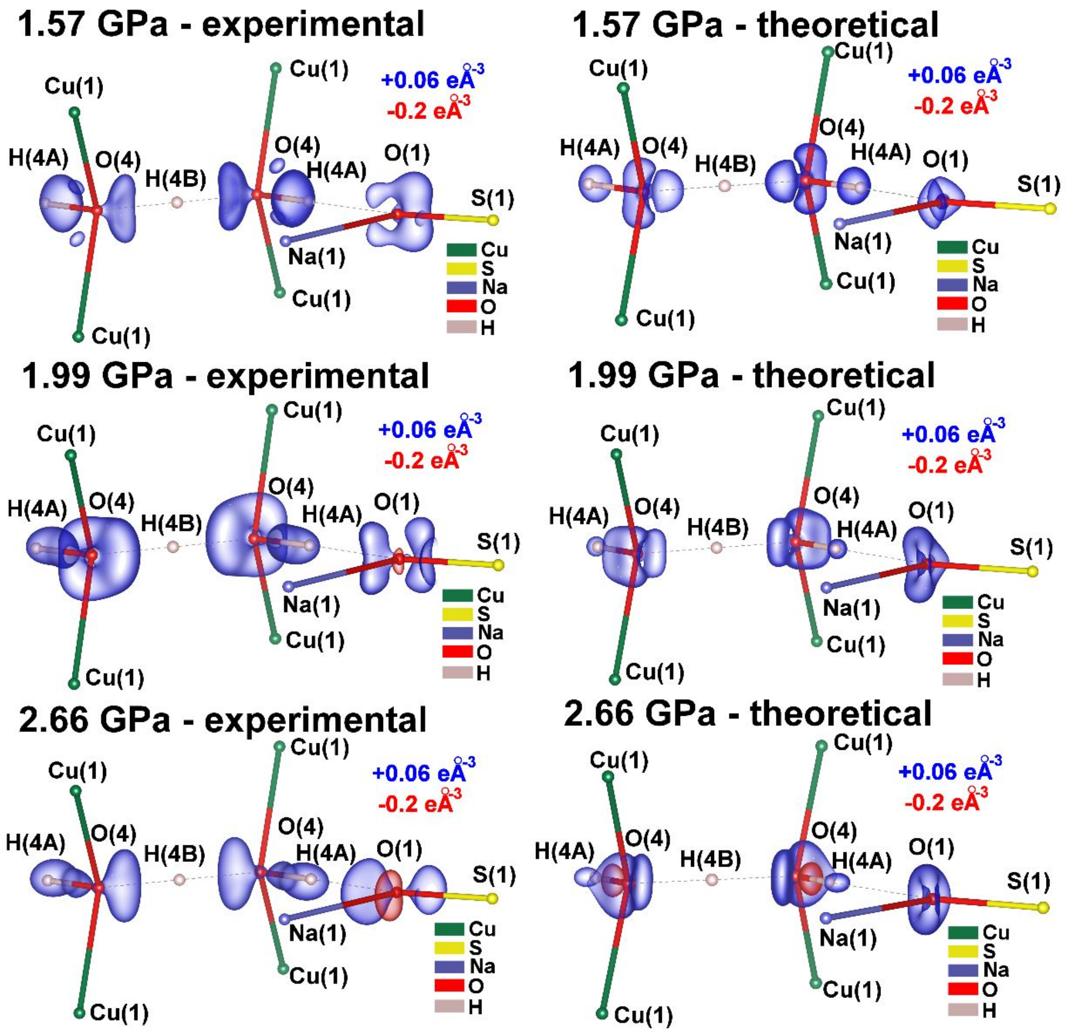

Fig. S12 3D maps of static deformation density obtained from the experimental and theoretical electron density distribution for both hydrogen bonds in the structure of natrochalcite. Blue and red colors indicate positive and negative regions of electron density, respectively and reveal lone and bonding electron pairs. Positive iso-contours are at 0.06 eÅ^{-3} and negative iso-contours are at 0.2 eÅ^{-3}

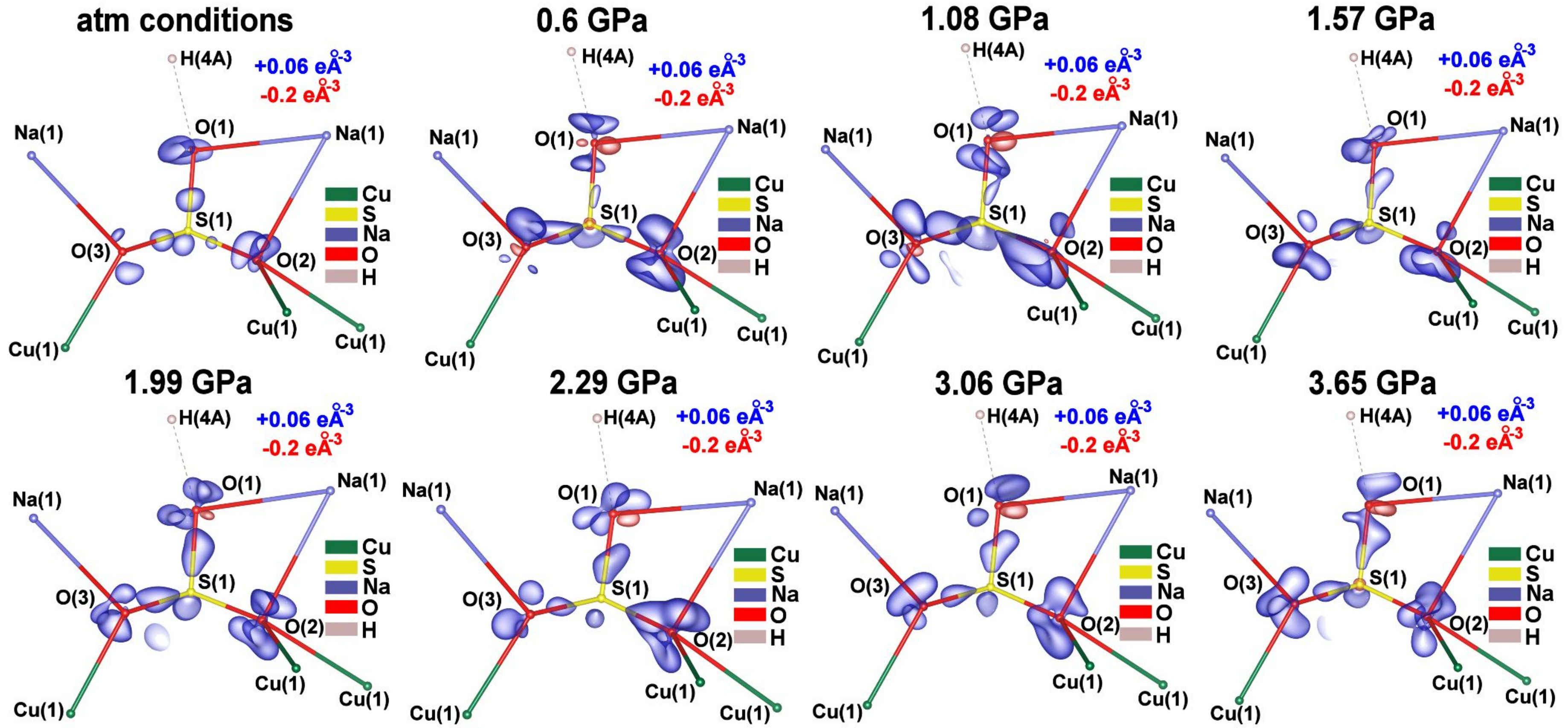

Fig. S13 3D maps of static deformation density obtained from the experimental electron density distribution for sulfate molecule in the structure of natrochalcite. Blue and red colors indicate positive and negative regions of electron density, respectively and reveal lone and bonding electron pairs. Positive iso-contours are at 0.06 eÅ^{-3} and negative iso-contours are at 0.2 eÅ^{-3}.

Section 8: A complete set of integrated atomic basins for natrochalcite

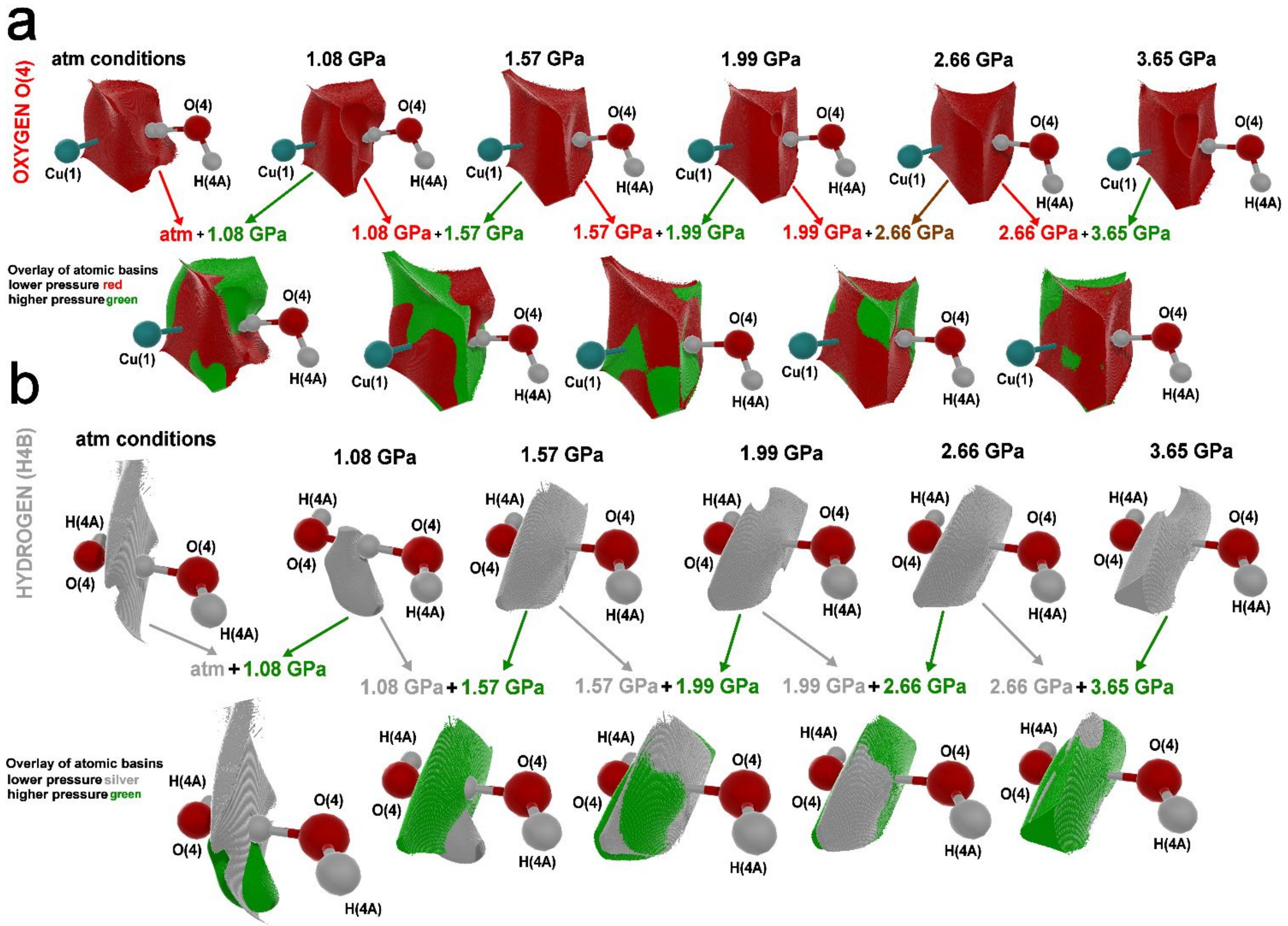

Fig.S14 A complete set of atomic basins for atoms from O(4)-H(4B)···O(4) moiety under high pressure conditions. (a) Shape changes of oxygen O(4) atomic basin influenced by a high pressure. (b) Shape changes of hydrogen H(4B) atomic basin influenced by a high pressure. Atomic basins at lower pressure are superimposed on the atomic basins at higher pressure colored in green. Parts where green color is on the top reveal volume expansion due to increasing pressure and in the remaining fragments the electron density of atom (and hence volume) is compressed.

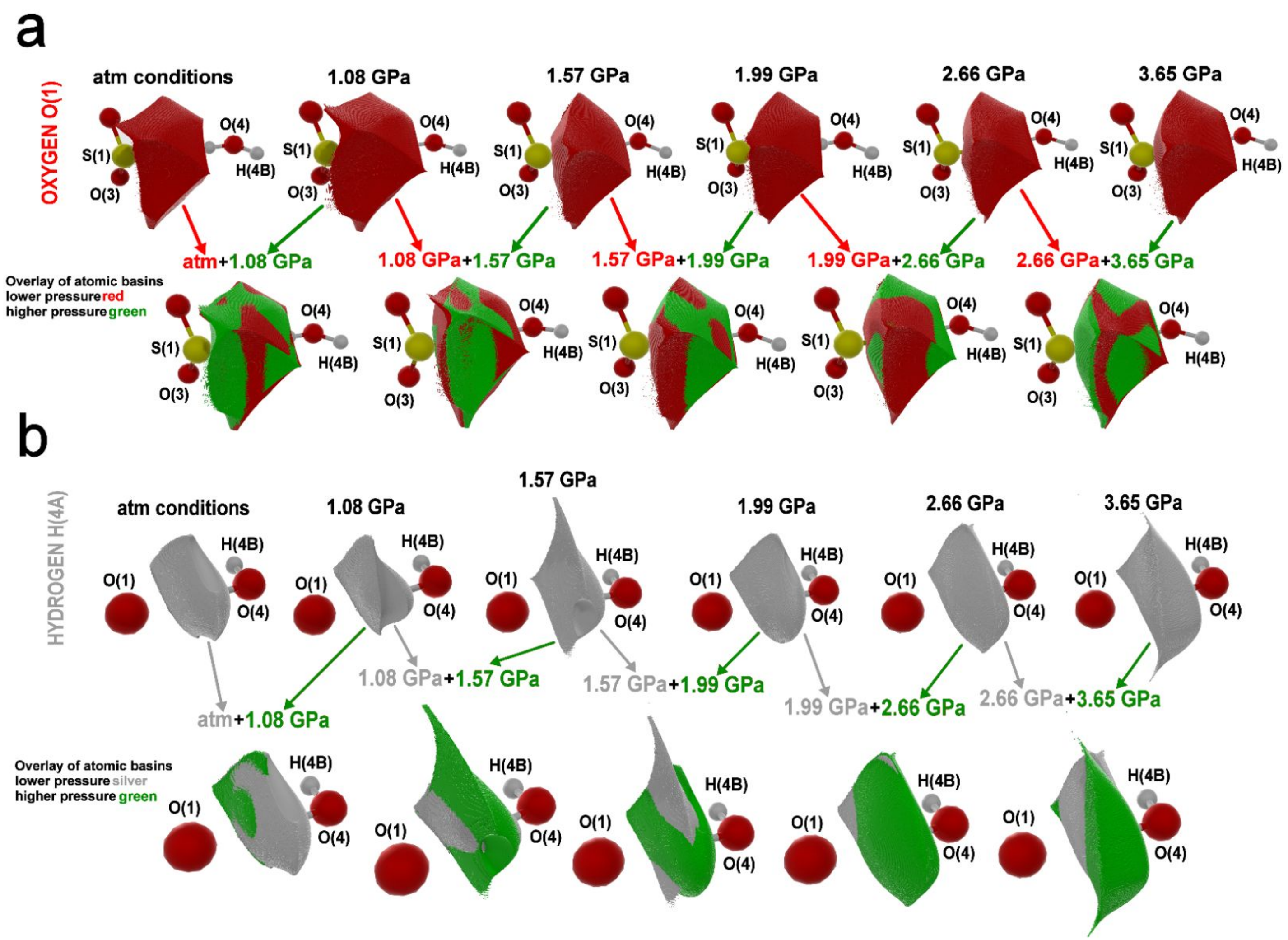

Fig. S15 Atomic basins for atoms from O(4)-H(4A)···O(1) moiety under high pressure conditions. (a) Shape changes of oxygen O(1) atomic basin influenced by a high pressure. (b) Shape changes of hydrogen H(4A) atomic basin influenced by a high pressure. Atomic basins at lower pressure are superimposed on the atomic basins at higher pressure colored in green. Parts where green color is on the top reveal volume expansion due to increasing pressure and in the remaining fragments the electron density of atom (and hence volume) is compressed.

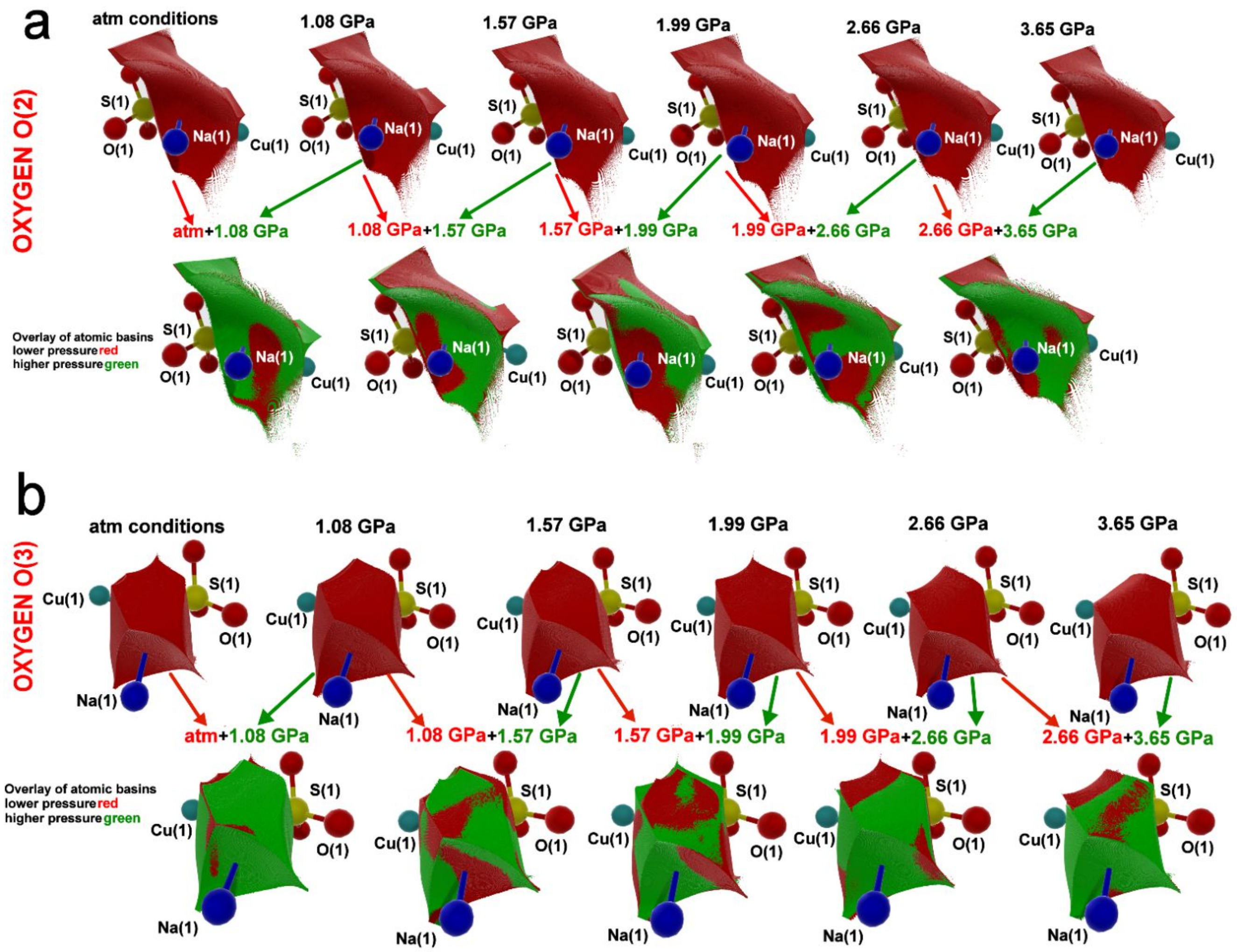

Fig. S16 Atomic basins for oxygen atoms O(2) and O(3) under high pressure conditions. (a) Shape changes of oxygen O(2) atomic basin influenced by a high pressure. (b) Shape changes of oxygen O(3) atomic basin influenced by a high pressure. Atomic basins at lower pressure are superimposed on the atomic basins at higher pressure colored in green. Parts where green color is on the top reveal volume expansion due to increasing pressure and in the remaining fragments the electron density of atom (and hence volume) is compressed.

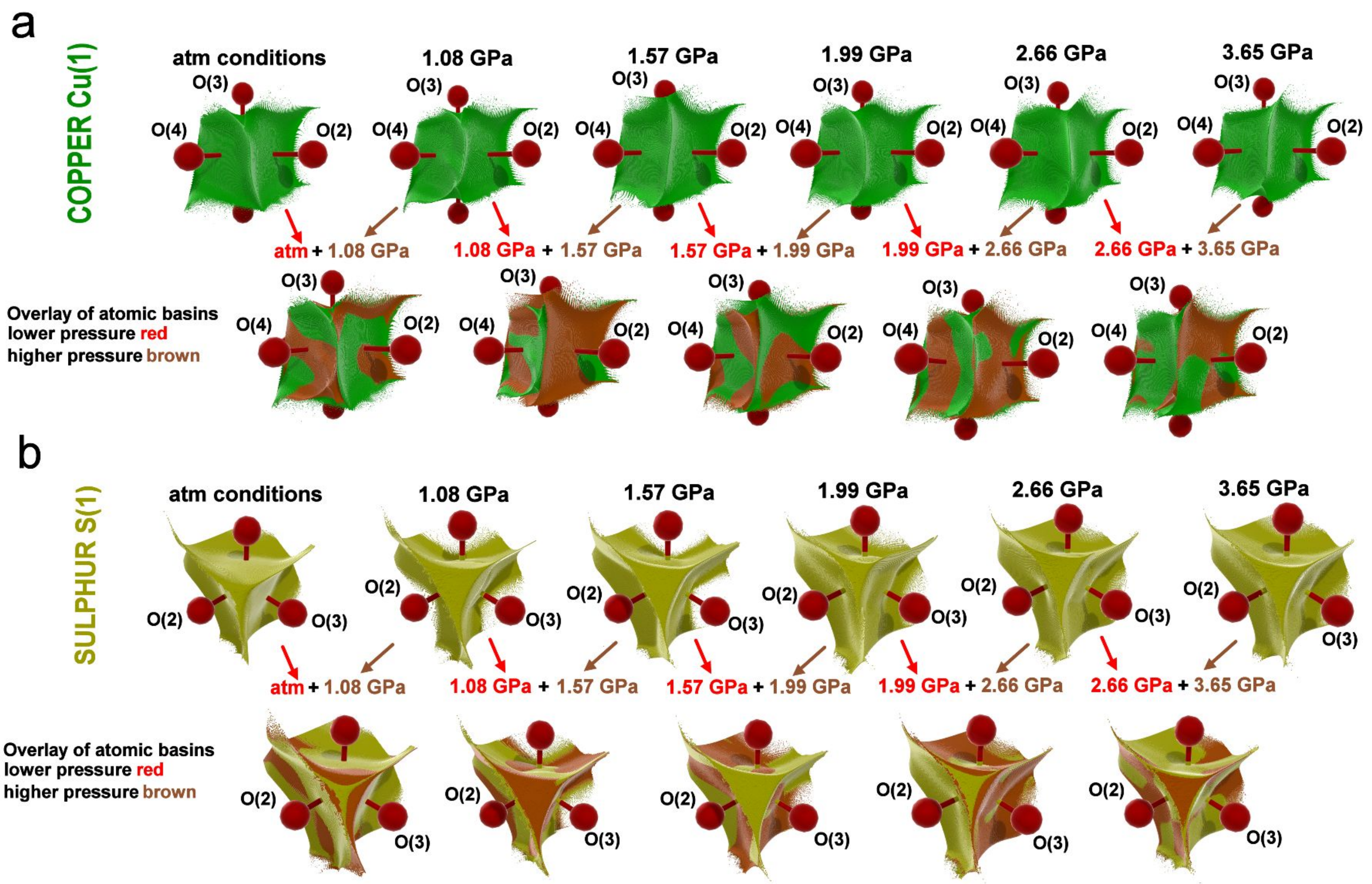

Fig. S17 Atomic basins for copper Cu(1) and sulfur S(1) atoms under high pressure conditions. (a) Shape changes of copper Cu(1) atomic basin influenced by a high pressure. (b) Shape changes of sulfur S(1) atomic basin influenced by a high pressure. Atomic basins at lower pressure are superimposed on the atomic basins at higher pressure colored in brown. Parts where brown color is on the top reveal volume expansion due to increasing pressure and in the remaining fragments the electron density of atom (and hence volume) is compressed.

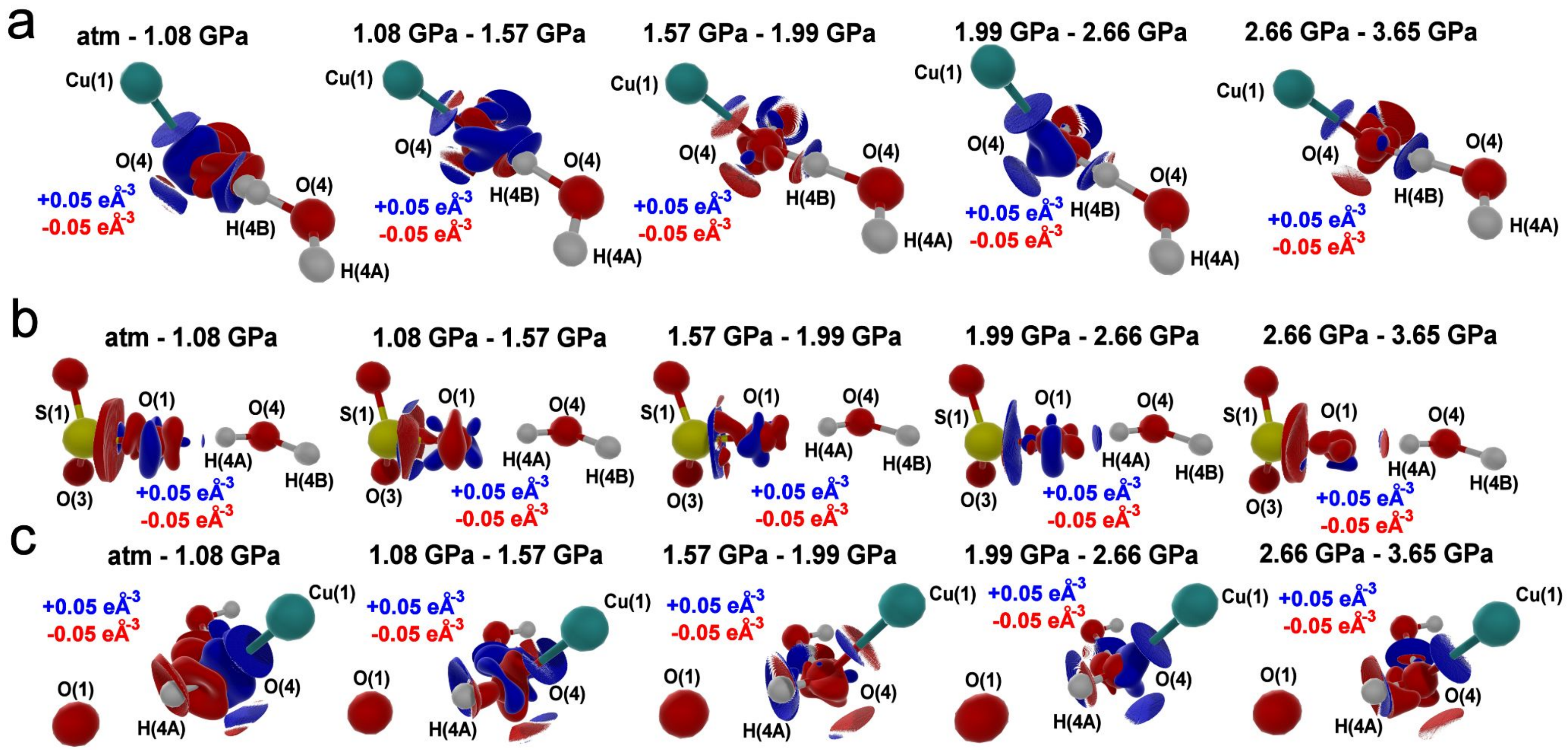

Fig. S18 Changes of electron density inside atomic basins of atoms from H_2O moiety. (a) Redistribution of electron density inside oxygen O(4) atomic basins with elevated pressure from the perspective of O(4)-H(4B)···O(4) hydrogen bond. (b) Redistribution of electron density inside oxygen O(1) atomic basins with elevated pressure. (c) Redistribution of electron density inside oxygen O(4) atomic basins with elevated pressure from the perspective of O(4)-H(4A)···O(1) hydrogen bond.

Changes in the shapes of oxygens O(2) and O(3) atomic basins (Fig S16) are not as spectacular as for oxygens O(4) (FigS14) and O(1) (Fig S15). It shows that the phase transition based on the process of hydrogen bond symmetrization affects only oxygens atomic basins which participate in the formation of hydrogen bonds in the structure of natrochalcite. In the case of copper Cu(1) and sulfur S(1) we also did not observe significant changes in the shapes of their atomic basins (Fig. S17). However we can say that both atoms (Cu and S) have the most regular shapes among all atomic basins in natrochalcite. Copper basins are most similar to cubes with six faces because there are six oxygen ligands around central atom in the CuO_6 octahedra and the S basins to tetrahedra with four faces (four oxygen atoms around sulfur atom). Electron density also redistributes inside atomic basins when external stimuli, such as a pressure, are applied. The differential density maps (where the electron density values from atomic basins at higher pressure are subtracted at every point from the ones at lower pressure) give a better insight how EDD changes with pressure closer to atomic nuclei or bonds (Fig. S18). The differential density maps of bihydroxide anion show similar changes in the electron density redistribution as negative Laplacian maps and charge flow from hydrogen H(4B) towards oxygen O(4) is observed in the phase I (red region for atm – 1.08 GPa map, Fig. S18a). However, after the phase transition the opposite situation takes place and charge depletion occurs for oxygen O(4) especially along Cu(1)-O(4) bond which is visible on the 1.99 – 2.66 GPa differential density map. It leads to significant volume decrease for O(4) oxygen atomic basin (Fig. S3d). We also observed on the difference density maps for O(1) atom (Fig S16b) that charge transfer between O(1) and S(1) is a continuous process and increases with increasing pressure up to 2.66 GPa. Additionally the redistribution density maps inside atomic basins revealed increasing charge concentration at O(4)-H(4A) bond along with elevated pressure and it is connected with strengthening of that covalent bond when pressure is applied (Fig.S18c, Table S4, S6).

Section 9: Comparison of experimental results for aspherical atom models at various pressure conditions by the analysis of residual electron density

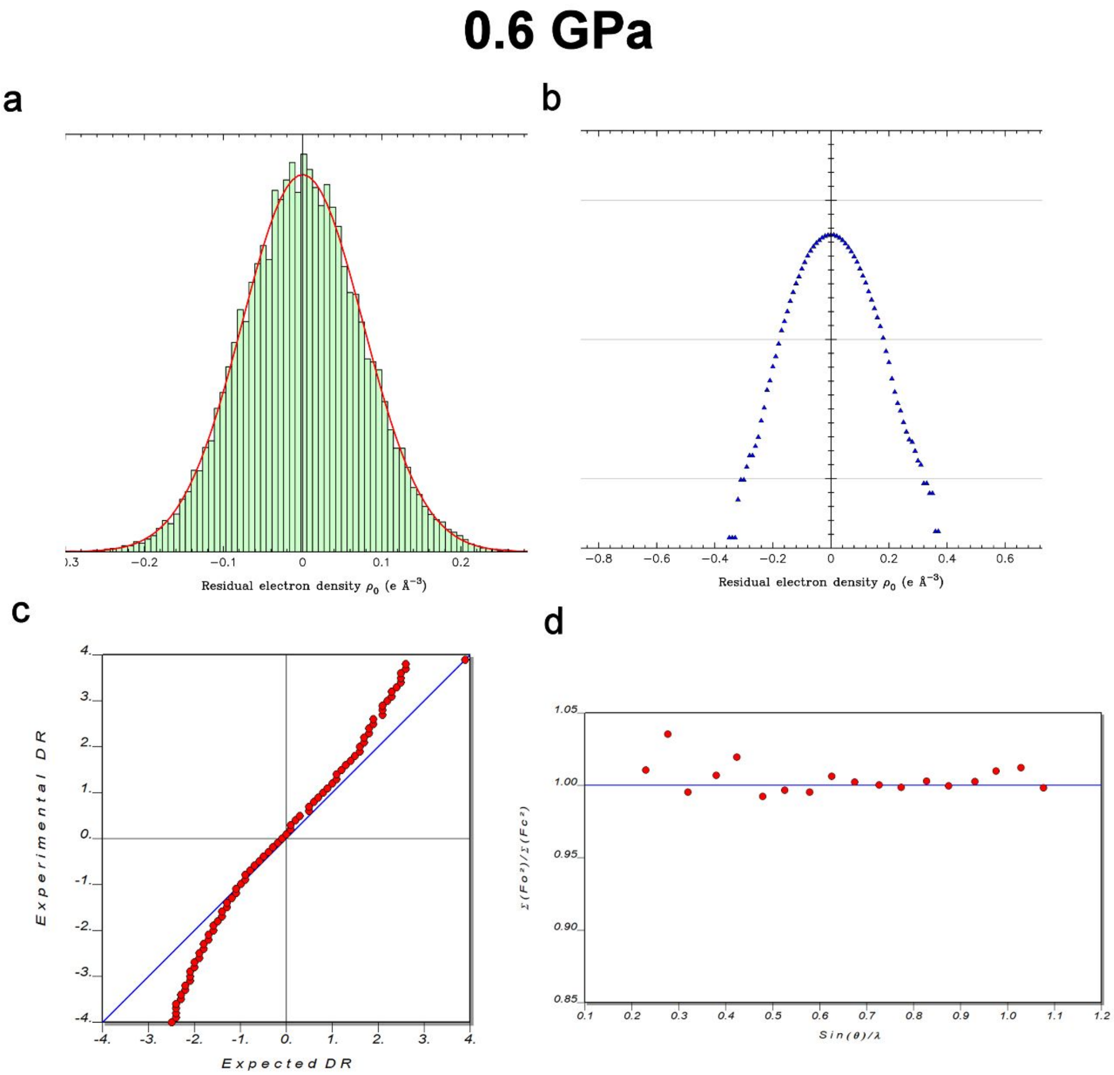

Fig. S19 Experimental results for aspherical atom model at 0.6 GPa. (a) Probability distribution histogram. (b) Fractal dimension plot. (c) Normal probability plot. (d) $\Sigma(Fo^2)/\Sigma(Fc^2)$ as a function of sinθ/λ. The graphs were generated for the multipole model refined against high resolution (sin θ/λ = 1.1 Å^{-1}) X-ray data.

1.08 GPa

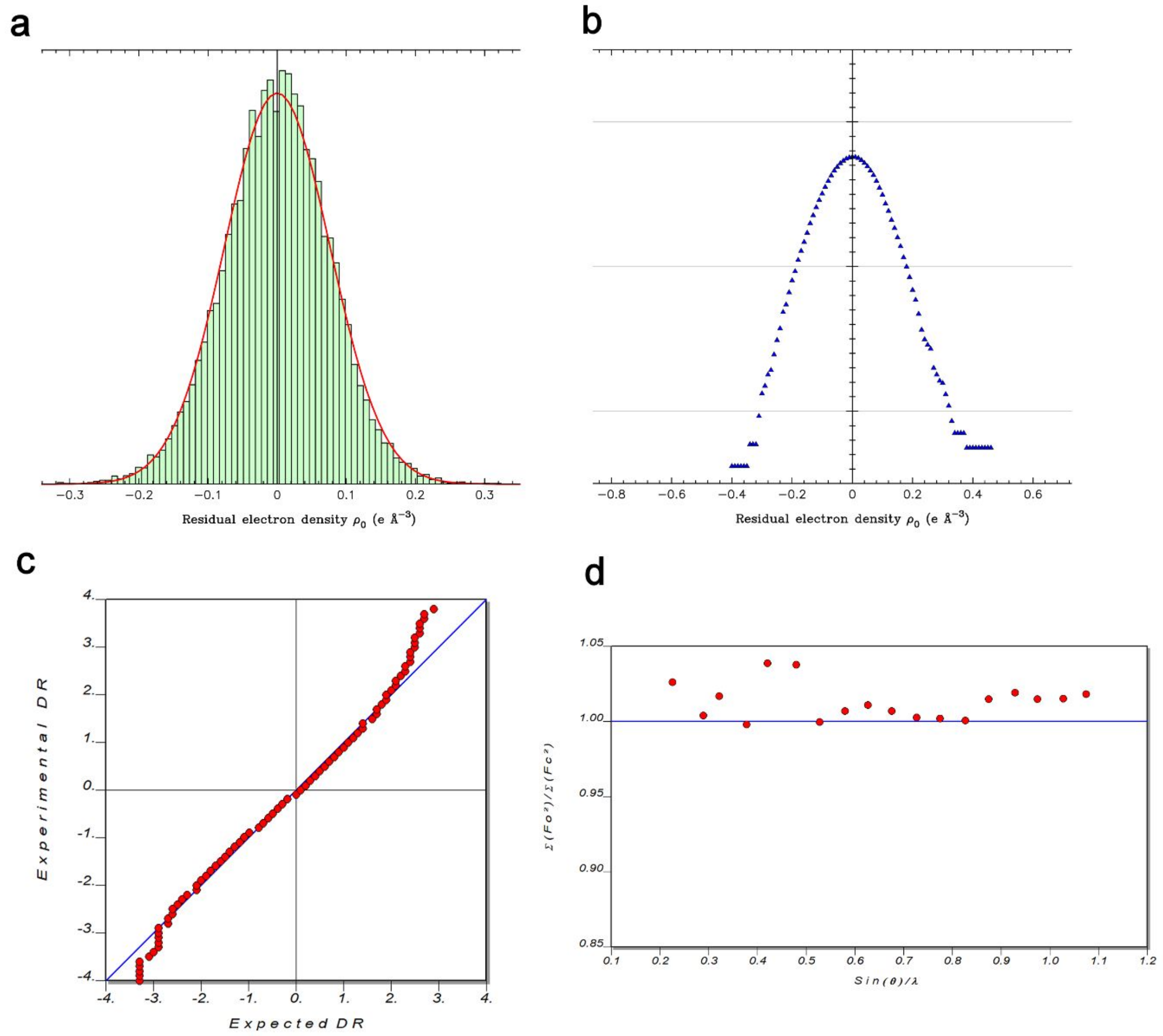

Fig. S20 Experimental results for aspherical atom model at 1.08 GPa. (a) Probability distribution histogram. (b) Fractal dimension plot. (c) Normal probability plot. (d) $\Sigma(Fo^2)/\Sigma(Fc^2)$ as a function of sinθ/λ. The graphs were generated for the multipole model refined against high resolution (sin θ/λ = 1.1 Å^{-1}) X-ray data.

1.57 GPa

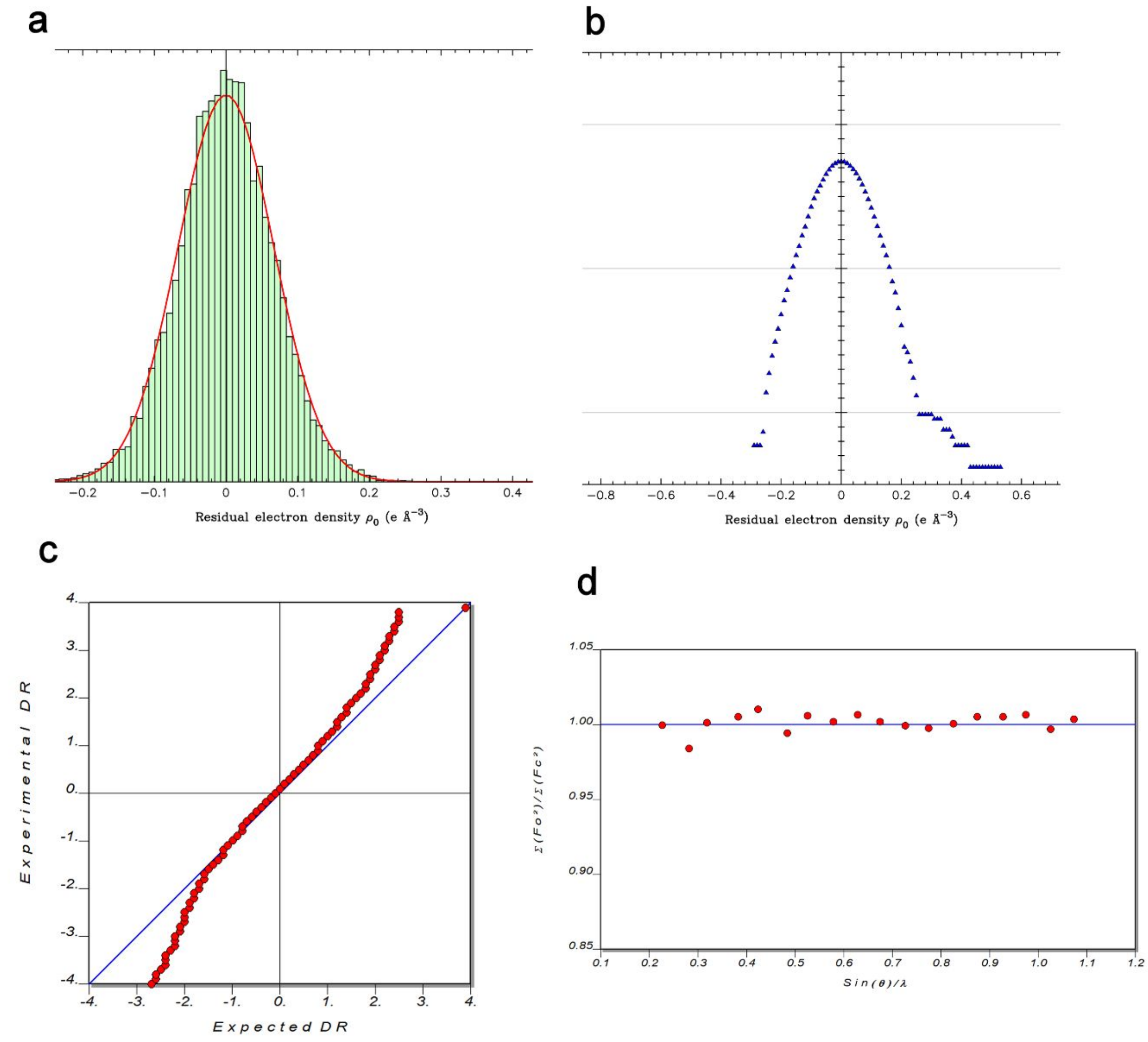

Fig. S21 Experimental results for aspherical atom model at 1.57 GPa. (a) Probability distribution histogram. (b) Fractal dimension plot. (c) Normal probability plot. (d) $\Sigma(Fo^2)/\Sigma(Fc^2)$ as a function of sinθ/λ. The graphs were generated for the multipole model refined against high resolution (sin θ/λ = 1.1 Å^{-1}) X-ray data.

1.99 GPa

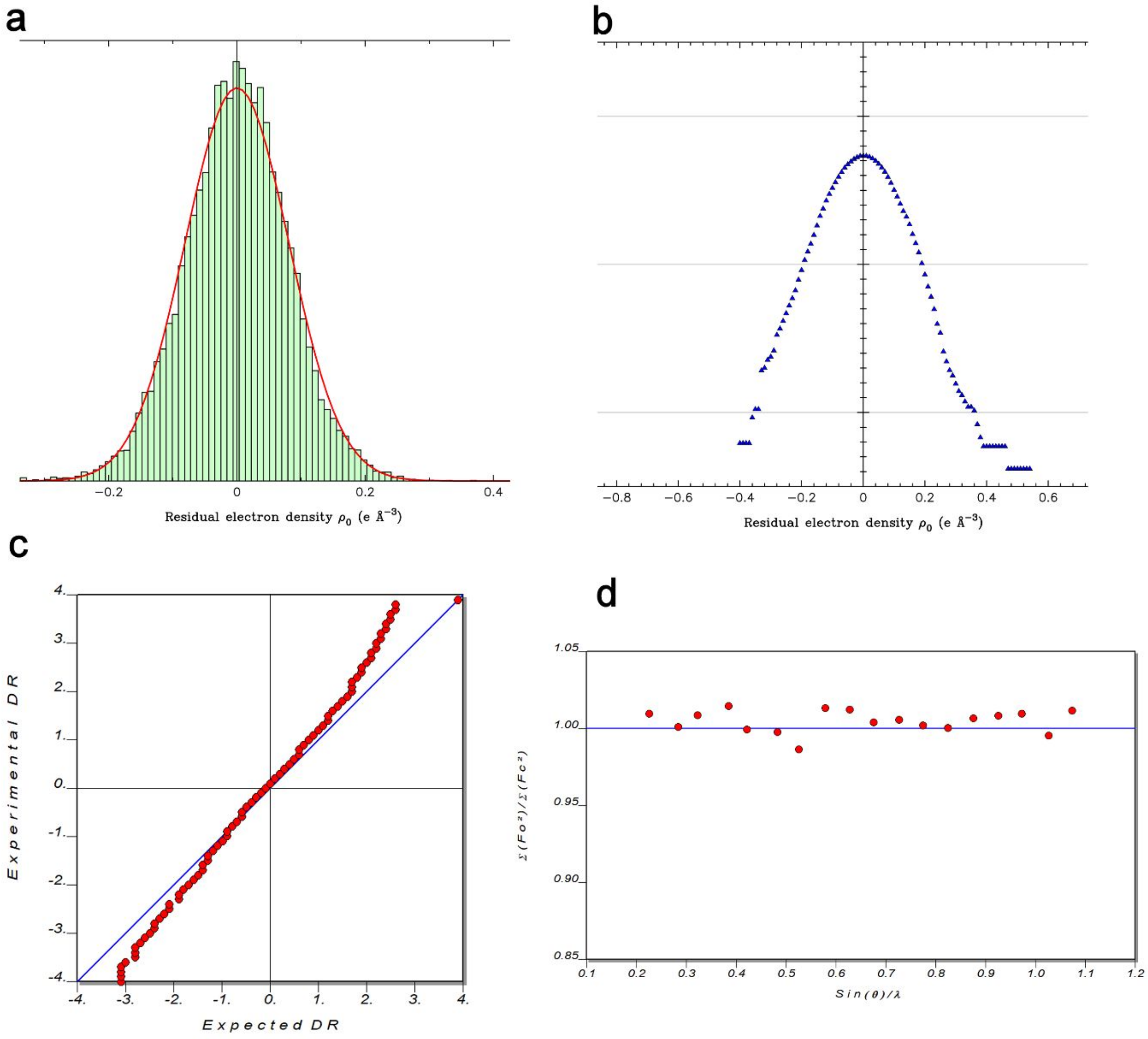

Fig. S22 Experimental results for aspherical atom model at 1.99 GPa. (a) Probability distribution histogram. (b) Fractal dimension plot. (c) Normal probability plot. (d) $\Sigma(Fo^2)/\Sigma(Fc^2)$ as a function of $\sin\theta/\lambda$. The graphs were generated for the multipole model refined against high resolution ($\sin\theta/\lambda$ = 1.1 $Å^{-1}$) X-ray data.

2.29 GPa

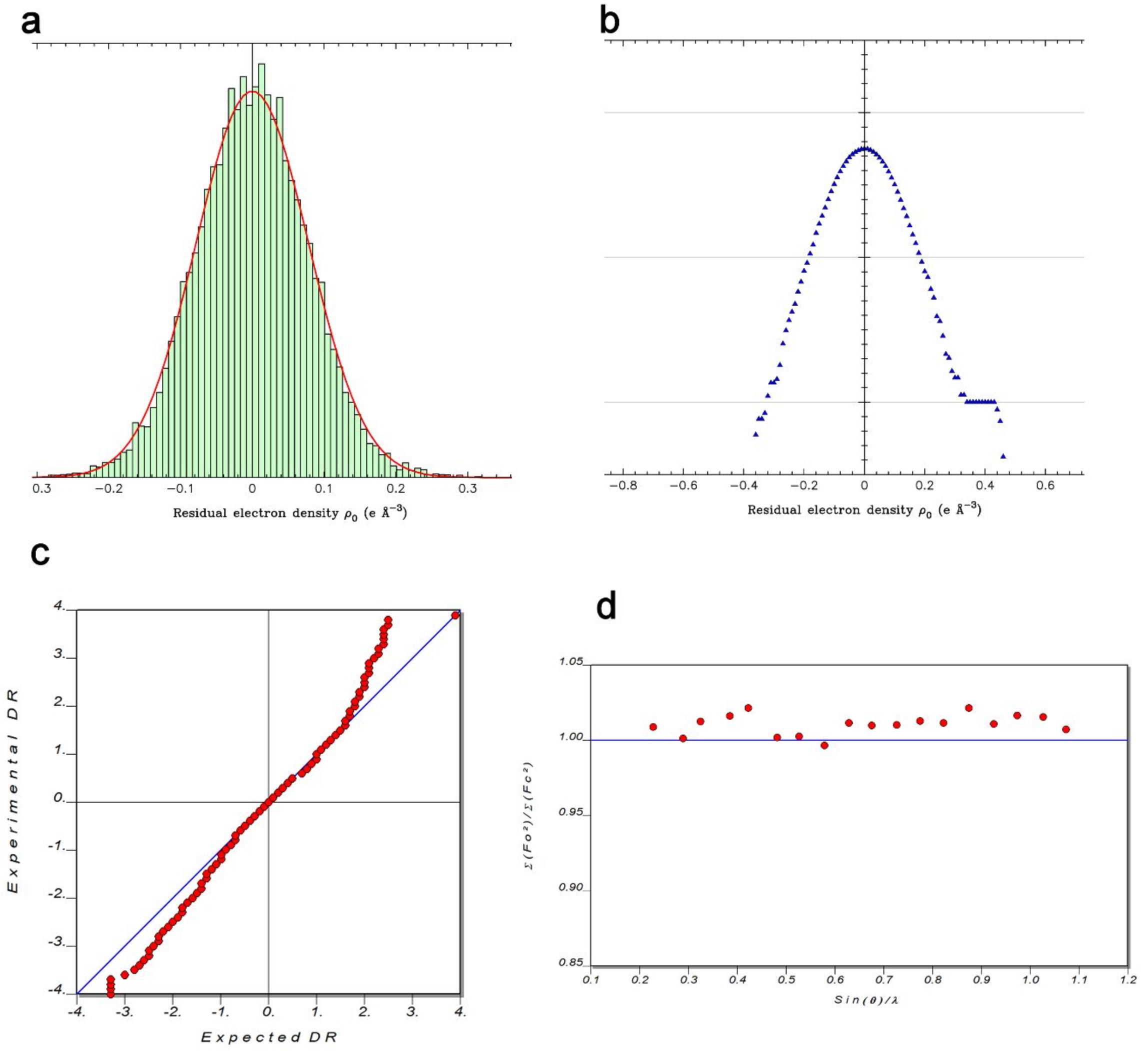

Fig. S23 Experimental results for aspherical atom model at 2.29 GPa. (a) Probability distribution histogram. (b) Fractal dimension plot. (c) Normal probability plot. (d) $\Sigma(Fo^2)/\Sigma(Fc^2)$ as a function of sinθ/λ. The graphs were generated for the multipole model refined against high resolution (sin θ/λ = 1.1 $Å^{-1}$) X-ray data.

2.66 GPa

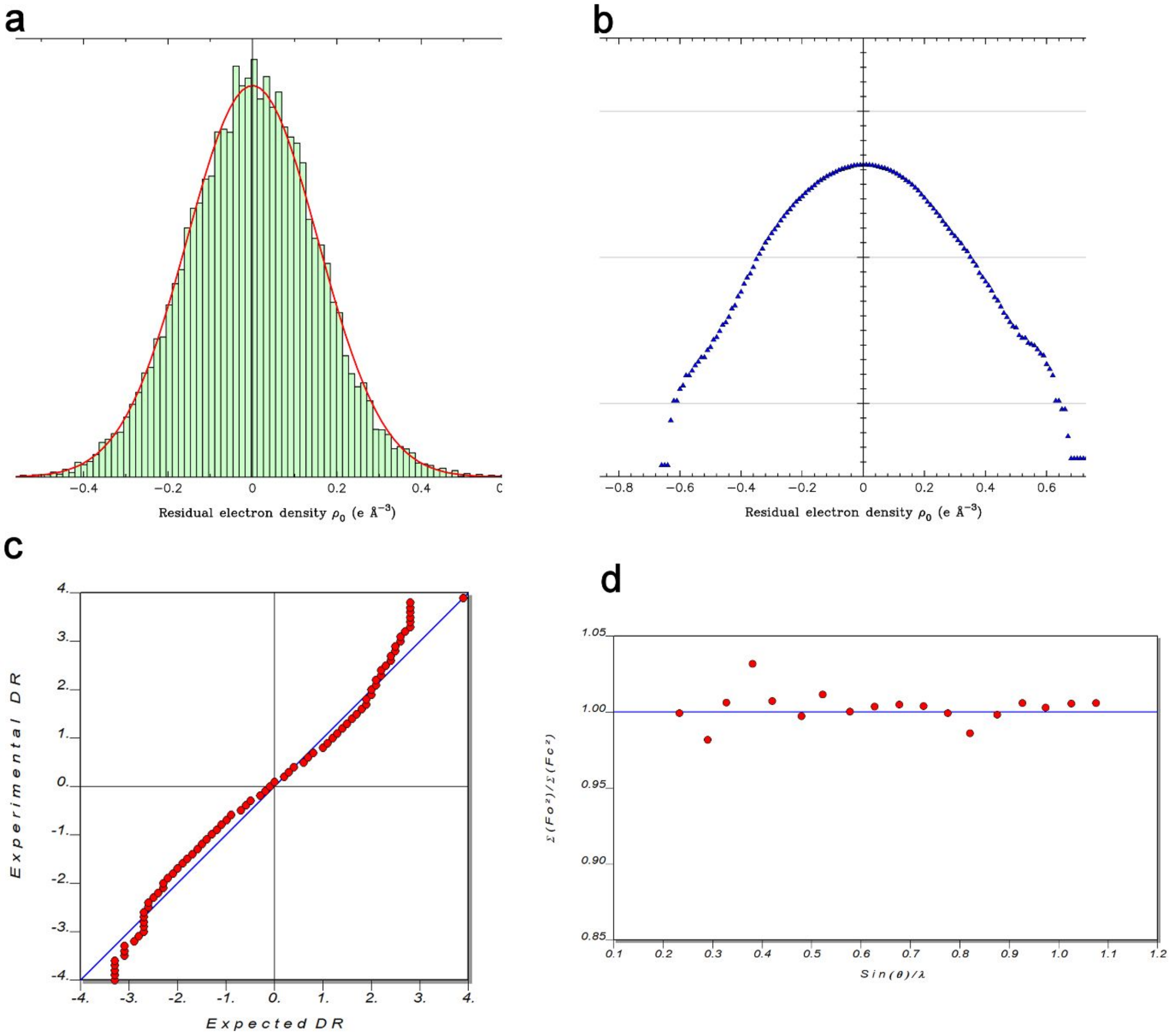

Fig. S24 Experimental results for aspherical atom model at 2.66 GPa. (a) Probability distribution histogram. (b) Fractal dimension plot. (c) Normal probability plot. (d) $\Sigma(Fo^2)/\Sigma(Fc^2)$ as a function of sinθ/λ. The graphs were generated for the multipole model refined against high resolution (sin θ/λ = 1.1 Å^{-1}) X-ray data.

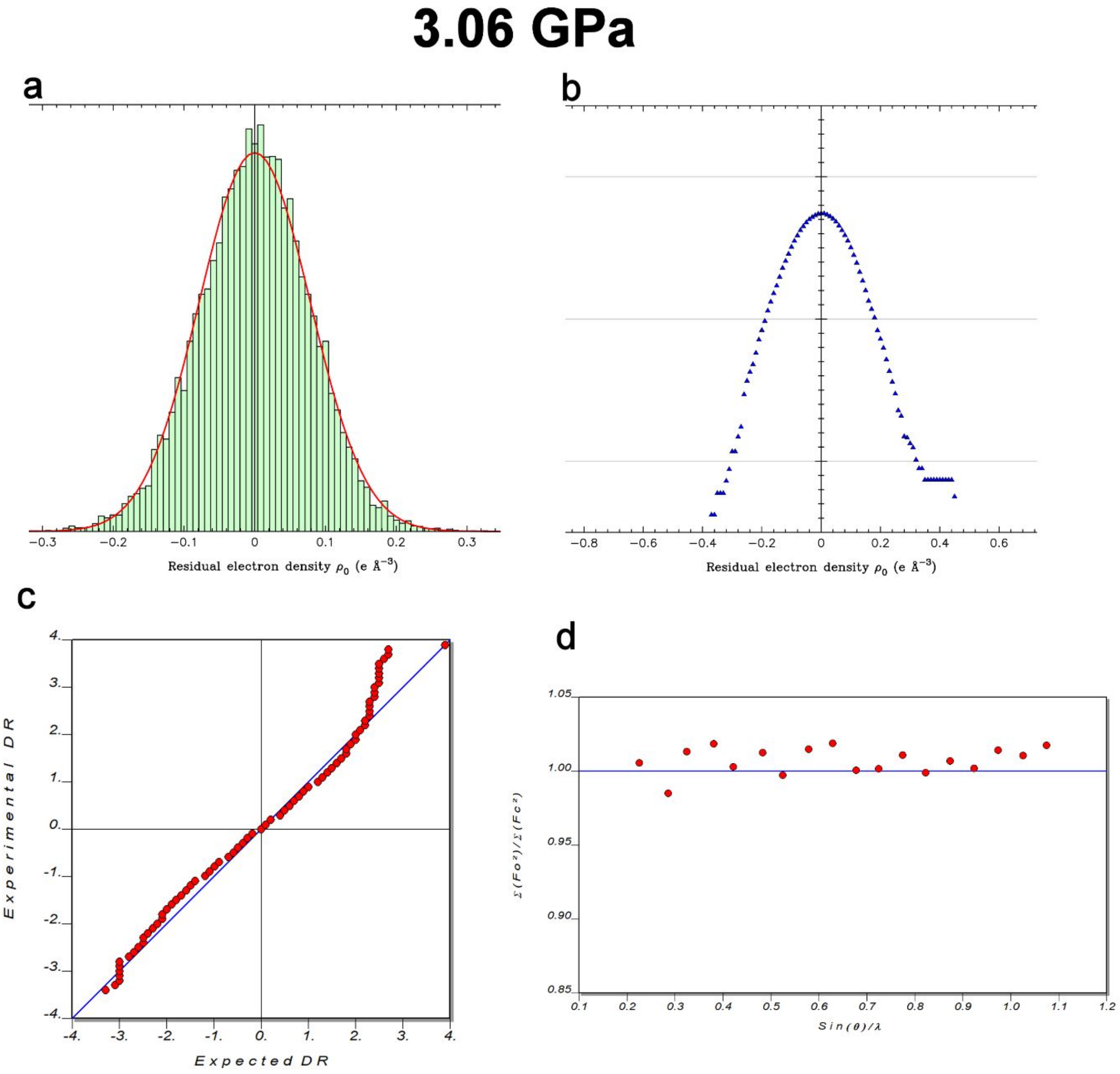

Fig. S25 Experimental results for aspherical atom model at 3.06 GPa. (a) Probability distribution histogram. (b) Fractal dimension plot. (c) Normal probability plot. (d) $\Sigma(Fo^2)/\Sigma(Fc^2)$ as a function of sinθ/λ. The graphs were generated for the multipole model refined against high resolution (sin θ/λ = 1.1 $Å^{-1}$) X-ray data.

3.65 GPa

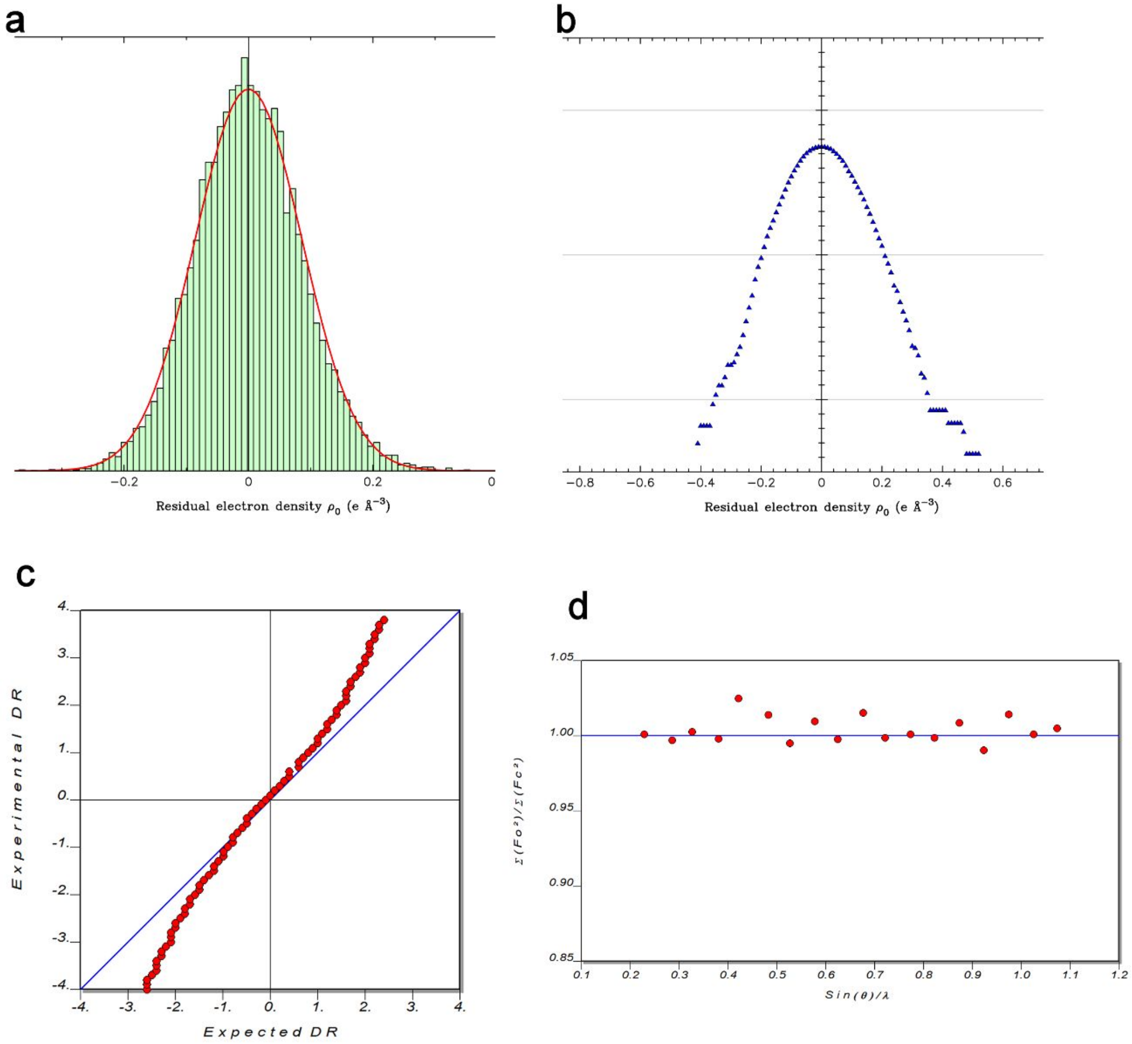

Fig. S26 Experimental results for aspherical atom model at 3.65 GPa. (a) Probability distribution histogram. (b) Fractal dimension plot. (c) Normal probability plot. (d) $\Sigma(Fo^2)/\Sigma(Fc^2)$ as a function of $\sin\theta/\lambda$. The graphs were generated for the multipole model refined against high resolution ($\sin\theta/\lambda$ = 1.1 Å^{-1}) X-ray data.

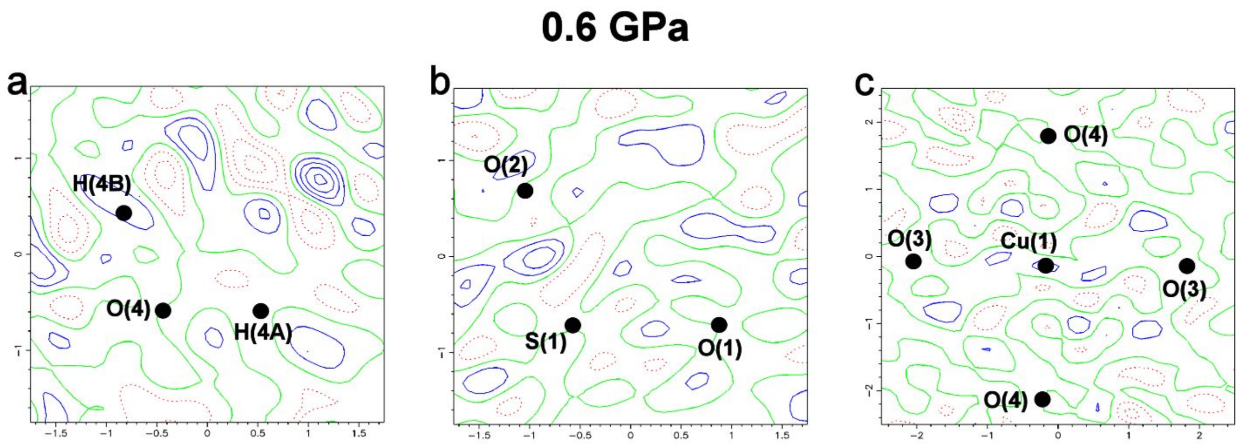

Fig. S27 2D residual electron density maps at 0.6 GPa. (a) Residual electron density map for H_2O moiety. (b) Residual electron density map for sulfate moiety. (c) Residual electron density map for CuO_6 octahedra. Positive (blue solid) and negative (red dashed) contour lines are drawn employing a step width of 0.1 $eÅ^{-3}$

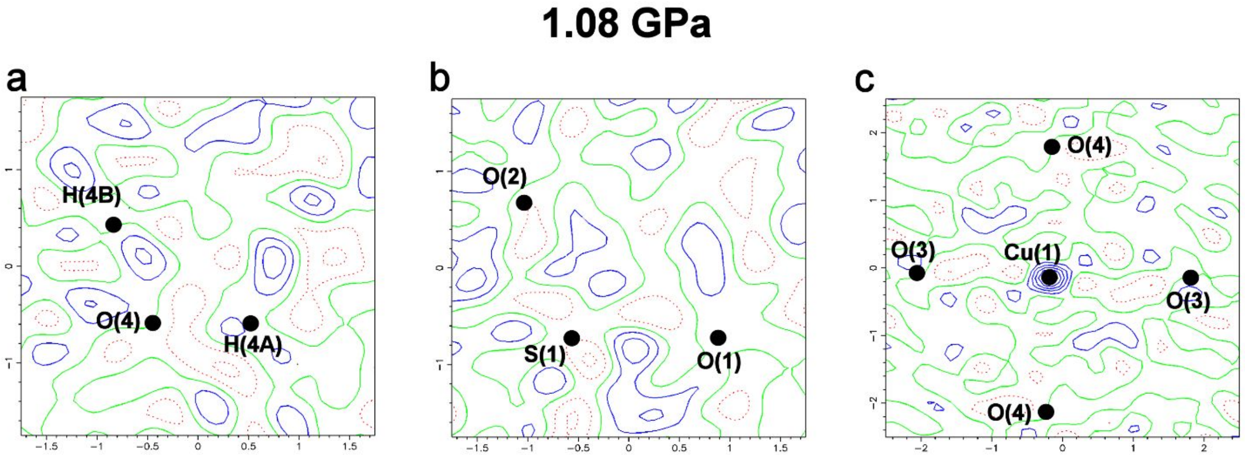

Fig. S28 2D residual electron density maps at 1.08 GPa. (a) Residual electron density map for H_2O moiety. (b) Residual electron density map for sulfate moiety. (c) Residual electron density map for CuO_6 octahedra. Positive (blue solid) and negative (red dashed) contour lines are drawn employing a step width of 0.1 $eÅ^{-3}$

1.57 GPa

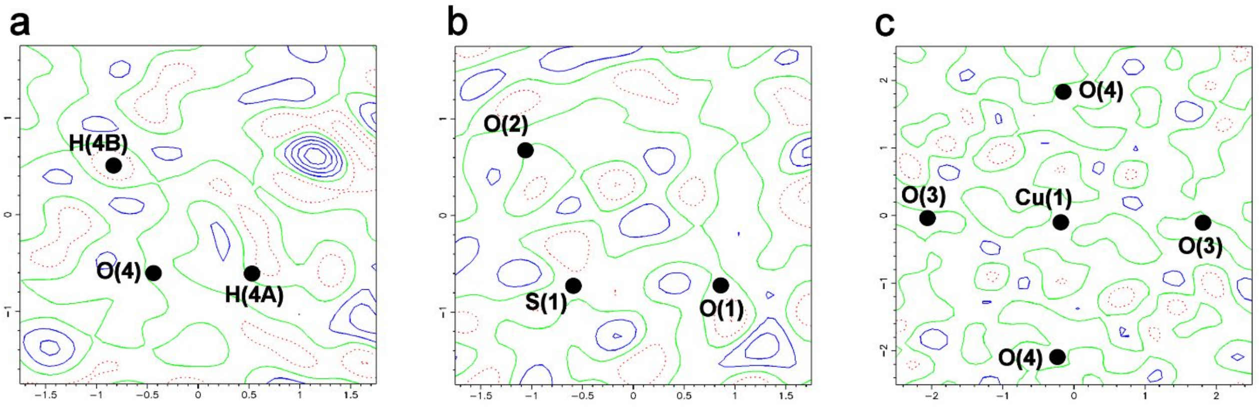

Fig. S29 2D residual electron density maps at 1.57 GPa. (a) Residual electron density map for H_2O moiety. (b) Residual electron density map for sulfate moiety. (c) Residual electron density map for CuO_6 octahedra. Positive (blue solid) and negative (red dashed) contour lines are drawn employing a step width of 0.1 eÅ^{-3}

1.99 GPa

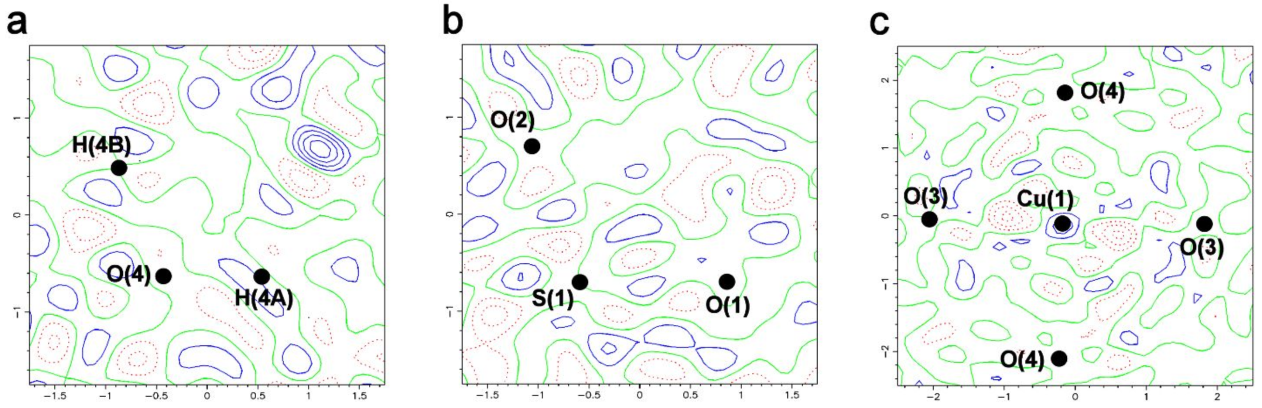

Fig. S30 2D residual electron density maps at 1.99 GPa. (a) Residual electron density map for H_2O moiety. (b) Residual electron density map for sulfate moiety. (c) Residual electron density map for CuO_6 octahedra. Positive (blue solid) and negative (red dashed) contour lines are drawn employing a step width of 0.1 eÅ^{-3}

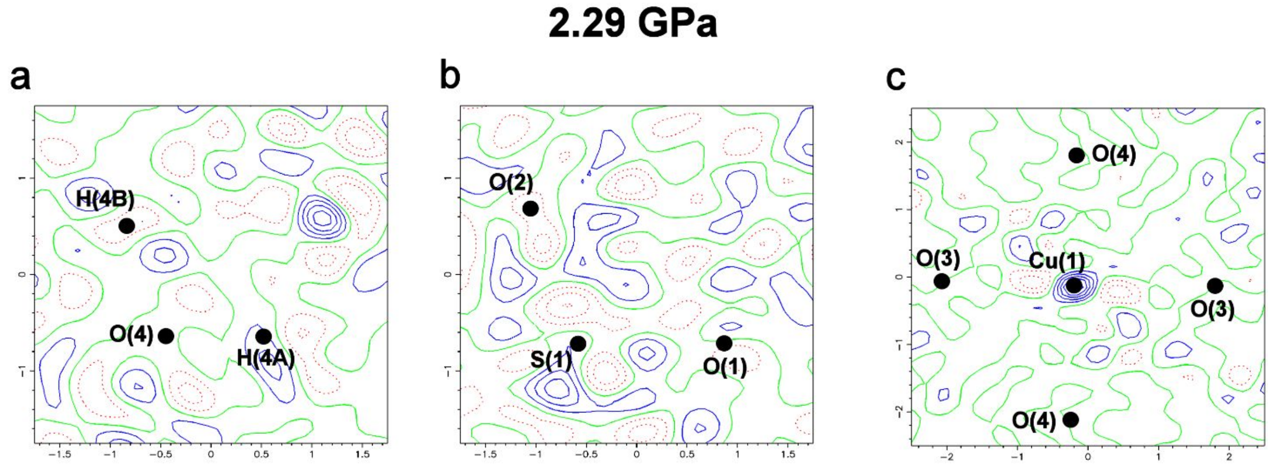

Fig. S31 2D residual electron density maps at 2.29 GPa. (a) Residual electron density map for H_2O moiety. (b) Residual electron density map for sulfate moiety. (c) Residual electron density map for CuO_6 octahedra. Positive (blue solid) and negative (red dashed) contour lines are drawn employing a step width of 0.1 $eÅ^{-3}$

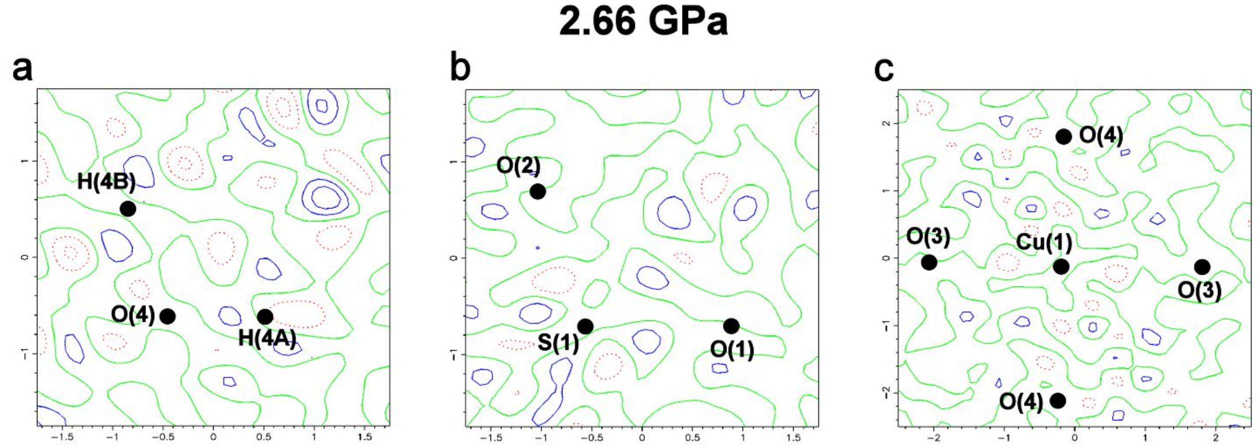

Fig. S32 2D residual electron density maps at 2.66 GPa. (a) Residual electron density map for H_2O moiety. (b) Residual electron density map for sulfate moiety. (c) Residual electron density map for CuO_6 octahedra. Positive (blue solid) and negative (red dashed) contour lines are drawn employing a step width of 0.1 $eÅ^{-3}$

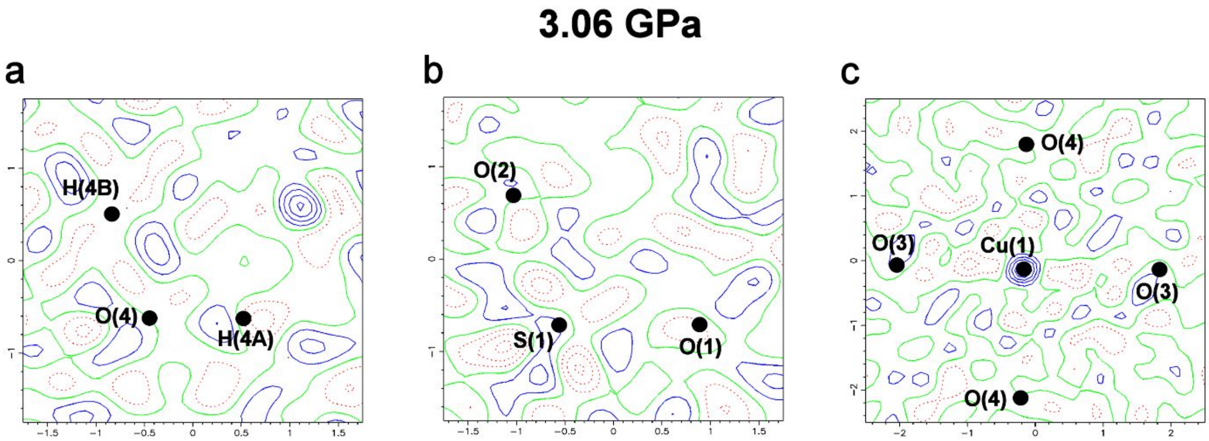

Fig. S33 2D residual electron density maps at 3.06 GPa. (a) Residual electron density map for H_2O moiety. (b) Residual electron density map for sulfate moiety. (c) Residual electron density map for CuO_6 octahedra. Positive (blue solid) and negative (red dashed) contour lines are drawn employing a step width of 0.1 eÅ^{-3}

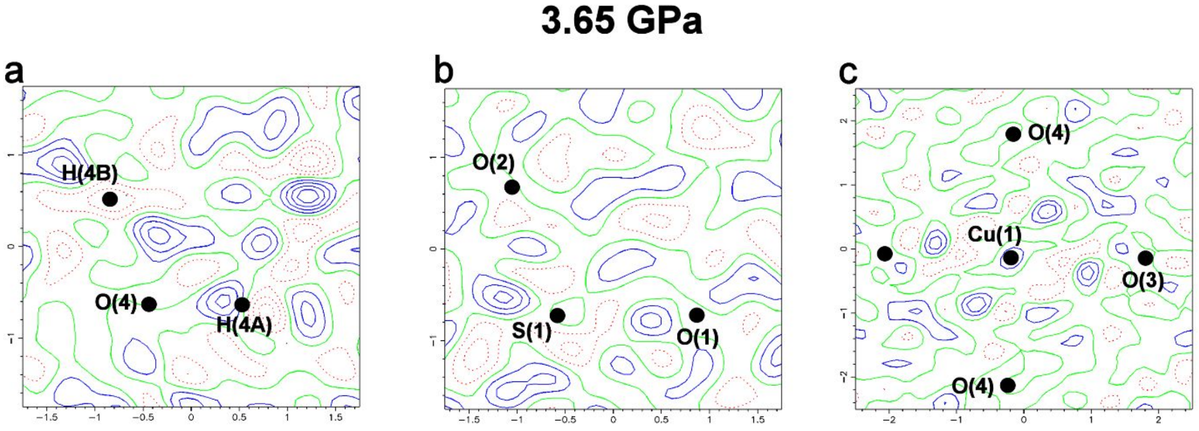

Fig. S34 2D residual electron density maps at 3.65 GPa. (a) Residual electron density map for H_2O moiety. (b) Residual electron density map for sulfate moiety. (c) Residual electron density map for CuO_6 octahedra. Positive (blue solid) and negative (red dashed) contour lines are drawn employing a step width of 0.1 eÅ^{-3}

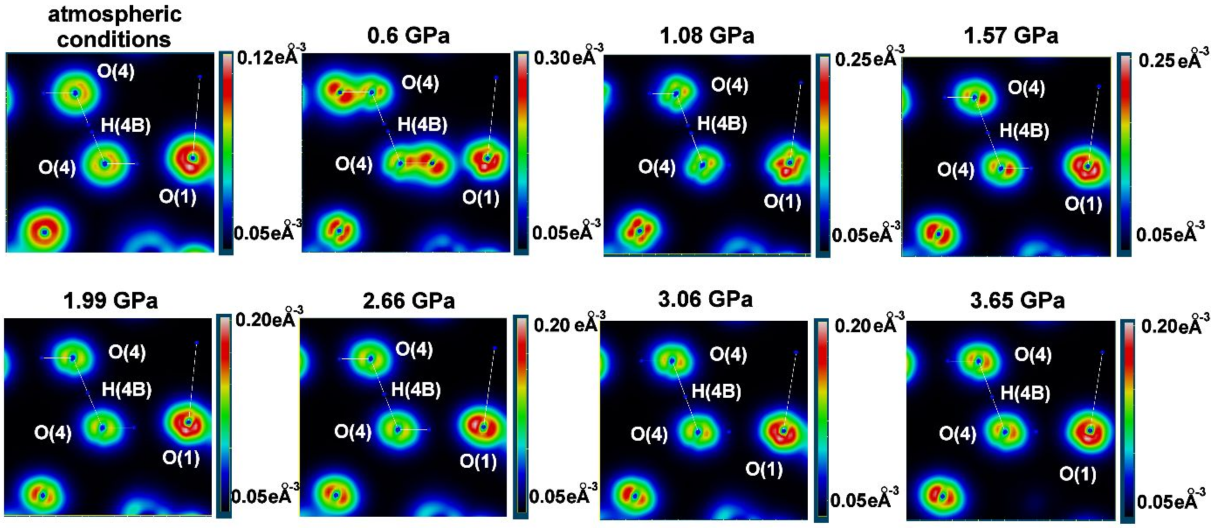

Fig. S35 Two-dimensional maps of electron density standard deviation for H_2O moiety as a function of pressure.

The correctness of the multipolar refinement was established by the analysis and evaluation of the residual density using the method proposed by Meindl and Henn.[22] The residual electron density for all measured pressure points shows an almost perfect Gaussian distribution (Fig. S19a – S26a). It means that there are no systematic features on the residual density maps and our aspherical atom models does not contain any errors nor un-modelled electron density. Additionally the values of electron density residuals are very satisfying for a high pressure experiment (up to the resolution of sin θ/λ = 1.1 $Å^{-1}$) however they are certainly higher than for the multipolar model refined against X-ray data at ambient conditions (Fig. S19b – S26b). Plots of the variation in ratio of $\Sigma(Fo^2)/\Sigma(Fc^2)$ versus sin θ/λ [23] (Fig. S19d – S26d) remain within the 5% tolerance required for a satisfactory model, free of significant data problems.[24,25] The 2D residual density maps calculated in the plane of H_2O moiety (Fig. S27a – S34a) and in the plane of sulfate moiety (Fig. S27b – S34b) did not reveal any significant peaks of un-modelled electron density in the atoms neighborhood in the whole pressure range. In the case of 2D residual density maps calculated in the plane of CuO_6 octahedra (Fig. S27c – S34c) there is visible un-modelled electron density in the position of copper atom for the models at 1.08 GPa, 2.29 GPa and 3.06 GPa. Despite using the same strategy in the multipolar refinement we were not able to model whole electron density associated with copper atom at aforementioned pressure points. It is well-known that transition metals present problems when refining electron densities because of the significantly different radial extensions of the (*n*-1)d and *n*s valence orbitals.[26] The low values for standard deviation of electron density represented on two-dimensional maps calculated parallel to the bihydroxide ion indicate the correctness of our multipole models (Fig. S35).

Section 10: Independent Atom Model and Multipole Model refinement against X-ray data - experimental tables

Table S7 Crystal data and structure refinement details for natrochalcite. For all structures: $Cu_2H_3NaO_{10}S_2$, monoclinic, *C*2/*m*, Z = 2. Experiments were carried out at 293 K. All H-atom parameters were refined.

	0.0001(GPa)<br>293(K)	0.6(GPa)<br>293 (K)	1.08(GPa)<br>293 (K)
Crystal data			
a, *b*, *c* (Å)	8.8081 (2), 6.1858 (1), 7.5073 (1)	8.7906 (5), 6.1646 (1), 7.4801 (4)	8.7851 (5), 6.15114 (13), 7.4706 (5)
β (°)	118.704 (2)	118.632 (8)	118.522 (8)
V (Å^3)	358.77 (1)	355.78 (4)	354.71 (4)
Radiation type	Mo *K*α	Synchrotron, λ= 0.2229 Å	Synchrotron, λ = 0.2229 Å
μ (mm^{-1})	6.62	0.29	0.30
Data collection			
Diffractometer	SuperNova, Single source at offset/far, Eos	Synchrotron	Synchrotron
Absorption correction	Gaussian	Multi-scan	Multi-scan
T_{min}, T_{max}	0.450, 0.893	0.836, 0.838	0.835, 0.837
No. of measured, independent and observed [$I > 2\sigma(I)$] reflections	13900, 1939, 1837	4529, 4529, 4025	4665, 4665, 4193
R_{int}	0.039	0.018	0.017
$(\sin \theta/\lambda)_{max}$ (Å^{-1})	1.069	1.554	1.555
Range of *h*, *k*, *l*	*h* = -18 →18, *k* = -13 →13, *l* = -16 →16	*h* = -23 →23, *k* = -14 →14, *l* = -20 →19	*h* = -23 →23, *k* = -13 →14, *l* = -19 →20
Refinement IAM			
$R[F^2 > 2\sigma(F^2)]$, $wR(F^2)$, S	0.025, 0.066, 1.08	0.026, 0.075, 1.00	0.028, 0.080, 0.99
No. of reflections	1939	4529	4665
No. of parameters	50	50	50
$\Delta\rho_{max}$, $\Delta\rho_{min}$ (eÅ^{-3})	0.67, -0.98	0.55, -1.01	0.84, -0.77
Refinement MM			
Refinement on, parameters, reflections	F^2/ 145/ 1892	F^2/ 145/ 2277	F^2/ 145/ 2312
$R[F^2 > 2\sigma(F^2)]$, R(all)	0.023, 0.023	0.025, 0.032	0.027, 0.034
$wR[F^2 > 2\sigma(F^2)]$	0.051	0.059	0.075
$(\sin \theta/\lambda)_{max}$ (Å^{-1})	1.11	1.11	1.11
$\Delta\rho_{max}$, $\Delta\rho_{min}$ (eÅ^{-3})	0.544, -0.689	0.378, -0.353	0.463, -0.404

Table S8 Crystal data and structure refinement details for natrochalcite. For all structures: $Cu_2H_3NaO_{10}S_2$, monoclinic, *C*2/*m*, Z = 2. Experiments were carried out at 293 K. All H-atom parameters were refined.

	1.57(GPa)<br>293 (K)	1.99(GPa)<br>293 (K)	2.29(GPa)<br>293 (K)
Crystal data			
a, *b*, *c* (Å)	8.7562 (3), 6.1223 (1), 7.4340 (2)	8.7413 (5), 6.1026 (2), 7.4168 (6)	8.7303 (4), 6.09060 (14), 7.4032 (5)
β (°)	118.445 (4)	118.379 (9)	118.333 (7)
V (Å^3)	350.41 (2)	348.10 (4)	346.49 (4)
Radiation type	Synchrotron, λ = 0.2229 Å	Synchrotron, λ = 0.2229 Å	Synchrotron, λ = 0.2229 Å
μ (mm^{-1})	0.30	0.30	0.30
Data collection			
Diffractometer	Synchrotron	Synchrotron	Synchrotron
Absorption correction	Multi-scan	Multi-scan	Multi-scan
T_{min}, T_{max}	0.834, 0.836	0.833, 0.835	0.832, 0.834
No. of measured, independent and observed [$I > 2\sigma(I)$] reflections	4316, 4316, 3843	4426, 4426, 3993	4494, 4494, 4105
R_{int}	0.019	0.017	0.017
(sin θ/λ)$_{max}$ (Å^{-1})	1.534	1.530	1.540
Range of *h*, *k*, *l*	*h* = -23 →23, *k* = -14 →13, *l* = -20 →19	*h* = -23 →24, *k* = -14 →13, *l* = -20 →19	*h* = -24 →24, *k* = -14 →13, *l* = -18 →20
Refinement IAM			
$R[F^2 > 2\sigma(F^2)]$, $wR(F^2)$, *S*	0.024, 0.069, 1.02	0.027, 0.081, 1.02	0.026, 0.075, 1.03
No. of reflections	4316	4426	4494
No. of parameters	50	50	50
$\Delta\rho_{max}$, $\Delta\rho_{min}$ (eÅ^{-3})	0.85, -1.20	0.95, -1.26	0.91, -1.26
Refinement MM			
Refinement on, parameters, reflections	F^2/ 145/ 2057	F^2/ 145/ 2251	F^2/ 145/ 2304
R[$F^2 > 2\sigma (F^2)$], R(all)	0.02, 0.034	0.023, 0.034	0.023, 0.033
wR[$F^2 > 2\sigma (F^2)$]	0.049	0.057	0.064
(sin θ/λ)$_{max}$ (Å^{-1})	1.11	1.11	1.11
$\Delta\rho_{max}$, $\Delta\rho_{min}$ (eÅ^{-3})	0.535, -0.3	0.544, -0.404	0.469, -0.368

Table S9 Crystal data and structure refinement details for natrochalcite. For all structures: $Cu_2H_3NaO_{10}S_2$, monoclinic, *C*2/*m*, Z = 2. Experiments were carried out at 293 K. All H-atom parameters were refined.

	2.66(GPa)<br>293 (K)	3.06(GPa)<br>293 (K)	3.65(GPa)<br>293 (K)
Crystal data			
a, *b*, *c* (Å)	8.7211 (4), 6.0786 (1), 7.3911 (4)	8.7075 (4), 6.06280 (12), 7.3749 (4)	8.6883 (4), 6.0408 (1), 7.3520 (4)
β (°)	118.271 (7)	118.202 (7)	118.113 (7)
V (Å^3)	345.08 (3)	343.12 (3)	340.34 (3)
Radiation type	Synchrotron, λ = 0.2229 Å	Synchrotron, λ = 0.2229 Å	Synchrotron, λ = 0.2229 Å
μ (mm^{-1})	0.30	0.30	0.31
Data collection			
Diffractometer	Synchrotron	Synchrotron	Synchrotron
Absorption correction	Multi-scan	Multi-scan	Multi-scan
T_{min}, T_{max}	0.832, 0.834	0.831, 0.833	0.829, 0.832
No. of measured, independent and observed [$I > 2\sigma(I)$] reflections	4255, 4255, 3844	4328, 4328, 3945	4372, 4372, 3990
R_{int}	0.015	0.016	0.015
(sin θ/λ)$_{max}$ (Å^{-1})	1.540	1.533	1.536
Range of *h*, *k*, *l*	*h* = -24 →24, *k* = -14 →13, *l* = -18 →19	*h* = -24 →24, *k* = -14 →13, *l* = -19 →19	*h* = -24 →24, *k* = -14 →13, *l* = -19 →18
Refinement IAM			
$R[F^2 > 2\sigma(F^2)]$, $wR(F^2)$, S	0.027, 0.078, 1.02	0.023, 0.070, 1.03	0.026, 0.077, 1.01
No. of reflections	4255	4328	4372
No. of parameters	50	50	50
$\Delta\rho_{max}$, $\Delta\rho_{min}$ (eÅ^{-3})	0.82, -1.21	0.68, -1.16	0.73, -1.15
Refinement MM			
Refinement on, parameters, reflections	F^2/ 145/ 2097	F^2/ 145/ 2267	F^2/ 145/ 2306
R[F^2 > 2σ (F^2)], R(all)	0.024, 0.031	0.022, 0.029	0.024, 0.03
wR[F^2 > 2σ (F^2)]	0.057	0.057	0.058
(sin θ/λ)$_{max}$ (Å^{-1})	1.11	1.11	1.11
$\Delta\rho_{max}$, $\Delta\rho_{min}$ (eÅ^{-3})	0.775, -0.665	0.455, -0.379	0.52, -0.446

Table S10 Crystal data and structure refinement details for natrochalcite: $Cu_2H_3NaO_{10}S_2$, monoclinic, *C*2/*m*, Z = 2. Experiment was carried out at 100 K.

	0.0001(GPa)<br>100(K)
Crystal data	
a, *b*, *c* (Å)	8.7853 (5), 6.1706 (2), 7.4842 (4)
β (°)	118.742 (7)
V ($Å^3$)	355.74 (4)
Radiation type	Mo *K*α
μ (mm^{-1})	6.67
Data collection	
Diffractometer	SuperNova, Single source at offset/far, Eos
Absorption correction	Multi-scan
T_{min}, T_{max}	0.467, 1.000
No. of measured, independent and observed [$I > 2σ(I)$] reflections	2701, 723, 670
R_{int}	0.060
$(\sin θ/λ)_{max}$ ($Å^{-1}$)	0.769
Range of *h*, *k*, *l*	*h* = -13 →12, *k* = -9 →9, *l* = -11 →11
Refinement IAM	
$R[F^2 > 2σ(F^2)]$, $wR(F^2)$, *S*	0.034, 0.089, 1.05
No. of reflections	723
No. of parameters	44
$Δρ_{max}$, $Δρ_{min}$ ($eÅ^{-3}$)	1.37, -1.02

Section 11: Experimental tables for single crystal data collected using electron and neutron diffraction methods

Table S11 Crystal data and structure refinement details against single crystal neutron diffraction data for natrochalcite: $Cu_2H_3NaO_{10}S_2$, monoclinic, *C*2/*m*, Z = 2.

	1.6(GPa)<br>300(K)
Crystal data	
a, *b*, *c* (Å)	8.7562 (3), 6.1223 (1), 7.4340 (2)
β (°)	118.445 (4)
V (Å^3)	350.41 (2)
Radiation type	Neutrons, λ = 1.003 Å
μ (mm^{-1})	17.13
Data collection	
Diffractometer	DEMAND
T_{min}, T_{max}	0.834, 0.836
No. of measured, independent and observed [$I > 2\sigma(I)$] reflections	182, 182, 153
$(\sin \theta/\lambda)_{max}$ (Å^{-1})	0.621
Refinement	
$R[F^2 > 2\sigma(F^2)]$, $wR(F^2)$, S	0.107, 0.263, 1.10
No. of reflections	182
No. of parameters	36
H-atom treatment	All H-atom parameters refined
$\Delta\rho_{max}$, $\Delta\rho_{min}$ (eÅ^{-3})	1.37, -1.34

Table S12 Crystal data and structure refinements details against single crystal electron diffraction data for natrochalcite: $Cu_2H_3NaO_{10}S_2$, monoclinic, *C*2/*m*, Z = 2.

	0.0001(GPa)
	80(K)
Crystal data	
a, *b*, *c* (Å)	8.5358 (18), 6.114 (2), 7.3701 (16)
β (°)	118.04 (3)
V (Å^3)	339.46 (18)
Radiation type	Electron, λ = 0.0251 Å
Data collection	
Diffractometer	TEM Thermofisher 200 kV Glacios^{TM} FEG Cryo
3D ED method	Continuous Rotation
T_{min}, T_{max}	0.867, 1.000
No. of measured, independent and observed [$I > 2\sigma(I)$] reflections	3647, 1502, 637
Tilt range, tilt step	-60° to +60°, 0.5° -
Exposure time per frame (ms)	1000
Completeness	94.1%
R_{int}	0.180
$(\sin \theta/\lambda)_{max}$ (Å^{-1})	1.229
IAM Kinematical Refinement	
$R[F^2 > 2\sigma(F^2)]$, $wR(F^2)$, S	0.188, 0.507, 1.28
No. of reflections	1502
No. of parameters	45
$\Delta\rho_{max}$, $\Delta\rho_{min}$ (eÅ^{-3})	0.47, -0.78
IAM Dynamical Refinement	
RSg(max), DSg(max)	0.66, 0.00
No. of reflections (Nobs/ Nall)	2065/8181
No. of parameters	85
R(obs)/R(all)	10.19/19.63
GoF	1.521
Kappa Refinement	
RSg(max), DSg(max)	0.66, 0.00
No. of reflections (Nobs/ Nall)	2065/8181
No. of parameters	101
R(obs)/R(all)	5.63/19.59
GoF	0.934

Section 12: Completeness of the collected data at high pressure conditions

Table S13 Distribution of measured and missing reflections in equal-volume resolution shells at 0.6 GPa.

shell $\sin\theta_{max}/\lambda$ (Å^{-1})	shell d_{min} (Å)	hkl measured	hkl missing	percent completeness
0.4091	1.222	74	41	64.3
0.5155	0.970	79	39	66.9
0.5901	0.847	76	30	71.7
0.6494	0.770	72	34	67.9
0.6996	0.715	83	33	71.6
0.7434	0.673	63	34	64.9
0.7826	0.639	79	34	69.9
0.8183	0.611	72	34	67.9
0.8510	0.588	75	41	64.7
0.8814	0.567	65	32	67.0
0.9099	0.550	69	40	63.3
0.9367	0.534	72	37	66.1
0.9620	0.520	69	31	69.0
0.9861	0.507	73	43	62.9
1.0090	0.496	60	36	62.5
1.0309	0.485	67	44	60.4
1.0520	0.475	77	34	69.4
1.0722	0.466	57	44	56.4
1.0917	0.458	77	38	67.0
1.1105	0.450	52	39	57.1

Table S14 Distribution of measured and missing reflections in equal-volume resolution shells at 1.08 GPa.

shell $\sin\theta_{max}/\lambda$ (Å^{-1})	shell d_{min} (Å)	hkl measured	hkl missing	percent completeness
0.4094	1.221	72	43	62.6
0.5158	0.969	77	40	65.8
0.5904	0.847	71	36	66.4
0.6499	0.769	69	35	66.3
0.7000	0.714	73	43	62.9
0.7439	0.672	62	35	63.9
0.7831	0.638	74	39	65.5
0.8188	0.611	67	38	63.8
0.8516	0.587	65	50	56.5
0.8820	0.567	63	34	64.9
0.9105	0.549	71	37	65.7
0.9373	0.533	60	47	56.1
0.9626	0.519	61	40	60.4
0.9867	0.507	64	46	58.2
1.0096	0.495	61	43	58.7
1.0316	0.485	60	46	56.6
1.0527	0.475	73	40	64.6
1.0729	0.466	55	45	55.0
1.0924	0.458	69	47	59.5
1.1112	0.45	41	50	45.1

Table S15 Distribution of measured and missing reflections in equal-volume resolution shells at 1.57 GPa.

shell $\sin\theta_{max}/\lambda$ (Å^{-1})	shell d_{min} (Å)	hkl measured	hkl missing	percent completeness
0.4092	1.222	77	38	67.0
0.5155	0.970	74	41	64.3
0.5901	0.847	76	31	71.0
0.6495	0.770	70	33	68.0
0.6997	0.715	73	39	65.2
0.7435	0.672	61	34	64.2
0.7827	0.639	78	37	67.8
0.8184	0.611	71	35	67.0
0.8511	0.587	68	43	61.3
0.8816	0.567	66	32	67.3
0.9100	0.549	68	37	64.8
0.9368	0.534	62	40	60.8
0.9621	0.520	73	36	67.0
0.9862	0.507	65	38	63.1
1.0091	0.495	70	41	63.1
1.0311	0.485	59	37	61.5
1.0521	0.475	71	40	64.0
1.0724	0.466	74	35	67.9
1.0919	0.458	57	44	56.4
1.1107	0.450	62	41	60.2

Table S16 Distribution of measured and missing reflections in equal-volume resolution shells at 1.99 GPa.

shell $\sin\theta_{max}/\lambda$ (Å^{-1})	shell d_{min} (Å)	hkl measured	hkl missing	percent completeness
0.4096	1.221	79	36	68.7
0.5161	0.969	79	36	68.7
0.5907	0.846	79	26	75.2
0.6502	0.769	73	30	70.9
0.7004	0.714	78	33	70.3
0.7443	0.672	69	28	71.1
0.7835	0.638	76	33	69.7
0.8192	0.610	75	30	71.4
0.8520	0.587	73	39	65.2
0.8825	0.567	70	31	69.3
0.9109	0.549	73	33	68.9
0.9377	0.533	63	33	65.6
0.9631	0.519	78	33	70.3
0.9872	0.506	71	33	68.3
1.0102	0.495	72	41	63.7
1.0321	0.484	59	32	64.8
1.0532	0.475	71	39	64.5
1.0735	0.466	74	35	67.9
1.0930	0.457	61	38	61.6
1.1118	0.450	61	40	60.4

Table S17 Distribution of measured and missing reflections in equal-volume resolution shells at 2.29 GPa.

shell $\sin\theta_{max}/\lambda$ (Å^{-1})	shell d_{min} (Å)	hkl measured	hkl missing	percent completeness
0.4091	1.222	77	37	67.5
0.5155	0.970	75	37	67.0
0.5901	0.847	80	26	75.5
0.6495	0.770	77	28	73.3
0.6996	0.715	76	33	69.7
0.7435	0.673	70	28	71.4
0.7827	0.639	76	32	70.4
0.8183	0.611	73	30	70.9
0.8511	0.588	78	31	71.6
0.8815	0.567	74	31	70.5
0.9099	0.549	69	31	69.0
0.9367	0.534	62	37	62.6
0.9620	0.520	84	28	75.0
0.9861	0.507	71	33	68.3
1.0090	0.496	72	38	65.5
1.0310	0.485	56	29	65.9
1.0520	0.475	77	36	68.1
1.0723	0.466	75	30	71.4
1.0918	0.458	68	37	64.8
1.1106	0.450	63	45	58.3

Table S18 Distribution of measured and missing reflections in equal-volume resolution shells at 2.66 GPa.

shell $\sin\theta_{max}/\lambda$ (Å^{-1})	shell d_{min} (Å)	hkl measured	hkl missing	percent completeness
0.4101	1.219	78	37	67.8
0.5167	0.968	77	37	67.5
0.5915	0.845	82	22	78.8
0.6510	0.768	79	27	74.5
0.7012	0.713	78	29	72.9
0.7452	0.671	71	27	72.4
0.7845	0.637	82	27	75.2
0.8202	0.610	80	26	75.5
0.8530	0.586	77	30	72.0
0.8835	0.566	76	30	71.7
0.9120	0.548	71	30	70.3
0.9389	0.533	67	30	69.1
0.9643	0.519	84	29	74.3
0.9884	0.506	75	29	72.1
1.0114	0.494	73	35	67.6
1.0334	0.484	56	32	63.6
1.0545	0.474	79	36	68.7
1.0747	0.465	81	28	74.3
1.0943	0.457	66	33	66.7
1.1132	0.449	65	40	61.9

Table S19 Distribution of measured and missing reflections in equal-volume resolution shells at 3.06 GPa.

shell $\sin\theta_{max}/\lambda$ (Å^{-1})	shell d_{min} (Å)	hkl measured	hkl missing	percent completeness
0.4091	1.222	76	37	67.3
0.5155	0.970	79	32	71.2
0.5900	0.847	82	24	77.4
0.6494	0.770	77	27	74.0
0.6996	0.715	75	32	70.1
0.7434	0.673	67	26	72.0
0.7826	0.639	84	31	73.0
0.8182	0.611	78	27	74.3
0.8510	0.588	65	31	67.7
0.8814	0.567	82	22	78.8
0.9099	0.550	73	34	68.2
0.9366	0.534	65	34	65.7
0.9620	0.520	81	30	73.0
0.9860	0.507	72	29	71.3
1.0090	0.496	73	35	67.6
1.0309	0.485	54	29	65.1
1.0519	0.475	76	39	66.1
1.0722	0.466	72	26	73.5
1.0917	0.458	68	34	66.7
1.1105	0.450	74	36	67.3

Table S20 Distribution of measured and missing reflections in equal-volume resolution shells at 3.65 GPa.

shell $\sin\theta_{max}/\lambda$ (Å^{-1})	shell d_{min} (Å)	hkl measured	hkl missing	percent completeness
0.4095	1.221	76	34	69.1
0.5159	0.969	79	34	69.9
0.5906	0.847	78	25	75.7
0.6500	0.769	77	26	74.8
0.7002	0.714	76	31	71.0
0.7441	0.672	72	25	74.2
0.7833	0.638	79	31	71.8
0.8190	0.611	77	22	77.8
0.8518	0.587	62	33	65.3
0.8822	0.567	85	26	76.6
0.9107	0.549	72	34	67.9
0.9375	0.533	69	33	67.6
0.9628	0.519	78	28	73.6
0.9869	0.507	72	29	71.3
1.0099	0.495	67	35	65.7
1.0318	0.485	59	30	66.3
1.0529	0.475	72	38	65.5
1.0732	0.466	79	25	76.0
1.0927	0.458	66	32	67.3
1.1115	0.450	67	42	61.5

Section 13: References

(1) Gajda, R.; Zhang, D.; Parafiniuk, J.; Dera, P.; Woźniak, K. W. Tracing Electron Density Changes in Langbeinite under Pressure. https://doi.org/10.1107/S2052252521012628.

(2) Stachowicz, M.; Gajda, R.; Huć, A.; Parafiniuk, J.; Makal, A.; Sutuła, S.; Fertey, P.; Woźniak, K. Charge Density Redistribution with Pressure in a Zeolite Framework. *Scientific Reports 2023 13:1* **2023**, *13* (1), 1–9. https://doi.org/10.1038/s41598-023-28350-4.

(3) Casati, N.; Genoni, A.; Meyer, B.; Krawczuk, A.; MacChi, P. Exploring Charge Density Analysis in Crystals at High Pressure: Data Collection, Data Analysis and Advanced Modelling. *Acta Crystallogr B Struct Sci Cryst Eng Mater* **2017**, *73* (4), 584–597. https://doi.org/10.1107/S2052520617008356/LC5086SUP3.CIF.

(4) Koritsanszky, T. S.; Coppens, P. Chemical Applications of X-Ray Charge-Density Analysis. *Chem Rev* **2001**, *101* (6), 1583–1627. https://doi.org/10.1021/CR990112C/ASSET/IMAGES/LARGE/CR990112CH00023.JPEG.

(5) Hansen, N. K.; Coppens, P. Testing Aspherical Atom Refinements on Small-Molecule Data Sets. *Acta Cryst* **1978**, *34* (6), 909–921. https://doi.org/10.1107/S0567739478001886.

(6) Clementi, E.; Roetti, C. Roothaan-Hartree-Fock Atomic Wavefunctions: Basis Functions and Their Coefficients for Ground and Certain Excited States of Neutral and Ionized Atoms, Z≤54. *At Data Nucl Data Tables* **1974**, *14* (3–4), 177–478. https://doi.org/10.1016/S0092-640X(74)80016-1.

(7) Chevrier, G.; Giester, G.; Zemann, J. Neutron refinements of $NaCu_2(H_3O_2)(SO_4)_2$ and $RbCu_2(H_3O_2)(SeO_4)_2$: variation of the hydrogen-bond system in the natrochalcite-type series. *Zeitschrift für Kristallographie* **1993**, 206, 7–14.

(8) Hirshfeld, F. L. Bonded-Atom Fragments for Describing Molecular Charge Densities. *Theor Chim Acta* **1977**, *44* (2), 129–138. https://doi.org/10.1007/BF00549096/METRICS.

(9) R. F. W. Bader. Atoms in Molecules: A Quantum Theory, Oxford University Press. **1990**, 438.

(10) Gatti, C. Chemical Bonding in Crystals: New Directions. *Zeitschrift für Kristallographie* **2005**, *220* (5–6), 399–457. https://doi.org/10.1524/ZKRI.220.5.399.65073/MACHINEREADABLECITATION/RIS.

(11) Popelier, P. L. A. Integration of Atoms in Molecules: A Critical Examination. *Mol Phys* **1996**, *87* (5), 1169–1187. https://doi.org/10.1080/00268979600100781.

(12) Coppens, P.; Guru Row, T. N.; Leung, P.; Stevens, E. D.; Becker, P. J.; Yang, Y. W. Net Atomic Charges and Molecular Dipole Moments from Spherical-Atom X-Ray Refinements, and the Relation between Atomic Charge and Shape. *urn:issn:0567-7394* **1979**, *35* (1), 63–72. https://doi.org/10.1107/S0567739479000127.

(13) Ángyán, J. G.; Jansen, G.; Loss, M.; Hättig, C.; Heß, B. A. Distributed Polarizabilities Using the Topological Theory of Atoms in Molecules. *Chem Phys Lett* **1994**, *219* (3–4), 267–273. https://doi.org/10.1016/0009-2614(94)87056-X.

(14) Dovesi, R.; Erba, A.; Orlando, R.; Zicovich-Wilson, C. M.; Civalleri, B.; Maschio, L.; Rérat, M.; Casassa, S.; Baima, J.; Salustro, S.; Kirtman, B. Quantum-Mechanical Condensed Matter Simulations with CRYSTAL. *Wiley Interdiscip Rev Comput Mol Sci* **2018**, *8* (4), e1360. https://doi.org/10.1002/WCMS.1360.

(15) Dovesi, R.; Orlando, R.; Civalleri, B.; Roetti, C.; Saunders, V. R.; Zicovich-Wilson, C. M. CRYSTAL: A Computational Tool for the Ab Initio Study of the Electronic Properties of Crystals. *Zeitschrift für Kristallographie* **2005**, *220* (5–6), 571–573. https://doi.org/10.1524/ZKRI.220.5.571.65065/MACHINEREADABLECITATION/RIS.
(16) Erba, A.; Ferrabone, M.; Orlando, R.; Dovesi, R. Accurate Dynamical Structure Factors from Ab Initio Lattice Dynamics: The Case of Crystalline Silicon. *J Comput Chem* **2013**, *34* (5), 346–354. https://doi.org/10.1002/JCC.23138.
(17) Lee, C.; Yang, W.; Parr, R. G. Development of the Colle-Salvetti Correlation-Energy Formula into a Functional of the Electron Density. *Phys Rev B Condens Matter* **1988**, *37* (2), 785–789. https://doi.org/10.1103/PHYSREVB.37.785.
(18) Becke, A. D.; Chem Phys, J. Density-functional Thermochemistry. III. The Role of Exact Exchange. *J Chem Phys* **1993**, *98* (7), 5648–5652. https://doi.org/10.1063/1.464913.
(19) Grimme, S.; Antony, J.; Ehrlich, S.; Krieg, H. A Consistent and Accurate Ab Initio Parametrization of Density Functional Dispersion Correction (DFT-D) for the 94 Elements H-Pu. *Journal of Chemical Physics* **2010**, *132* (15), 154104. https://doi.org/10.1063/1.3382344/926936.
(20) Vilela Oliveira, D.; Laun, J.; Peintinger, M. F.; Bredow, T. BSSE-Correction Scheme for Consistent Gaussian Basis Sets of Double- and Triple-Zeta Valence with Polarization Quality for Solid-State Calculations. *J Comput Chem* **2019**, *40* (27), 2364–2376. https://doi.org/10.1002/JCC.26013.
(21) Ruiz, E.; Llunell, M.; Alemany, P. Calculation of Exchange Coupling Constants in Solid State Transition Metal Compounds Using Localized Atomic Orbital Basis Sets. *J Solid State Chem* **2003**, *176* (2), 400–411. https://doi.org/10.1016/S0022-4596(03)00238-X.
(22) Meindl, K.; Henn, J. Foundations of Residual-Density Analysis. *Acta Cryst* **2008**, *64* (3), 404–418. https://doi.org/10.1107/S0108767308006879.
(23) Zhurov, V. V.; Zhurova, E. A.; Pinkerton, A. A. Optimization and Evaluation of Data Quality for Charge Density Studies. *J Appl Crystallogr* **2008**, *41* (2), 340–349. https://doi.org/10.1107/S0021889808004482/DO5038SUP1.PDF.
(24) Herbst-Irmer, R.; Stalke, D. Experimental Charge-Density Studies: Data Reduction and Model Quality: The More the Better? *Acta Cryst* B **2017**, *73* (4), 531–543. https://doi.org/10.1107/S2052520617007016.
(25) Saunders, L. K.; Pallipurath, A. R.; Gutmann, M. J.; Nowell, H.; Zhang, N.; Allan, D. R. A Quantum Crystallographic Approach to Short Hydrogen Bonds. *CrystEngComm* **2021**, *23* (35), 6180–6190. https://doi.org/10.1039/D1CE00355K.
(26) Dos Santos, L. H. R.; Lanza, A.; Barton, A. M.; Brambleby, J.; Blackmore, W. J. A.; Goddard, P. A.; Xiao, F.; Williams, R. C.; Lancaster, T.; Pratt, F. L.; Blundell, S. J.; Singleton, J.; Manson, J. L.; Macchi, P. Experimental and Theoretical Electron Density Analysis of Copper Pyrazine Nitrate Quasi-Low-Dimensional Quantum Magnets. *J Am Chem Soc* **2016**, *138* (7), 2280–2291. https://doi.org/10.1021/JACS.5B12817/SUPPL_FILE/JA5B12817_SI_002.CIF.
